# Efficacy, Immunogenicity, and Safety of Enterovirus 71 Vaccines in Children: A Systematic Review and Meta-Analysis

**DOI:** 10.3390/vaccines14030235

**Published:** 2026-03-04

**Authors:** Guan Xing Lai, Albert Ka Wing Au, Edmond Siu Keung Ma

**Affiliations:** 1 Department of Health, Centre for Health Protection, Communicable Disease Branch, Hong Kong SAR, China; 2 Department of Health, Centre for Health Protection, Infection Control Branch, Hong Kong SAR, China; edmond_sk_ma@dh.gov.hk

**Keywords:** hand, foot and mouth disease (HFMD), enterovirus 71 (EV71), vaccine

## Abstract

**Background/Objectives**: Hand, foot and mouth disease (HFMD) caused by enterovirus 71 (EV71) may cause severe complications and death in children. It is also a common cause of outbreaks in the Asia-Pacific Region. Incidence among children 1 to <2 years was over 3000/100,000 population in China. A systematic review and meta-analysis was performed to review evidence on vaccine efficacy (VE), immunogenicity, and safety of two doses of EV71 vaccine in children. **Methods**: Randomized controlled trials (RCTs) comparing EV71 vaccine with placebo or with another EV71 vaccine in children and adolescents aged ≤18 years were searched on PubMed, Medline, Embase, CENTRAL, and CNKI (Chinese) in week 5 November 2024. The reference list of each study and the websites of vaccine manufacturers were also searched. The Cochrane Risk of Bias 2 tool (RoB2) was used to assess the risk of bias. VE, immunogenicity (including seropositive rate, seroconversion rate, geometric mean titer (GMT), Geometric Mean Fold Increase (GMFI)), and rate of adverse events were analyzed. **Results**: A total of 4199 articles were identified, and 25 studies were finally included. VE (%) against EV71 HFMD in children aged ≤5 years at 12 months was 94.8% (95%CI 87.2–97.9) for Sinovac and 90.9% (95%CI 70.4–97.2) for Wuhan Institute of Biological Products (WIBP), while the Chinese Academy of Medical Sciences (CAMS) reported 97.4% (95%CI 92.9–99.0) at 11 months. At 1 month after the second dose, 99.19% (95%CI 98.15–99.65) of children aged ≤5 years in the vaccine group were seropositive, and 96.30% (95%CI 92.71–98.17) achieved seroconversion. GMT at 1 month after the second dose in the vaccine group was 46.78 (95%CI 26.18–83.61) times that in the placebo group. GMFI at 1 month after the second dose in the vaccine group was 28.41 (95%CI 22.18–36.39) times that of the placebo group. The rate of serious adverse events (AEs) was lower in the vaccine group than the placebo group (1.23% (95%CI 0.58–2.69) vs. 1.34% (95%CI 0.58–3.07)) at 1 month after the second dose. There was no significant difference in other adverse events between the vaccine and placebo groups. **Conclusions**: EV71 vaccines were effective, immunogenic and safe. Areas with a high incidence of EV71 may consider introducing EV71 vaccines.

## 1. Introduction

According to the World Health Organization (WHO), hand, foot and mouth disease (HFMD) is a ‘febrile illness with papulovesicular rash on palms and soles, with or without vesicles/ulcers in the mouth’. HFMD is caused by enteroviruses, mainly affecting children. It is transmitted by fecal–oral and respiratory secretions [1]. HFMD is mostly self-limiting and resolves in 7–10 days [2]. However, Enterovirus 71 (EV71) infection is of particular concern since it may cause severe complications in children, especially those aged ≤5 [3]. According to the World Health Organization, approximately 10–30% of hospitalized cases during EV71-associated HFMD epidemics in Asia developed central nervous system complications, including aseptic meningitis, encephalitis and acute flaccid paralysis. Fatal cases often developed acute refractory myocardial dysfunction and fulminant pulmonary oedema [1]. Long-term sequelae were related to greater clinical severity at the acute stage, neurological damage and younger age of onset. These may include: dysphagia requiring tube feeding, central hypoventilation with ventilator support, neurodevelopmental delay, impaired cognition, seizure, limb weakness and behavioral problems [4]. EV71 infection also caused significant economic loss. A USA study published in 1998 estimated direct medical costs of USD$69–771/case and indirect costs of USD$63–422/case, mainly attributable to parental missed work [5,6]. Another Chinese study published in 2017 estimated medical costs of USD$88–2501/case and indirect costs of USD$113–677/case [7]. The Asia-Pacific region had cyclical EV71 outbreaks affecting Malaysia, China, and Vietnam, with major outbreaks occurring every 3–4 years [8,9,10]. From 2008 to 2017, around 18 million HFMD cases were reported in China (incidence = 134.59/100,000 population), 151,194 (0.85%) of which were severe, and most of those (89.66%) were children < 5 years old; 3623 (0.02%) of which died, and most of those (97.96%) were children < 5 years old. Incidence in children < 5 was much higher than that of the general population: incidence at age < 1 years, 1 to <2 years, 2 to <3 years, 3 to <4 years and 4 to <5 years was 1084, 3184, 2547, 2052 and 1151 per 100,000 population, respectively. Among laboratory-confirmed HFMD cases, 42.97% were caused by EV71 [11]. Management of HFMD is mainly supportive, and no antivirals are available. In places without the EV71 vaccine, the mainstay of prevention of HFMD is maintaining personal and environmental hygiene. Control measures of school outbreaks include strengthening infection control, excluding sick children from school, or even school suspension. Although these public health measures are shown to be effective in controlling HFMD [12], vaccination is considered of utmost importance in reducing the disease burden.

The first EV71 vaccine was approved in the Chinese Mainland in December 2015 [13]. Currently, there are five vaccines approved worldwide, with all approved in China (three in the Chinese Mainland, and two in Taiwan, China) (Table 1). All approved vaccines are inactivated vaccines. The three vaccines approved in the Chinese Mainland were developed from sub-genotype C4 and approved for use in children aged 6–71 months. The remaining two vaccines approved in Taiwan, China, were developed from sub-genotype B4 and approved for use in children aged 2–71 months. The interval between doses is 28 days for all vaccines except Medigen. We aimed to review evidence on vaccine efficacy (VE), immunogenicity, and safety of two doses of EV71 vaccine in children.

## 2. Methods

This study was conducted following the Preferred Reporting Items for Systematic reviews and Meta-Analyses (PRISMA) guideline [24]. This review was registered with PROSPERO (registration number: CRD420261306764).

### 2.1. Search Strategy

PubMed, Medline (via Ovid, 1946 to week 5 November 2024), Embase (via Ovid, 1974 to week 5 November 2024), Cochrane Central Register of Controlled Trials (CENTRAL) (via Cochrane Library issue 10/2024), and China National Knowledge Infrastructure (CNKI) were searched in week 5 November 2024. The reference lists of each study and the websites of vaccine manufacturers [25,26,27,28,29] were also searched to identify all relevant studies. Keywords used for the search included: (‘enterovirus’ or ‘EV’) AND (‘71’ or ‘A71’) AND (‘vaccin*’). For Chinese literature, keywords included: (‘腸病毒’ or ‘腸道病毒’) AND (‘71’ or ‘A71’) AND (‘疫苗’). Both traditional and simplified Chinese characters were used for searching.

### 2.2. Eligibility Criteria

Randomized controlled trials (RCTs), conducted in children and adolescents aged ≤18 years, comparing EV71 vaccine with placebo or with another EV71 vaccine were included. Exclusion criteria included: (1) non-RCT study; (2) conducted in animals, or adults; (3) studies on coadministration of vaccines (since coadministration might affect immunogenicity of vaccines); and (4) studies using 1 vaccine dose only, or booster doses (>2 doses). The first author conducted the literature search, screening and trial selection.

### 2.3. Data Extraction

The following data were extracted from the included studies by the first author: (1) first author and year of publication; (2) phase of trial; (3) location of study; (4) age of study population; (5) sample size; (6) vaccine dosage and its interval between 1st and 2nd dose; and (7) outcomes assessed. The outcomes assessed included VE (%), immunogenicity (including seropositive rate, seroconversion rate, geometric mean titer (GMT), Geometric Mean Fold Increase (GMFI)), and rate of adverse events in both vaccine and placebo groups.

### 2.4. Risk of Bias Assessment

The Cochrane Risk of Bias 2 tool (RoB2) [30] was used by the first author to assess risk of bias in included studies.

### 2.5. Certainty of Evidence Assessment

The Grading of Recommendations Assessment, Development and Evaluation (GRADE) tool was used to judge the certainty of evidence.

### 2.6. Data Synthesis and Statistical Analysis

Meta-analysis was performed for each outcome in each group using IBM SPSS 29. Heterogeneity was assessed by Cochran’s Q and I2 statistic and interpreted according to Cochrane guidelines (0–40%: might not be important; 30–60%: moderate heterogeneity; 50–90%: substantial heterogeneity; 75–100%: considerable heterogeneity) [31]. A random effects model was adopted due to significant heterogeneity. The restricted maximum likelihood estimator was employed. Study results were weighted by the inverse of their variance. Subgroup analysis for children with a seronegative baseline was conducted. For studies that did not report an overall result for subgroups, subgroup results were combined before meta-analysis. For study results of 0% or 100%, a fixed continuity correction of 0.5 was applied. Sensitivity analysis was performed by the leave-one-out method for meta-analyses with ≥3 studies. For meta-analyses with more than one study requiring continuity correction, further sensitivity analysis was performed by excluding all studies requiring continuity correction. For meta-analyses with ≥10 studies, publication bias was evaluated by visual inspection of a funnel plot and Egger’s test.

## 3. Results

### 3.1. Literature Search

**Figure 1:**
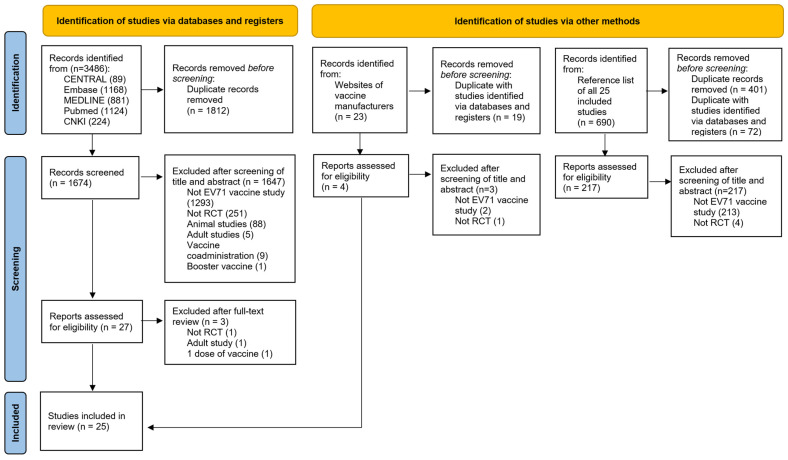
Literature search result.

Figure 1 shows that 3486 (3262 English and 224 Chinese), 23, and 690 articles were identified from electronic databases, websites of vaccine manufacturers, and reference lists of all included studies, respectively. A total of 2304 duplicates were removed, and 1867 were excluded after screening the title and abstract. Three studies were excluded after full-text review. A total of 25 studies were finally included in this systematic review (Table 2). The number of studies on Sinovac, CAMS, WIBP, Medigen and Enimmune vaccines was seven, four, six, three and one, respectively. Four studies were head-to-head trials between different vaccines.

### 3.2. Risk-of-Bias Assessment

Figure S1 showed the RoB2 risk-of-bias assessment result. Study 8 had high bias risk. Studies 6, 10, 16 and 17 had ‘some concerns’. The rest had low risk.

### 3.3. Certainty of Evidence Assessment

Study results were mostly of high certainty (Table S1).

### 3.4. Vaccine Efficacy

**Figure 2:**
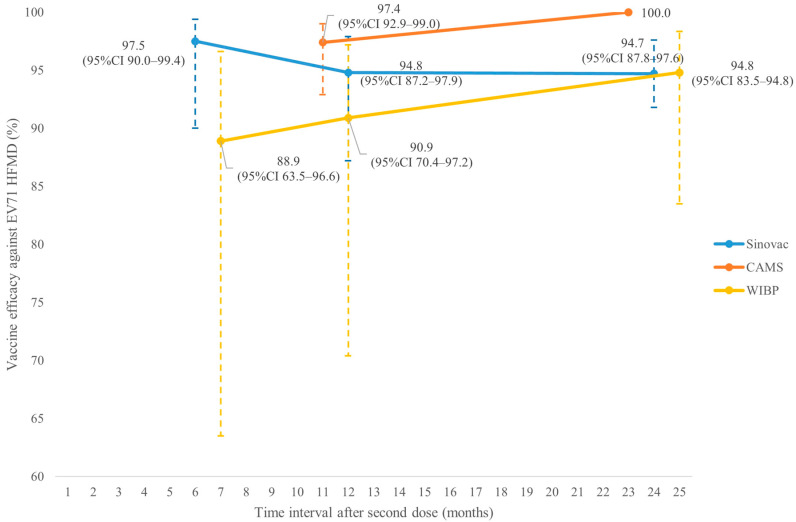
Vaccine efficacy of different vaccines against EV71 HFMD in children aged ≤5 years.

**Figure 3:**
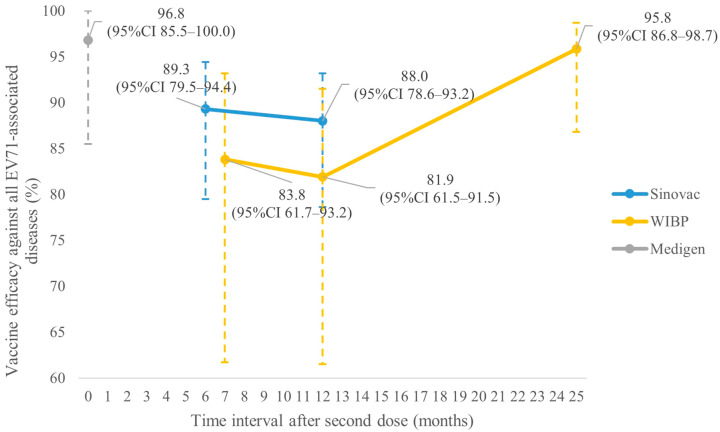
Vaccine efficacy of different vaccines against all EV71-associated diseases in children aged ≤5 years.

Figure 2 shows that VE (%) against EV71 HFMD in children aged ≤5 years of Sinovac at 6, 12 and 24 months after the second dose were 97.5% (95%CI 90.0–99.4), 94.8% (95%CI 87.2–97.9) and 94.7% (95%CI 87.8–97.6), respectively (studies 5 and 6). For WIBP, the corresponding results at 7, 12 and 25 months were 88.9% (95%CI 63.5–96.6), 90.9% (95%CI 70.4–97.2) and 94.8% (95%CI 83.5–94.8), respectively (studies 14 and 16). The corresponding results for CAMS at 11 and 23 months were 97.4% (95%CI 92.9–99.0) and 100.0%, respectively (studies 9 and 10). Figure 3 shows that VE (%) against all EV71-associated diseases of Sinovac at 6 and 12 months after the second dose was 89.3% (95%CI 79.5–94.4) and 88.0% (95%CI 78.6–93.2), respectively (study 5). The corresponding results for WIBP at 7, 12 and 25 months were 83.8% (95%CI 61.7–93.2), 81.9% (95%CI 61.5–91.5) and 95.8% (95%CI 86.8–98.7), respectively (studies 14 and 16). For Medigen, the corresponding result at 14 days was 96.8% (95%CI 85.5–100.0, study 20). The VE against EV71 HFMD severe complications of Sinovac at 6 and 12 months after the second dose was 100% (95%CI 42.4–100) and 100% (95%CI 42.6–100), respectively (study 5). At 11 months, no severe case was reported in the vaccine group of CAMS (study 9). The VE against EV71 HFMD hospitalization of Sinovac at 6 and 12 months after the second dose was 100% (95%CI 83.7–100) and 100% (83.7–100), respectively (study 5). The corresponding results for WIBP at 7 months and Medigen at 7 days were 100% (95%CI 41.6–100) and 81% (95%CI −12.3–100), respectively (studies 14 and 20).

### 3.5. Immunogenicity

Neutralizing antibody titer (NAbT) after vaccination was measured by assays involving serial dilution of serum. NAbT ≥ 1:8 indicated seropositivity. The greater the dilution, the higher the NAbT (e.g., NAbT ≥ 1:16 is higher than NAbT ≥ 1:8). The higher the NAbT, the more likely the child is protected from infection.

#### 3.5.1. Seropositive Rate

**Figure 4:**
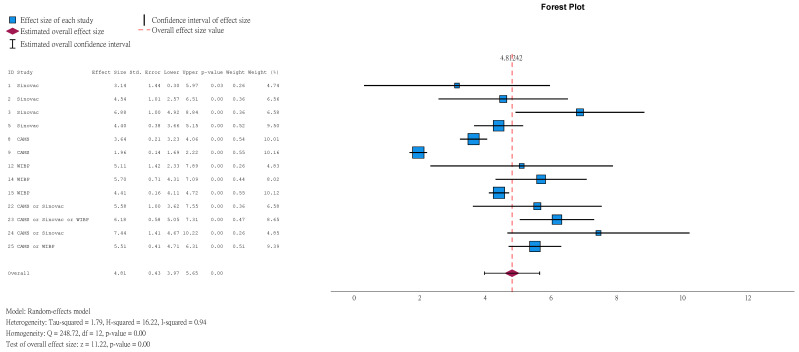
Natural logarithm of proportion of children aged ≤5 years in vaccine group with antibody titer ≥ 1:8 (1 month after 2nd dose).

Among children aged ≤5 years in the vaccine group, 99.19% (95%CI 98.15–99.65) achieved NAbT ≥ 1:8 at 1 month after the second dose (Figure 4), compared to 17.50% (95%CI 10.58–27.57) in the placebo group (Figure S2). Among those with a seronegative baseline (pre-vaccination NAbT < 1:8), the corresponding results were 99.30% (95%CI 96.99–99.84, Figure S3) and 7.95% (95%CI 3.41–17.44, Figure S4). At 1 month after the second dose, 98.52% (95%CI 96.40–99.40, Figure S5) and 10.92% (95%CI 2.01–42.25, Figure S6) of children aged ≤5 years in the vaccine and placebo groups achieved NAbT ≥ 1:16, respectively. Among those with a seronegative baseline, results were 99.25% (95%CI 96.12–99.86, Figure S7) and 2.08% (95%CI 1.26–3.42, Figure S8), respectively. Similarly, 97.01% (95%CI 93.00–98.76, Figure S9) and 8.96% (95%CI 3.62–20.52, Figure S10) of participants in the vaccine and placebo groups achieved NAbT ≥ 1:32, respectively. Among those with a seronegative baseline, results were 96.24% (95%CI 90.23–98.61, Figure S11) and 3.09 (95%CI 1.32–7.05, Figure S12).

#### 3.5.2. Seroconversion Rate

**Figure 5:**
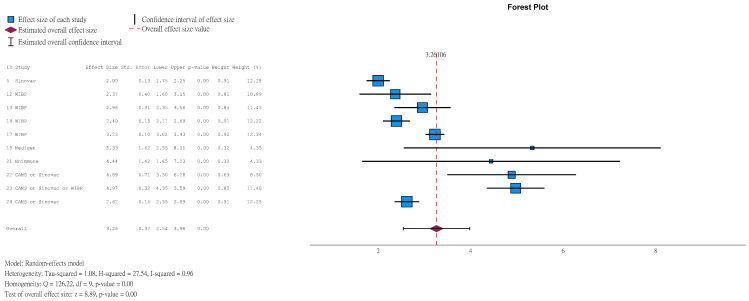
Natural logarithm of seroconversion rate of children aged ≤5 years in vaccine group (1 month after 2nd dose).

Seroconversion was defined as NAbT ≥ 1:8 for participants with a seronegative baseline, or at least a 4-fold rise in NT after vaccination for participants with baseline NAbT ≥ 1:8. Results showed that 96.30% (95%CI 92.71–98.17) of children aged ≤5 years in vaccine group achieved seroconversion at 1 month after the second dose (Figure 5), compared to 4.96% (95%CI 2.37–10.08, Figure S13) in the placebo group. Among those with a seronegative baseline, results were 98.93% (95%CI 96.85–99.64, Figure S14) and 3.63% (95%CI 1.83–7.08, Figure S15).

#### 3.5.3. Geometric Mean Titer (GMT)

**Figure 6:**
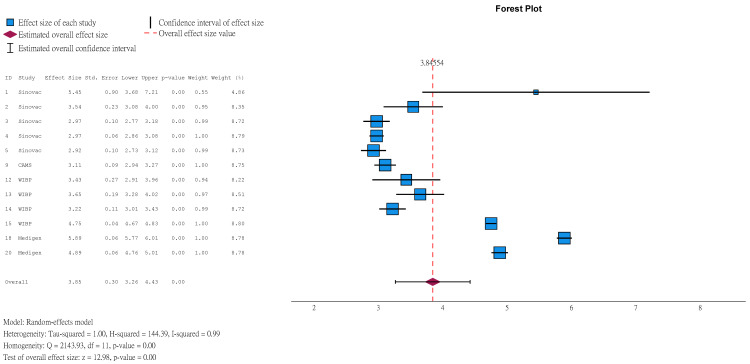
Natural logarithm of ratio of GMT between vaccine and placebo groups in children aged ≤5 years (1 month after 2nd dose).

GMT is a measure of NAbT. The greater the value, the higher the NAbT. In children aged ≤5 years, at 1 month after the second dose, the GMT in the vaccine group was 46.78 (95%CI 26.18–83.61) times that in the placebo group (Figure 6). Among those with a seronegative baseline, the ratio was higher at 60.99 (95%CI 28.58–130.17, Figure S16).

Older children had a higher GMT than younger children. Four studies compared GMT in children aged 6–11 months with those aged 1–4 years at 1 month after the second dose. The GMT of those aged 1–4 years was 76% higher than that of younger children (ratio = 1.76, 95%CI 1.12–2.76, Figure S17). Three studies compared the GMT in children aged 6–35 months with those aged 3–5 years. The GMT of those aged 3–5 years was 79% higher than that of younger children (ratio = 1.79, 95%CI 0.93–3.46, Figure S18), though the result was not statistically significant.

**Figure 7:**
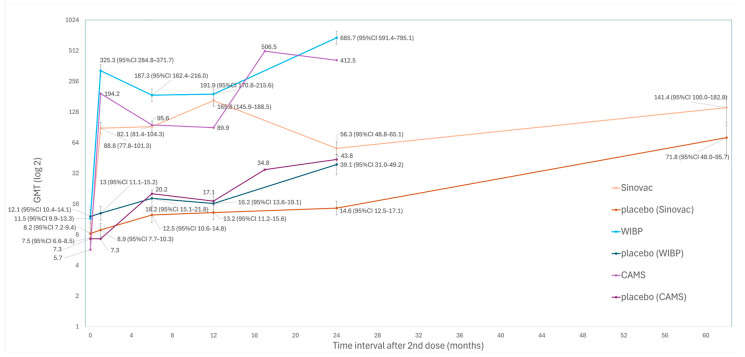
GMT trend of sub-genotype-C4 vaccines in children aged ≤5 years.

Figure 7 showed that only several studies (studies 5, 6, 7, 10, 14 & 16) examined longer-term GMT. While longer-term GMT remained high, all sub-genotype-C4 vaccine groups showed a transient decrease in GMT at some timepoints in the first 2 years. The GMT of Sinovac increased from 88.8 (95%CI 77.8–101.3) at 1 month to 165.8 (95%CI 145.9–188.5) at 1 year, followed by a decrease to 56.3 (95%CI 48.8–65.1) at 2 years, then an increase to 141.4 (95%CI 100.0–182.8). The GMT of WIBP peaked at 325.3 (95%CI 284.8–371.7) at 1 month, decreased to 187.3 (95%CI 162.4–216.0) at 6 months, then increased to 685.7 (95%CI 591.4–795.1) at 2 years. The GMT of CAMS peaked at 194.2 at 1 month, followed by a decrease to 89.9 at 12 months and an increase to 506.5 at 17 months.

An increasing trend of GMT was observed in all placebo groups. The GMT of Sinovac’s placebo group increased from 8.2 (95%CI 7.2–9.4) at day 0 to 14.6 (95%CI 12.5–17.1) at 2 years and 71.8 (95%CI 48.0–95.7) at 5 years. The GMT of WIBP’s placebo group increased from 12.1 (95%CI 10.4–14.1) at day 0 to 39.1 (95%CI 31.0–49.2) at 2 years. The corresponding results for CAMS were 7.3 and 412.5, respectively.

The only study (study 7) that studied GMT at 5 years showed that GMT at 5 years was higher than that at 2 years in both the vaccine and placebo groups.

**Figure 8:**
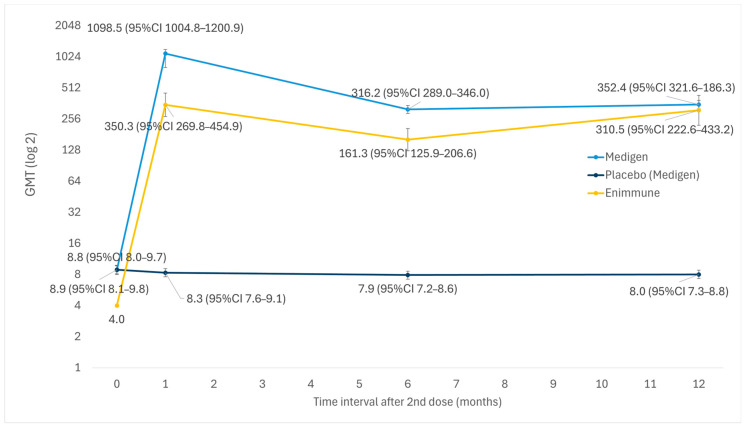
GMT trend of sub-genotype-B4 vaccines in children aged ≤5 years.

Medigen and Enimmune vaccines were based on sub-genotype B4. Figure 8 showed that vaccination led to a significant increase in GMT, which remained at high levels at 1 year after vaccination.

#### 3.5.4. Geometric Mean Fold Increase (GMFI)

GMFI represents the fold increase in GMT before and after vaccination. GMFI in the vaccine group at 1 month after the second dose in children aged ≤5 years was 28.41 (95%CI 22.18–36.39) times that of the placebo group (Figure S19).

### 3.6. Safety

**Figure 9:**
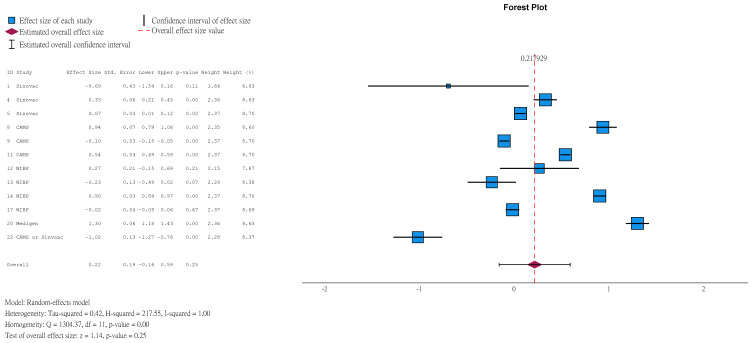
Natural logarithm of rate of any adverse events in children aged ≤5 years in vaccine group (1 month after 2nd dose).

At 1 month after the second dose, there were no statistically significant differences in the rate of any adverse events (AEs) (55.43% (95%CI 46.08–64.41) vs. 58.57% (95%CI 50.09–66.56), Figure 9, Figures S20 and S21) and unsolicited AEs (20.19% (95%CI 3.50–63.83) vs. 33.56% (95%CI 9.17–71.65), Figures S28–S30) between vaccine and placebo groups in children aged ≤5 years. The rate of serious AEs was lower in the vaccine group than the placebo group (1.23% (95%CI 0.58–2.69) vs. 1.34% (95%CI 0.58–3.07), Figures S22–S24) in children aged ≤5 years at 1 month after the second dose. There were no significant differences in the rate of solicited AEs (50.15% (95%CI 46.32–53.98) vs. 47.80% (95%CI 41.66–54.00), Figures S25–S27), systemic AEs (45.69% (95%CI 43.28–48.11) vs. 44.08% (95%CI 42.58–45.59), Figures S31–S33), and local AEs (15.53% (95%CI 6.98–31.04) vs. 9.69 (95%CI 2.03–35.75), Figures S34–S36) between vaccine and placebo groups in children aged ≤5 years at 7 days after the second dose.

Three studies showed that AEs in the vaccine group were less frequent in older children than in younger children at 1 month after the second dose. Study 4 showed a lower incidence of AEs in children aged 3–5 years than in children aged 6–35 months (47.0% vs. 60.4%, *p* = 0.003). Study 11 showed that the incidence of AEs decreased with increasing age (age 36–71 months: 28.9%, 24–35 months: 30.73%, 12–23 months: 40.23%, 6–11 months: 42.34%, *p* < 0.001). In study 13, the incidence of AEs in children aged 12–36 months was lower than in those aged 6–11 months (30.8% vs. 57.5%, *p* < 0.001).

### 3.7. Sensitivity Analysis and Publication Bias

Sensitivity analyses by the leave-one-out method showed that no single study significantly altered pooled estimates (Figures S37–S74). The results after excluding all studies requiring continuity correction did not differ significantly from the main analysis (Figures S75–S80). Potential publication bias was identified for the seropositive rate with NAbT ≥ 1:8 at 1 month, seroconversion rate at 1 month, ratio of GMT at 1 month, and rate of serious AEs at 1 month. However, publication bias was not identified for the rate of any AE at 1 month, and the difference in the rate of serious AEs between the vaccine and placebo groups at 1 month (Figures S81–S86, Table S2).

## 4. Discussion

The results showed that EV71 vaccines provided adequate protection to children (including those with a seronegative baseline), as evidenced by high VE, seropositive rate, seroconversion rate, GMT ratio and GMFI ratio. Overall increasing trend of GMT in the 5-year follow-up period in both sub-genotype-C4 vaccine groups and placebo groups suggested that exposure to community-circulating virus might have boosted immunity. Persistence of immunity might not be the sole result of vaccination but a result of vaccination and exposure to the circulating virus. However, all sub-genotype-C4 vaccine groups showed a transient decrease in GMT at some timepoints in the first 2 years, suggesting the possibility of some degree of immunity weaning after vaccination. The GMT in older children was much higher than that of younger children. Possible reasons included: more mature immune system [35] and higher baseline antibody level [42]. The safety profile was satisfactory since there was no statistically significant difference in AEs between the vaccine and placebo.

For the three vaccines approved in the Chinese Mainland, two review articles, published in 2016 [51] and 2021 [52], respectively, described the same phase 3 trials’ VE results against EV71 HFMD at 1 year follow-up. For the immunogenicity and safety analysis, our study included several more studies in addition to the five studies included in the 2016 article. A meta-analysis was performed to provide a better overview of results. Our study also provided longer-term immunogenicity results up to 5 years post-vaccination. Immunogenicity and safety were not studied in the 2021 article. For the two vaccines approved in Taiwan, China, studies were not yet published in 2016, while the phase 3 trial of Medigen was not yet published in 2021.

### 4.1. Limitations of the Included Studies

There were several limitations of the studies included in the review. Firstly, it was difficult to determine NAbT that is ‘protective’, and there was no international standard [32,49]. Secondly, most studies were unable to assess the duration of protection and antibody weaning due to limited follow-up time [22,32,38,39,47]. Thirdly, if background disease incidence was high, measured NAbT and VE would be higher due to immunity from natural infection [17,33,36,37,43,46]. Fourthly, underreporting of disease in vaccine recipients due to milder disease might overestimate VE [46]. Fifthly, the sample size was insufficient to detect very rare adverse events [9]. Lastly, cellular immunity and cross-genotype protection was not investigated [8,22,49].

### 4.2. Limitations of Meta-Analysis

Studies provided a wide range of results for GMT, GMFI and rate of AEs. This could be due to different study locations, study population, background epidemiology, and study methodology (e.g., differences in assays used for measuring GMT and different methods of monitoring AE). Heterogeneity might also cause asymmetry in funnel plots and significant results in Egger’s test in the absence of publication bias. Despite significant heterogeneity, the overall estimate by meta-analysis was compatible with the results of individual studies, i.e., vaccines were able to elicit an immune response, and there was no significant difference in AEs between the vaccine group and the placebo group. Sensitivity analyses also showed that results are robust.

VE, seropositive rate, and seroconversion rate in the vaccine group were affected by heterogeneity to a smaller extent, since all included studies provided a narrow range of similar results.

### 4.3. Implications for Policy and Future Research

Cross-protection against other viral strains should be studied. Currently marketed vaccines were developed based on sub-genotype C4 or B4. The ability of the vaccines to elicit an immune response against other strains warrants further investigation. The vaccine would likely be more effective if cross-protection was demonstrated. Some studies showed that the VE of C4 vaccines against HFMD caused by Coxsackievirus-A16 and other enteroviruses was low (<18%) [36,39]. Neutralization studies on C4 vaccines showed good cross-reactivity against EV71 sub-genotypes B4, B5, C2 and C5 [53,54]. Studies on B4 vaccines showed good immune response against EV71 sub-genotypes B5, C4 and C5 [9,20]. Secondly, real-world vaccine effectiveness had to be assessed. For example, post-marketing studies in the Chinese Mainland showed that VE against EV71 HFMD in children was >80% [55,56,57,58]. Thirdly, post-marketing surveillance of vaccine safety, particularly for extremely rare AEs, should be conducted. These events were often undetected in clinical trials due to limited sample size. The incidence of AEs reported in the Chinese Mainland was low, and most were mild or moderate [59,60,61,62]. Finally, the change in epidemiology after vaccine introduction should be reviewed. After vaccine introduction, the incidence of EV71 HFMD and its severe complications decreased significantly in many parts of the Chinese Mainland. However, this was accompanied by an increase in HFMD caused by other viruses, such as Coxsackievirus, and non-EV71 enteroviruses [63,64,65,66,67]. Serotype replacement and its potential consequences should be considered in risk–benefit assessments.

Before considering including EV71 vaccines in national childhood immunization programs, local epidemiology should be considered. Since sub-genotype C4 was the predominant strain in the Chinese Mainland, and the three vaccines approved in the Chinese Mainland were based on C4, the VE observed was mainly against C4 [37,46]. Secondly, cost-effectiveness studies need to be conducted. One study published in 2017 in the Chinese Mainland showed that routine pediatric EV71 vaccination would be cost-effective if the cost (including procurement, logistics and administration) was below USD$14.6/dose [68]. Another study in 2017 demonstrated cost-effectiveness at USD$9.2/dose [69]. A local economic evaluation would be necessary to assess whether inclusion as a national program is cost-effective. Thirdly, parents’ acceptance had to be assessed. Surveys can be conducted to explore the views and perceptions of parents or guardians. Lastly, practical issues, including availability of manpower, were important considerations for a successful immunization program. All currently approved vaccines are recommended to be stored at 2–8 °C. Some studies showed that coadministration of EV71 vaccine with other vaccines, including measles–mumps–rubella, influenza, hepatitis B, and meningococcal vaccines, was safe and did not interfere with immunogenicity [70,71,72]. Vaccine coadministration in the same visit would improve the uptake rate.

## 5. Conclusions

The results of the current meta-analysis support that EV71 vaccines are effective, immunogenic and safe. Areas with a high incidence of EV71 may consider introducing EV71 vaccines, taking into account the local epidemiology, economic analysis, parents’ acceptability and practical considerations of the immunization program. The latest development of EV71 vaccines should be closely monitored, including the multivalent vaccines (from bivalent to hexavalent) against HFMD, which cover different Coxsackievirus serotypes in addition to EV71 [73].

## Figures and Tables

**Table 1:** Currently approved enterovirus 71 vaccines worldwide.

Manufacturer	Platform	Strain (Sub-Genotype)	Indication (Age Group)	Dosage, Ingredients	Route, No. of Doses, Interval Between Doses	Place of Approval (Year of Approval)
Chinese Academy of Medical Sciences (CAMS) [14,15,16]	Inactivated (human diploid cell)	FY-23K-B (C4)	6–71 months	0.5 mL, ≥3 EU + Al(OH)3	IM, 2 doses, 28 days	Chinese Mainland (2015)
Sinovac [16,17,18]	Inactivated (vero cell)	H07 (C4)	6–71 months	0.5 mL, ≥3 EU + Al(OH)3	IM, 2 doses, 28 days	Chinese Mainland (2016)
Wuhan Institute of Biological Products * (WIBP) [15,19]	Inactivated (vero cell)	FY7VP5/AH/CHN/2008 (C4)	6–71 months	0.5 mL, ≥3 EU + Al(OH)3	IM, 2 doses, 28 days	Chinese Mainland (2016)
Medigen Vaccine Biologics [20,21]	Inactivated (vero cell)	E59 (B4)	2–71 months	0.5 mL, 2.5 μg EV71 viral antigen + aluminium phosphate	IM, 2 doses, 56 days	Taiwan, China (2023)
Enimmune # [22,23]	Inactivated (vero cell)	E59 (B4)	2–71 months	0.5 mL, ≥1.5 EV71 viral antigen unit + Al(OH)3	IM, 2 doses, 28 days	Taiwan, China (2023)

Abbreviations: EU = efficacy unit of neutralizing antibody titer against EV71, Al(OH)3 = aluminium hydroxide. * This vaccine was previously produced by Beijing Vigoo Biological. # Manufactured by Adimmune.

**Table 2:** Key results of studies included in the systematic review.

No.	Author, Year, Trial Phase, Place	Participants’ Age (Sample Size)	Dosage (Interval Between 1st & 2nd Dose)	Primary Outcome (Secondary Outcome)	Main Results
Sinovac					
1	Li et al., 2012, 1, Guangxi China [32]	6 m–11 y ^1^ (132)	100/200/400U (28d ^2^)	Safety (Immu)	Seropositive rate (56 days): vaccine >> placebo GMT (56 days): 400U > 200U > 100U >> placebo AE incidence: NSD between vaccine & placebo. Most were mild or moderate.
2	Li et al., 2014, 2, Guangxi China [33]	6–35 m (480)	100/200/400U (28d)	Immu (Safety)	Seropositive rates & GMT (56 days): 400U >200U >100U >> placebo From 8–14 months, seropositive rates in 200U & 400U groups decreased by 5–10%, but GMT remained unchanged AE incidence: NSD between vaccine & placebo
3	Hu et al., 2013, 3, Jiangsu China [34]	6–59 m (1400)	400U [3 lots] (28d)	Immu (Safety)	Seropositive rate & GMT (56 days): vaccine >> placebo Lot-to-lot consistency demonstrated AE incidence: NSD between vaccine & placebo
4	Gu et al., 2018, 3, Jiangsu China ^2^ [35]	6–59 m (1400)	400U (28d)	Immu (Safety)	At 56 days, vaccine elicited high seropositive rate & GMT GMT (56 days): children aged 3–5 years >> 6–35 months AE incidence: children aged 6–35 months >> 3–5 years
5	Zhu et al., 2014, 3, Jiangsu China [36]	6–35 m (10,077)	400U (28d)	VE (Immu, safety)	At 12 months, VE against EV71 HFMD, EV71 hospitalization, EV71 severe complications was 94.8%, 100% and 100%, respectively Seropositive rate & GMT (from 56 days–12 months): vaccine >> placebo GMT waned from 56 days to 12 months Neutralizing antibody titer ≥ 1:16 was associated with protection against EV71 HFMD AE incidence: NSD between vaccine & placebo
6	Li et al., 2016, 3, Jiangsu China ^3^ [37]	6–35 m (9803)	400U (28d)	VE (Immu, safety)	VE against EV71 HFMD (2 years) = 94.7% Seropositive rate & GMT (2 years): vaccine >> placebo AE incidence: NSD between vaccine & placebo
7	Hu et al., 2018, 3, Jiangsu China ^3^ [17]	6–35 m (343)	400U (28d)	Immu	At 5 years, seropositive rate & GMT in vaccine group remained stable and significantly higher than placebo group. Seropositive rate & GMT decreased with increasing age. Seropositive rate & GMT in placebo group rose significantly in the 5-year period
CAMS					
8	Zhang et al., 2015, unknown, Guangxi China [38]	6–72 m (900)	Unknown [3 lots] (28d)	Immu (Safety)	Seropositive rates & GMT (56 days): NSD between 3 vaccine lots Vaccine-related AEs & serious AEs: NSD between 3 vaccine lots
9	Li et al., 2014, 3, Guangxi China [39]	6–71 m (12,000)	100U (28d)	VE (Immu, safety)	VE against EV71 HFMD (12 months) = 97.4%. NSD between children aged 6–23 months and those aged 24–71 months. Seropositive rate & GMT (56 days–6 months): vaccine >> placebo GMT: waned from 56 days to 6 months. NSD between children aged 6–23 months and 24–71 months at 56 days or 6 months.
10	Liu et al., 2015, 3, Guangxi China ^4^ [40]	6–71 m (1100)	100U (28d)	VE (Immu)	Dropout rate > 60% No. of EV71 infections (2 years): placebo = 5, vaccine = 0 High GMT maintained at 2 years GMT in both vaccine and placebo groups increased from 6 months to 2 years GMT in placebo group (2 years): children aged 36–71 months > 24–35 months >> 12–23 months > 6–11 months
11	Zhang et al., 2016, 3, Guangxi China ^5^ [41]	6–71 m (12,000)	100U (28d)	Safety	AE incidence (28 days): children aged 6–11 months > 12–23 months > 24–35 months > 36–71 months (*p*-value for trend < 0.001)
WIBP ^6^					
12	Zhu et al., 2012, 1b, Jiangsu China [42]	6–60 m (360)	160/320/640U (28d)	Safety (Immu)	Seropositive rate & GMT (56 days): vaccine >> placebo AE incidence & severity: NSD between vaccine & placebo. Most were mild or moderate. No serious vaccine-related AEs were reported.
13	Zhu et al., 2013, 2, Jiangsu China [43]	6–36 m (1200)	160/320/640U (28d)	Immu (Safety)	Seroconversion rate & GMT (56 days): vaccine >> placebo Participants receiving adjuvant formulations had significantly higher GMT than those without GMT decreased at 8 months AE incidence: NSD between vaccine & placebo. Children aged 6–11 months >> aged 12–36 months
14	Zhu et al., 2013, 3, Jiangsu & Beijing China [44]	6–35 m (10,245)	320U (28d)	VE (Immu, safety)	At 14 months, VE against EV71 HFMD, EV71 disease, severe EV71 were 90.0%, 80.4%, and 100%, respectively Seropositive rates & GMT (56 days, 8 months, 14 months): vaccine >> placebo From 56 days to 8 months, GMT waned significantly, then remained consistent for the next 6 months Incidence of all AEs & serious AEs: NSD between vaccine & placebo. Most were mild or moderate and resolved within 3 days.
15	Chen et al., 2014, 3, Jiangsu & Beijing China ^7^ [45]	6–35 m (10,245)	320U (28d)	Immu (Safety)	Seropositive rate & GMT (56 days): vaccine >> placebo Lot-to-lot consistency demonstrated AE incidence: NSD between 3 vaccine batches & placebo. Most were mild or moderate and resolved within 3 days.
16	Wei et al., 2017, 3, Jiangsu & Beijing China ^8^ [46]	6–35 m (10,100)	320U (28d)	VE (Immu, safety)	VE against EV71 HFMD (26 months) = 94.84% Seropositive rate & GMT (26 months): vaccine >> placebo GMT increased from 56 days to 26 months in both vaccine & placebo groups Serious AE incidence: NSD between vaccine & placebo
17	Chen et al., 2022, 4, Jiangsu China [47]	6–35 m (3000)	≥3EU [6 lots] (28d)	Immu (Safety)	GMT & seroconversion rates (56 days): NSD between 6 batches AE incidence: NSD between 6 batches. Most were mild. No grade 4 AE.
Medigen					
18	Huang et al., 2019, 2, Taiwan China [20]	2 m–11 y (365)	1.25/2.5/5 µg (28d or 56d ^9^)	Safety (Immu)	Seropositive rate & GMT (56 days–2 years): vaccine >> placebo Most AEs were mild, unrelated to vaccine, and subsided within 7 days
19	Chiu et al., 2024, 2, Taiwan China ^10^ [8]	2 m–6 y (227)	1.25/2.5/5 µg (28d or 56d ^9^)	Safety (Immu)	At 5 years, GMT remained high, and no long-term safety issues were reported
20	Nguyen et al., 2022, 3, Taiwan China & Vietnam [9]	2–71 m (3061)	2.5 µg (56d)	VE (Immu, safety)	VE against EV71 disease (70 days) = 96.8% Seropositive rate & GMT (85 days–1 year): vaccine >> placebo AE incidence: NSD between vaccine & placebo. Most solicited AEs were mild and self-limited.
Enimmune					
21	Hung et al., 2019, 2b, Taiwan China [22]	6–35 m (135)	0.5/1.0 µg (28d)	Immu (Safety)	From 56 days to 1 year, there was a significant rise in seropositive rate & GMT in all vaccine groups No major AEs were reported
Head-to-head					
22	Xu et al., 2020, 4, Shandong China [48]	6–35 m (300)	4 groups: 2 doses Sinovac [≥3EU] 2 doses CAMS [≥3EU] Sinovac → CAMS CAMS → Sinovac (30d)	Immu (Safety)	Seropositive rate (60 days): NSD between 4 groups GMT (60 days): CAMS → Sinovac group >> other groups AE incidence: NSD between groups. No serious AEs related to vaccination.
23	Li et al., 2021, 4, Heibei, Zhejiang & Yunnan China [49]	6–35 m (1500)	Sinovac [480U] CAMS [125U] WIBP [420U] (28–35d)	Immu (Safety)	At 56 days, all 3 vaccines elicited high seropositive rates and were mutually non-inferior in all 2-group comparisons AE incidence: NSD between 3 vaccines. No serious AEs in any groups.
24	Zhang et al., 2021, 3, Yunnan China [50]	6–71 m (900)	Sinovac [≥3EU] CAMS [≥3EU] (30d)	Immu (Safety)	At 60 days, seroconversion rate in older children (36–71 months) receiving Sinovac was higher than that of older children receiving CAMS and non-inferior to that of younger children (6–35 months) receiving Sinovac. GMT (60 days): older children receiving Sinovac >> younger children receiving Sinovac AE incidence: NSD between 3 vaccines. Most were mild or moderate and resolved in a few days.
25	Tong et al., 2022, 3, Hubei China [15]	6–71 m (1600)	WIBP [≥3EU] CAMS [≥3EU] (30d)	Immu (Safety)	At 60 days, seroconversion rate & GMT in older children (36–71 months) receiving WIBP were non-inferior to older children receiving CAMS and younger children (6–35 months) receiving WIBP Seropositive rate & GMT (12 months): older children receiving WIBP >> older children receiving CAMS Vaccine-related AE incidence: NSD between 3 groups

Abbreviations: d = days, m = months, y = years, EU = efficacy units, Immu = immunogenicity, NSD = no statistically significant difference, U = units, VE = vaccine efficacy, > = higher than, >> = statistically significantly higher than. ^1^: Also recruited 36 adults and administered 3rd dose in children; ^2^: Post hoc analysis of study 3; ^3^: Follow-up study of study 5; ^4^: Follow-up study of study 9; ^5^: Post hoc analysis of study 9; ^6^: Previously produced by Vigoo; ^7^: Post hoc analysis of study 14; ^8^: Follow-up study of study 14; ^9^: Also administered 3rd dose; ^10^: Follow-up study of study 18.

## Data Availability

Data is contained within the article or Supplementary Materials.

## Supplementary Materials

The following supporting information can be downloaded at: https://www.mdpi.com/article/10.3390/vaccines14030235/s1, File S1: PRISMA 2020 Checklist [24]; Figure S1: RoB2 risk-of-bias assessment. Visualization of risk-of-bias assessment was generated by online tool [74]; Figure S2: Natural logarithm of proportion of children aged ≤5 years in placebo group with antibody titre ≥1:8 (1 month after 2nd dose); Figure S3: Natural logarithm of proportion of children aged ≤5 years with seronegative baseline in vaccine group with antibody titre ≥1:8 (1 month after 2nd dose); Figure S4: Natural logarithm of proportion of children aged ≤5 years with seronegative baseline in placebo group with antibody titre ≥1:8 (1 month after 2nd dose); Figure S5: Natural logarithm of proportion of children aged ≤5 years in vaccine group with antibody titre ≥1:16 (1 month after 2nd dose); Figure S6: Natural logarithm of proportion of children aged ≤5 years in placebo group with antibody titre ≥1:16 (1 month after 2nd dose); Figure S7: Natural logarithm of proportion of children aged ≤5 years with seronegative baseline in vaccine group with antibody titre ≥1:16 (1 month after 2nd dose); Figure S8: Natural logarithm of proportion of children aged ≤5 years with seronegative baseline in placebo group with antibody titre ≥1:16 (1 month after 2nd dose); Figure S9: Natural logarithm of proportion of children aged ≤5 years in vaccine group with antibody titre ≥1:32 (1 month after 2nd dose); Figure S10: Natural logarithm of proportion of children aged ≤5 years in placebo group with antibody titre ≥1:32 (1 month after 2nd dose); Figure S11: Natural logarithm of proportion of children aged ≤5 years with seronegative baseline in vaccine group with antibody titre ≥1:32 (1 month after 2nd dose); Figure S12: Natural logarithm of proportion of children aged ≤5 years with seronegative baseline in placebo group with antibody titre ≥1:32 (1 month after 2nd dose); Figure S13: Natural logarithm of seroconversion rate of children aged ≤5 years in placebo group (1 month after 2nd dose); Figure S14: Natural logarithm of seroconversion rate of children aged ≤5 years with seronegative baseline in vaccine group (1 month after 2nd dose); Figure S15: Natural logarithm of seroconversion rate of children aged ≤5 years with seronegative baseline in placebo group (1 month after 2nd dose); Figure S16: Natural logarithm of ratio of GMT between vaccine and placebo groups in children aged ≤5 years with seronegative baseline (1 month after 2nd dose); Figure S17: Natural logarithm of ratio of GMT in children aged 6–11 months as compared to those aged 1–4 years (1 month after 2nd dose); Figure S18: Natural logarithm of ratio of GMT in children aged 6–35 months as compared to those aged 3–5 years (1 month after 2nd dose); Figure S19: Natural logarithm of ratio of GMFI between vaccine and placebo groups in children aged ≤5 years (1 month after 2nd dose); Figure S20: Natural logarithm of rate of any adverse events in children aged ≤5 years in placebo group (1 month after 2nd dose); Figure S21: Difference in rate of any adverse events in children aged ≤5 years between vaccine and placebo groups (1 month after 2nd dose); Figure S22: Natural logarithm of rate of serious adverse events in children aged ≤5 years in vaccine group (1 month after 2nd dose); Figure S23: Natural logarithm of rate of serious adverse events in children aged ≤5 years in placebo group (1 month after 2nd dose); Figure S24: Difference in rate of serious adverse events in children aged ≤5 years between vaccine and placebo groups (1 month after 2nd dose); Figure S25: Natural logarithm of rate of solicited adverse events in children aged ≤5 years in vaccine group (7 days after 2nd dose); Figure S26: Natural logarithm of rate of solicited adverse events in children aged ≤5 years in placebo group (7 days after 2nd dose); Figure S27: Difference in rate of solicited adverse events in children aged ≤5 years between vaccine and placebo groups (7 days after 2nd dose); Figure S28: Natural logarithm of rate of unsolicited adverse events in children aged ≤5 years in vaccine group (1 month after 2nd dose); Figure S29: Natural logarithm of rate of unsolicited adverse events in children aged ≤5 years in placebo group (1 month after 2nd dose); Figure S30: Difference in rate of unsolicited adverse events in children aged ≤5 years between vaccine and placebo groups (1 month after 2nd dose); Figure S31: Natural logarithm of rate of systemic adverse events in children aged ≤5 years in vaccine group (7 days after 2nd dose); Figure S32: Natural logarithm of rate of systemic adverse events in children aged ≤5 years in placebo group (7 days after 2nd dose); Figure S33: Difference in rate of systemic adverse events in children aged ≤5 years between vaccine and placebo groups (7 days after 2nd dose); Figure S34: Natural logarithm of rate of local adverse events in children aged ≤5 years in vaccine group (7 days after 2nd dose); Figure S35: Natural logarithm of rate of local adverse events in children aged ≤5 years in placebo group (7 days after 2nd dose); Figure S36: Difference in rate of local adverse events in children aged ≤5 years between vaccine and placebo groups (7 days after 2nd dose); Figure S37: Sensitivity analysis for natural logarithm of proportion of children aged ≤5 years in vaccine group with antibody titre ≥1:8 (1 month after 2nd dose); Figure S38: Sensitivity analysis for natural logarithm of proportion of children aged ≤5 years in placebo group with antibody titre ≥1:8 (1 month after 2nd dose); Figure S39: Sensitivity analysis for natural logarithm of proportion of children aged ≤5 years with seronegative baseline in vaccine group with antibody titre ≥1:8 (1 month after 2nd dose); Figure S40: Sensitivity analysis for natural logarithm of proportion of children aged ≤5 years with seronegative baseline in placebo group with antibody titre ≥1:8 (1 month after 2nd dose); Figure S41: Sensitivity analysis for natural logarithm of proportion of children aged ≤5 years in vaccine group with antibody titre ≥1:16 (1 month after 2nd dose); Figure S42: Sensitivity analysis for natural logarithm of proportion of children aged ≤5 years in placebo group with antibody titre ≥1:16 (1 month after 2nd dose); Figure S43: Sensitivity analysis for natural logarithm of proportion of children aged ≤5 years with seronegative baseline in vaccine group with antibody titre ≥1:16 (1 month after 2nd dose); Figure S44: Sensitivity analysis for natural logarithm of proportion of children aged ≤5 years in vaccine group with antibody titre ≥1:32 (1 month after 2nd dose); Figure S45: Sensitivity analysis for natural logarithm of proportion of children aged ≤5 years in placebo group with antibody titre ≥1:32 (1 month after 2nd dose); Figure S46: Sensitivity analysis for natural logarithm of proportion of children aged ≤5 years with seronegative baseline in vaccine group with antibody titre ≥1:32 (1 month after 2nd dose); Figure S47: Sensitivity analysis for natural logarithm of proportion of children aged ≤5 years with seronegative baseline in placebo group with antibody titre ≥1:32 (1 month after 2nd dose); Figure S48: Sensitivity analysis for natural logarithm of seroconversion rate of children aged ≤5 years in vaccine group (1 month after 2nd dose); Figure S49: Sensitivity analysis for natural logarithm of seroconversion rate of children aged ≤5 years in placebo group (1 month after 2nd dose); Figure S50: Sensitivity analysis for natural logarithm of seroconversion rate of children aged ≤5 years with seronegative baseline in vaccine group (1 month after 2nd dose); Figure S51: Sensitivity analysis for natural logarithm of seroconversion rate of children aged ≤5 years with seronegative baseline in placebo group (1 month after 2nd dose); Figure S52: Sensitivity analysis for natural logarithm of ratio of GMT between vaccine and placebo groups in children aged ≤5 years (1 month after 2nd dose); Figure S53: Sensitivity analysis for natural logarithm of ratio of GMT between vaccine and placebo groups in children aged ≤5 years with seronegative baseline (1 month after 2nd dose); Figure S54: Sensitivity analysis for natural logarithm of ratio of GMT in children aged 6–11 months as compared to those aged 1–4 years (1 month after 2nd dose); Figure S55: Sensitivity analysis for natural logarithm of ratio of GMT in children aged 6–35 months as compared to those aged 3–5 years (1 month after 2nd dose); Figure S56: Sensitivity analysis for natural logarithm of ratio of GMFI between vaccine and placebo groups in children aged ≤5 years (1 month after 2nd dose); Figure S57: Sensitivity analysis for natural logarithm of rate of any adverse events in children aged ≤5 years in vaccine group (1 month after 2nd dose); Figure S58: Sensitivity analysis for natural logarithm of rate of any adverse events in children aged ≤5 years in placebo group (1 month after 2nd dose); Figure S59: Sensitivity analysis for difference in rate of any adverse events in children aged ≤5 years between vaccine and placebo groups (1 month after 2nd dose); Figure S60: Sensitivity analysis for natural logarithm of rate of serious adverse events in children aged ≤5 years in vaccine group (1 month after 2nd dose); Figure S61: Sensitivity analysis for natural logarithm of rate of serious adverse events in children aged ≤5 years in placebo group (1 month after 2nd dose); Figure S62: Sensitivity analysis for difference in rate of serious adverse events in children aged ≤5 years between vaccine and placebo groups (1 month after 2nd dose); Figure S63: Sensitivity analysis for natural logarithm of rate of solicited adverse events in children aged ≤5 years in vaccine group (7 days after 2nd dose); Figure S64: Sensitivity analysis for natural logarithm of rate of solicited adverse events in children aged ≤5 years in placebo group (7 days after 2nd dose); Figure S65: Sensitivity analysis for difference in rate of solicited adverse events in children aged ≤5 years between vaccine and placebo groups (7 days after 2nd dose); Figure S66: Sensitivity analysis for natural logarithm of rate of unsolicited adverse events in children aged ≤5 years in vaccine group (1 month after 2nd dose); Figure S67: Sensitivity analysis for natural logarithm of rate of unsolicited adverse events in children aged ≤5 years in placebo group (1 month after 2nd dose); Figure S68: Sensitivity analysis for difference in rate of unsolicited adverse events in children aged ≤5 years between vaccine and placebo groups (1 month after 2nd dose); Figure S69: Sensitivity analysis for natural logarithm of rate of systemic adverse events in children aged ≤5 years in vaccine group (7 days after 2nd dose); Figure S70: Sensitivity analysis for natural logarithm of rate of systemic adverse events in children aged ≤5 years in placebo group (7 days after 2nd dose); Figure S71: Sensitivity analysis for difference in rate of systemic adverse events in children aged ≤5 years in between vaccine and placebo groups (7 days after 2nd dose); Figure S72: Sensitivity analysis for natural logarithm of rate of local adverse events in children aged ≤5 years in vaccine group (7 days after 2nd dose); Figure S73: Sensitivity analysis for natural logarithm of rate of local adverse events in children aged ≤5 years in placebo group (7 days after 2nd dose); Figure S74: Sensitivity analysis for difference in rate of local adverse events in children aged ≤5 years between vaccine and placebo groups (7 days after 2nd dose); Figure S75: Natural logarithm of seroconversion rate of children aged ≤5 years in vaccine group (1 month after 2nd dose) after excluding of all studies requiring continuity correction; Figure S76: Natural logarithm of seroconversion rate of children aged ≤5 years with seronegative baseline in vaccine group (1 month after 2nd dose) after excluding of all studies requiring continuity correction; Figure S77: Natural logarithm of proportion of children aged ≤5 years in vaccine group with antibody titre ≥1:8 (1 month after 2nd dose) after excluding of all studies requiring continuity correction; Figure S78: Natural logarithm of proportion of children aged ≤5 years with seronegative baseline in vaccine group with antibody titre ≥1:8 (1 month after 2nd dose) after excluding of all studies requiring continuity correction; Figure S79: Natural logarithm of proportion of children aged ≤5 years in vaccine group with antibody titre ≥1:32 (1 month after 2nd dose) after excluding of all studies requiring continuity correction; Figure S80: Natural logarithm of rate of serious adverse events in children aged ≤5 years in placebo group (1 month after 2nd dose) after excluding of all studies requiring continuity correction; Figure S81: Funnel plot for natural logarithm of proportion of children aged ≤5 years in vaccine group with antibody titre ≥1:8 (1 month after 2nd dose); Figure S82: Funnel plot for natural logarithm of seroconversion rate of children aged ≤5 years in vaccine group (1 month after 2nd dose); Figure S83: Funnel plot for natural logarithm of ratio of GMT between vaccine and placebo groups in children aged ≤5 years (1 month after 2nd dose); Figure S84: Funnel plot for natural logarithm of rate of any adverse events in children aged ≤5 years in vaccine group (1 month after 2nd dose); Figure S85: Funnel plot for natural logarithm of rate of serious adverse events in children aged ≤5 years in vaccine group (1 month after 2nd dose); Figure S86: Funnel plot of difference in rate of serious adverse events in children aged ≤5 years between vaccine and placebo groups (1 month after 2nd dose); Table S1: Certainty of evidence assessment; Table S2: Egger’s test result.

## Abbreviations

The following abbreviations are used in this manuscript:

AE	Adverse Events
CAMS	Chinese Academy of Medical Sciences
EV71	Enterovirus 71
GMFI	Geometric Mean Fold Increase
GMT	Geometric Mean Titer
HFMD	Hand, Foot and Mouth Disease
NAbT	Neutralizing Antibody Titer
RCT	Randomized Controlled Trial
VE	Vaccine Efficacy
WIBP	Wuhan Institute of Biological Products
