# Supplementary material for: Efficacy, Immunogenicity, and Safety of Enterovirus 71 Vaccines in Children: A Systematic Review and Meta-Analysis
Source: Vaccines (Basel). 2026 Mar 4;14(3):235. doi: 10.3390/vaccines14030235 (PMC13030738; doi:10.3390/vaccines14030235)
Supplement: Supplementary file 1 [file vaccines-14-00235-s001.zip › vaccines-4174519-supplementary materials.pdf]

Supplementary Materials

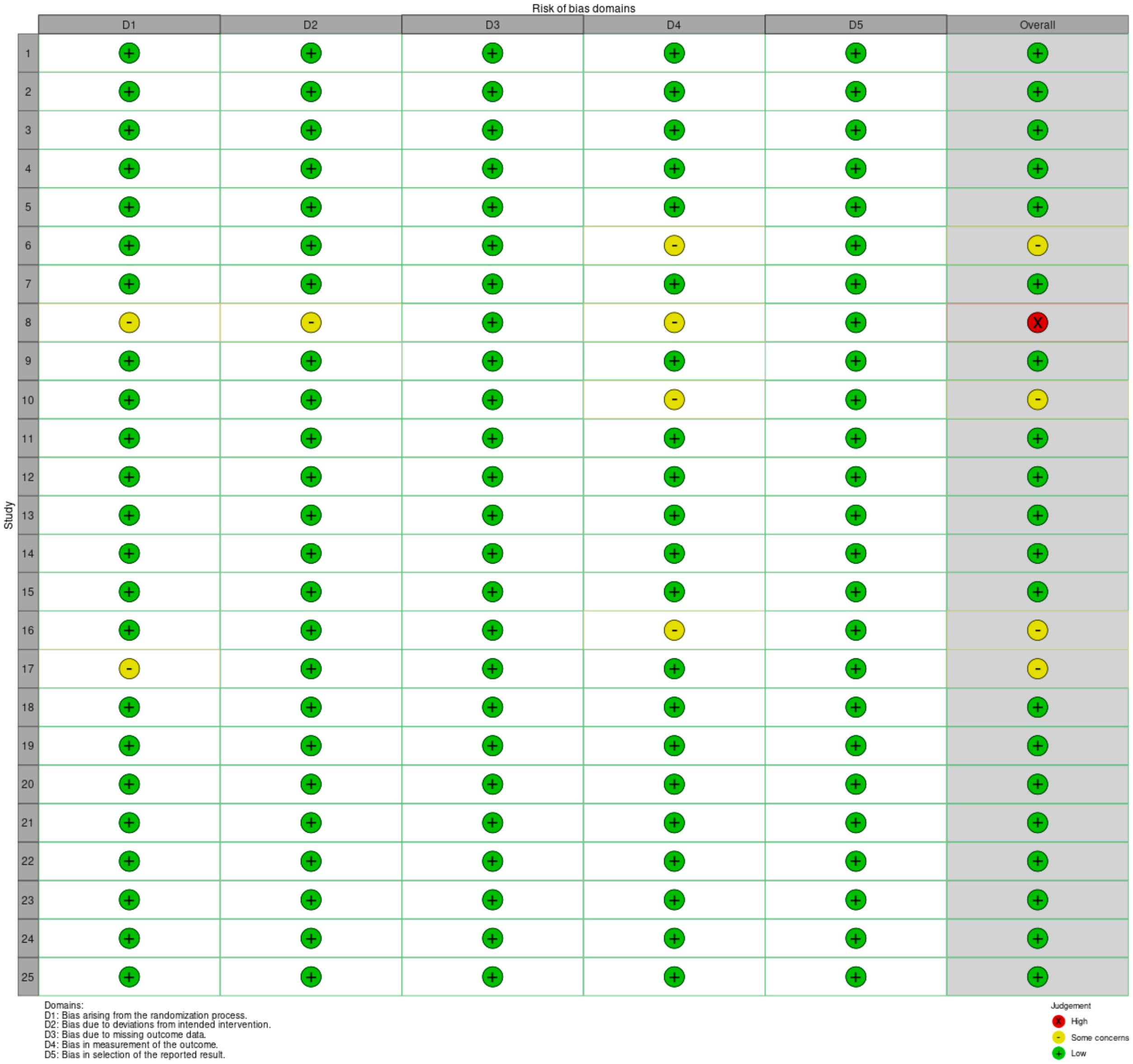

Figure S1. RoB2 risk-of-bias assessment.

Visualization of risk-of-bias assessment was generated by online tool [74].

Effect size of each study
Estimated overall effect size
Estimated overall confidence interval
Confidence interval of effect si
Overall effect size value

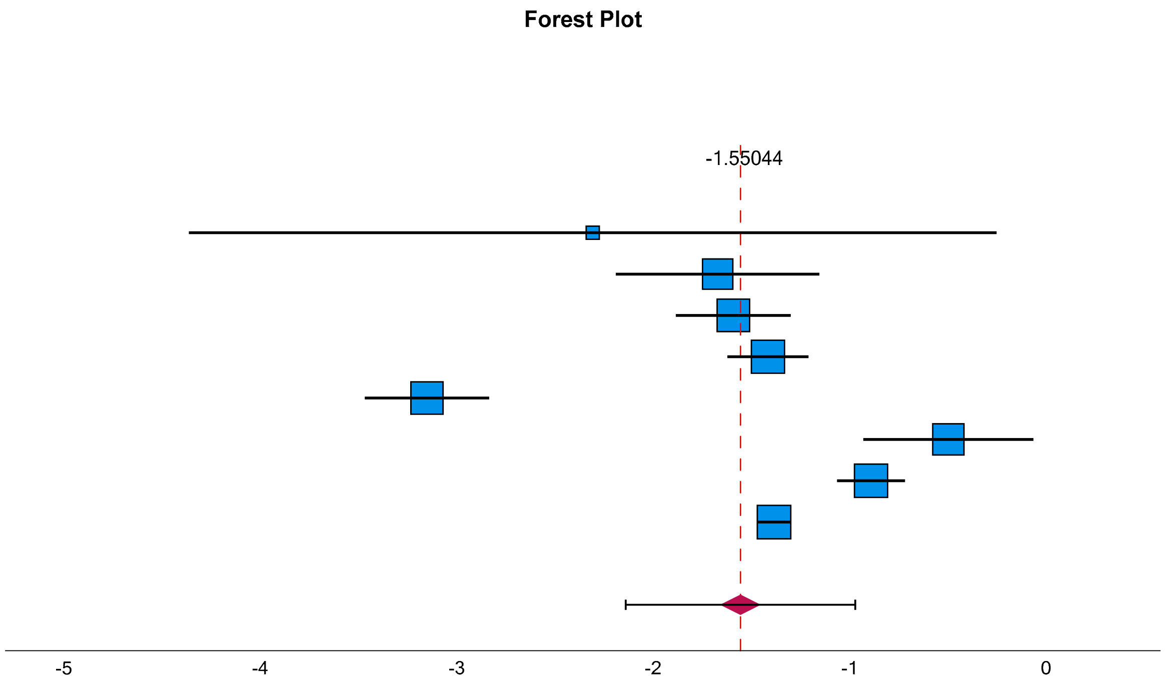

ID	Study	Effect Size	Std. Error	Lower	Upper	p-value	Weight	Weight (%)
1	Placebo	-2.30	1.05	-4.36	-0.25	0.03	0.58	5.14
2	Placebo	-1.67	0.26	-2.18	-1.15	0.00	1.43	12.70
3	Placebo	-1.59	0.15	-1.88	-1.29	0.00	1.53	13.63
5	Placebo	-1.41	0.11	-1.62	-1.20	0.00	1.56	13.87
9	Placebo	-3.15	0.16	-3.46	-2.83	0.00	1.52	13.55
12	Placebo	-0.49	0.22	-0.93	-0.06	0.03	1.47	13.10
14	Placebo	-0.89	0.09	-1.06	-0.71	0.00	1.57	13.94
15	Placebo	-1.38	0.04	-1.46	-1.30	0.00	1.58	14.07
Overall		-1.55	0.30	-2.13	-0.97	0.00		

Model: Random-effects model

Heterogeneity: Tau-squared = 0.63, H-squared = 47.29, I-squared = 0.98

Homogeneity: Q = 171.00, df = 7, p-value = 0.00

Test of overall effect size: z = -5.20, p-value = 0.00

Figure S2. Natural logarithm of proportion of children aged ≤5 years in placebo group with antibody titre ≥1:8 (1 month after 2nd dose).

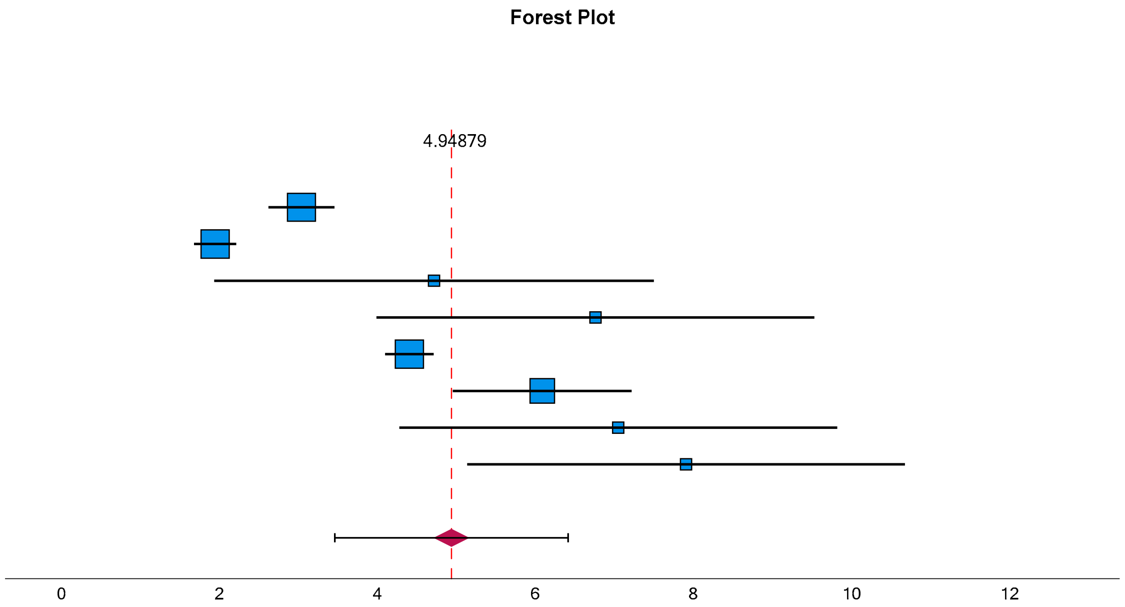

ID	Study	Effect Size	Std. Error	Lower	Upper	p-value	Weight	Weight (%)
8	CAMS	3.05	0.21	2.63	3.47	0.00	0.27	15.24
9	CAMS	1.96	0.14	1.69	2.22	0.00	0.27	15.35
12	WIBP	4.73	1.42	1.94	7.51	0.00	0.18	9.97
14	WIBP	6.77	1.42	4.00	9.55	0.00	0.18	9.99
15	WIBP	4.41	0.16	4.11	4.72	0.00	0.27	15.32
23	CAMS or Sinovac or WIBP	6.10	0.58	4.97	7.23	0.00	0.25	14.14
24	CAMS or Sinovac	7.06	1.41	4.29	9.83	0.00	0.18	10.00
25	CAMS or WIBP	7.92	1.41	5.15	10.69	0.00	0.18	10.00
	Overall	4.95	0.75	3.47	6.43	0.00		

Model: Random-effects model
Heterogeneity: Tau-squared = 3.69, H-squared = 42.92, I-squared = 0.98
Homogeneity: Q = 194.10, df = 7, p-value = 0.00
Test of overall effect size: z = 6.56, p-value = 0.00

Figure S3. Natural logarithm of proportion of children aged ≤5 years with seronegative baseline in vaccine group with antibody titre ≥1:8 (1 month after 2nd dose).

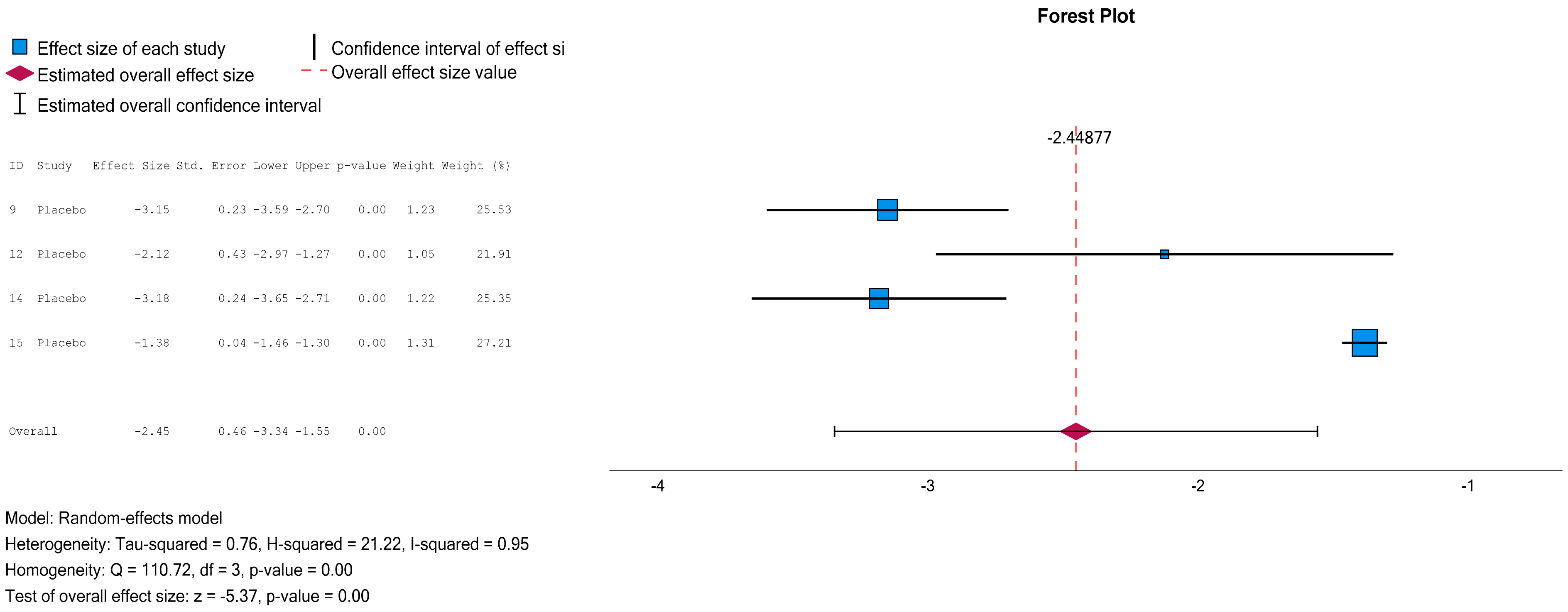

Figure S4. Natural logarithm of proportion of children aged ≤5 years with seronegative baseline in placebo group with antibody titre ≥1:8 (1 month after 2nd dose).

Effect size of each study
Estimated overall effect size
Estimated overall confidence interval
Confidence interval of effect si
Overall effect size value

ID	Study	Effect Size	Std. Error	Lower	Upper	p-value	Weight	Weight (%)
4	Sinovac	3.42	0.20	3.03	3.81	0.00	1.28	27.48
5	Sinovac	3.50	0.25	3.02	3.98	0.00	1.24	26.77
14	WIBP	5.29	0.58	4.16	6.43	0.00	0.93	19.97
25	CAMS or WIBP	4.90	0.30	4.31	5.49	0.00	1.20	25.78
Overall		4.20	0.46	3.29	5.11	0.00		

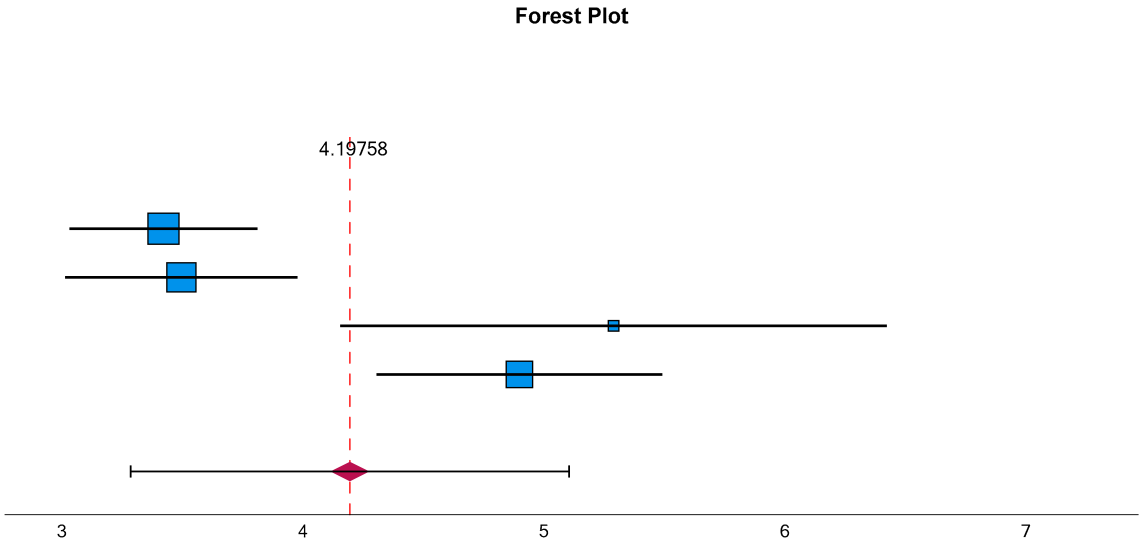

Model: Random-effects model
Heterogeneity: Tau-squared = 0.74, H-squared = 10.17, I-squared = 0.90
Homogeneity: Q = 24.87, df = 3, p-value = 0.00
Test of overall effect size: z = 9.04, p-value = 0.00

Figure S5. Natural logarithm of proportion of children aged ≤5 years in vaccine group with antibody titre ≥1:16 (1 month after 2nd dose).

Effect size of each study
Estimated overall effect size
Estimated overall confidence interval
Confidence interval of effect si
Overall effect size value

ID	Study	Effect Size	Std. Error	Lower	Upper	p-value	Weight	Weight (%)
4	Placebo	-3.99	0.45	-4.87	-3.10	0.00	0.38	31.65
5	Placebo	-1.49	0.11	-1.70	-1.28	0.00	0.41	34.15
14	Placebo	-0.96	0.09	-1.13	-0.78	0.00	0.41	34.20
Overall		-2.10	0.91	-3.89	-0.31	0.02		

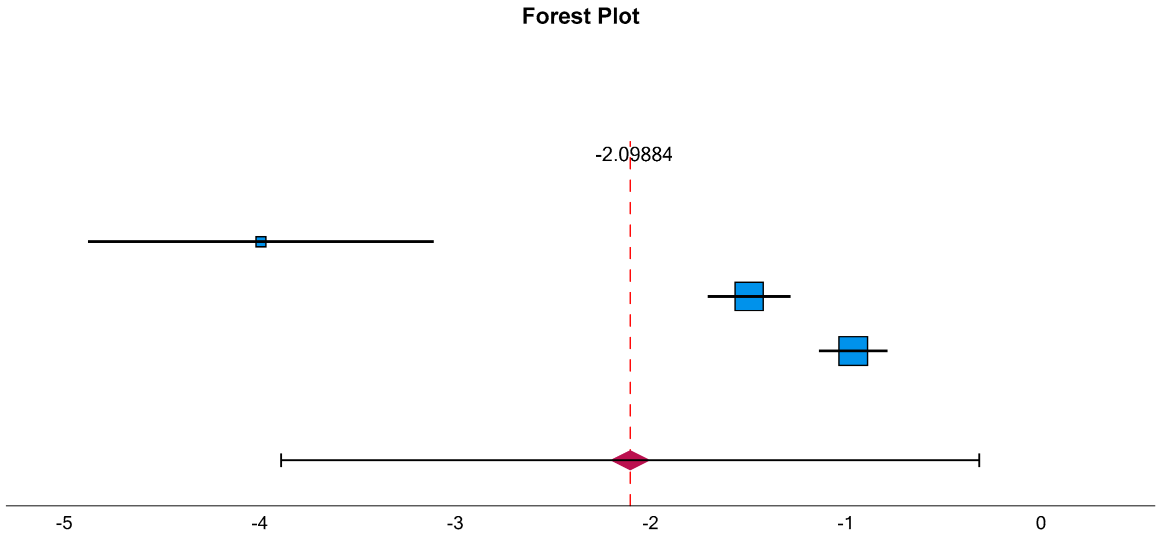

Model: Random-effects model
Heterogeneity: Tau-squared = 2.42, H-squared = 132.61, I-squared = 0.99
Homogeneity: Q = 52.40, df = 2, p-value = 0.00
Test of overall effect size: z = -2.30, p-value = 0.02

Figure S6. Natural logarithm of proportion of children aged ≤5 years in placebo group with antibody titre ≥1:16 (1 month after 2nd dose).

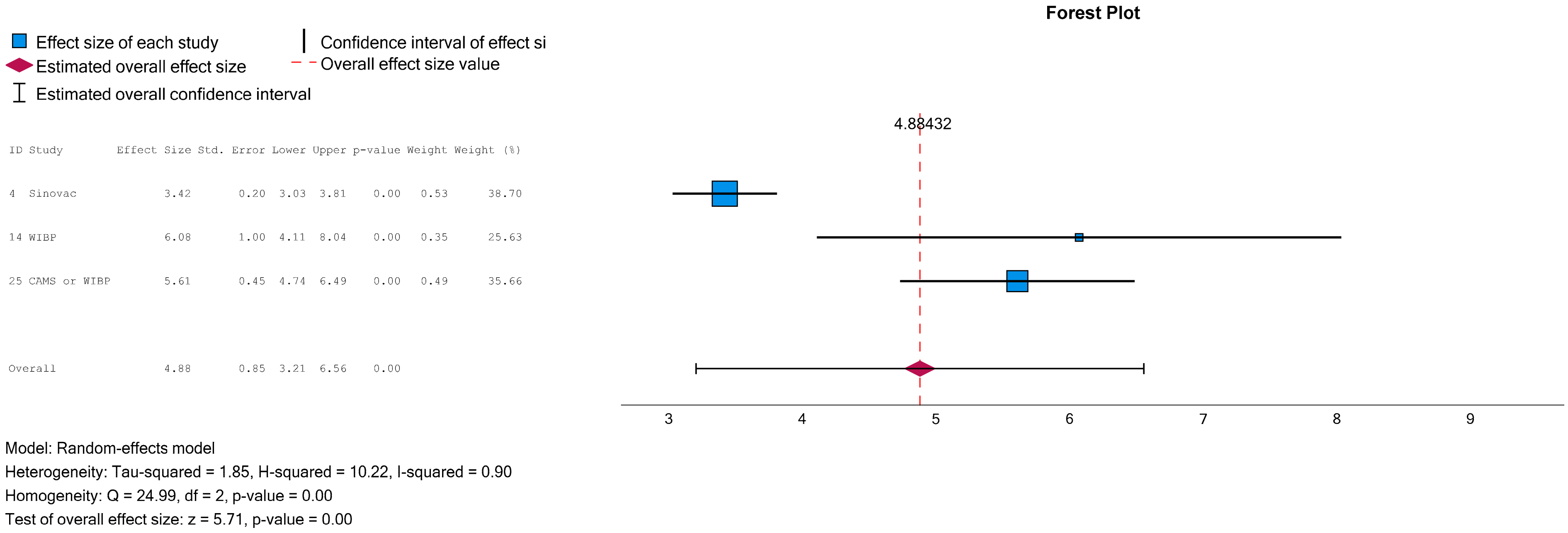

Figure S7. Natural logarithm of proportion of children aged ≤5 years with seronegative baseline in vaccine group with antibody titre ≥1:16 (1 month after 2nd dose).

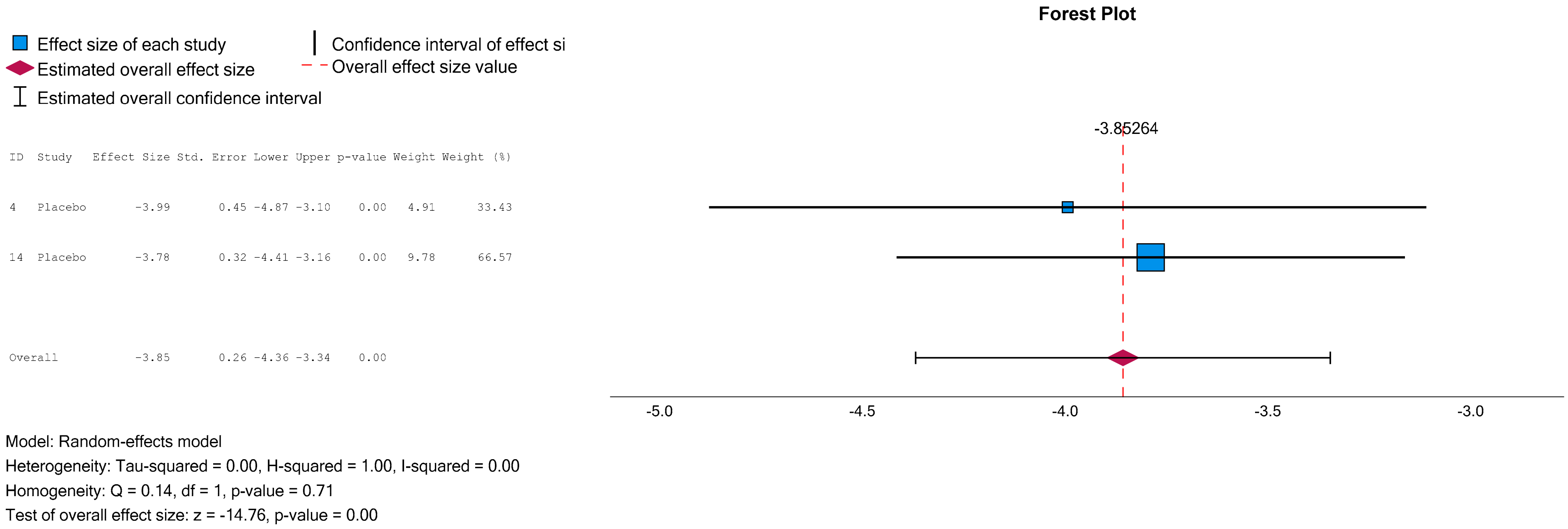

Figure S8. Natural logarithm of proportion of children aged ≤5 years with seronegative baseline in placebo group with antibody titre ≥1:16 (1 month after 2nd dose).

Effect size of each study
Estimated overall effect size
Estimated overall confidence interval
Confidence interval of effect si
Overall effect size value

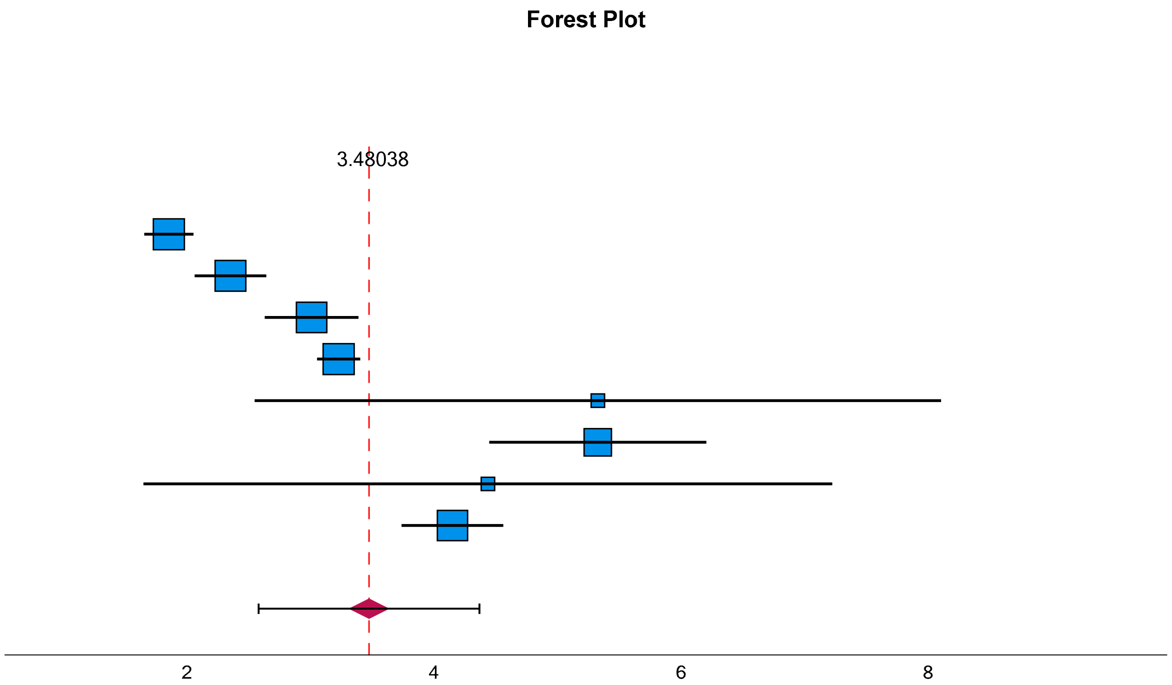

ID	Study	Effect Size	Std. Error	Lower	Upper	p-value	Weight	Weight (%)
4	Sinovac	1.86	0.10	1.66	2.06	0.00	0.72	15.05
5	Sinovac	2.36	0.15	2.07	2.65	0.00	0.72	14.92
14	WIBP	3.02	0.19	2.64	3.39	0.00	0.71	14.76
15	WIBP	3.23	0.09	3.06	3.41	0.00	0.72	15.07
18	Medigen	5.33	1.42	2.55	8.11	0.00	0.30	6.15
20	Medigen	5.33	0.45	4.45	6.21	0.00	0.64	13.23
21	Enimmune	4.44	1.42	1.65	7.23	0.00	0.29	6.13
25	WIBP	4.16	0.21	3.74	4.57	0.00	0.71	14.69
Overall		3.48	0.46	2.59	4.37	0.00		

Model: Random-effects model
Heterogeneity: Tau-squared = 1.37, H-squared = 46.99, I-squared = 0.98
Homogeneity: Q = 196.52, df = 7, p-value = 0.00
Test of overall effect size: z = 7.63, p-value = 0.00

Figure S9. Natural logarithm of proportion of children aged ≤5 years in vaccine group with antibody titre ≥1:32 (1 month after 2nd dose).

Effect size of each study
Estimated overall effect size
Estimated overall confidence interval
Confidence interval of effect si
Overall effect size value

ID	Study	Effect Size	Std. Error	Lower	Upper	p-value	Weight	Weight (%)
4	Placebo	-3.99	0.45	-4.87	-3.10	0.00	0.66	16.01
5	Placebo	-1.60	0.11	-1.82	-1.38	0.00	0.76	18.33
14	Placebo	-0.99	0.09	-1.17	-0.81	0.00	0.76	18.39
15	Placebo	-2.48	0.06	-2.61	-2.36	0.00	0.76	18.45
18	Placebo	-4.17	1.01	-6.15	-2.20	0.00	0.43	10.41
20	Placebo	-1.69	0.09	-1.86	-1.52	0.00	0.76	18.40
Overall		-2.32	0.49	-3.28	-1.35	0.00		

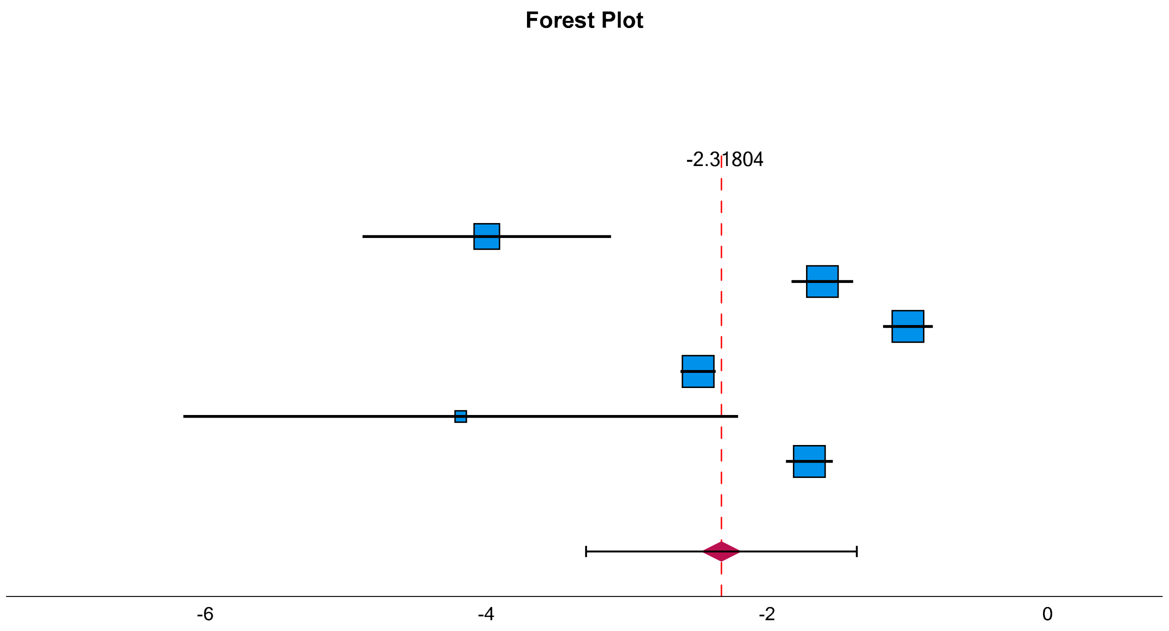

Model: Random-effects model

Heterogeneity: Tau-squared = 1.31, H-squared = 110.94, I-squared = 0.99

Homogeneity: Q = 224.39, df = 5, p-value = 0.00

Test of overall effect size: z = -4.71, p-value = 0.00

Figure S10. Natural logarithm of proportion of children aged ≤5 years in placebo group with antibody titre ≥1:32 (1 month after 2nd dose).

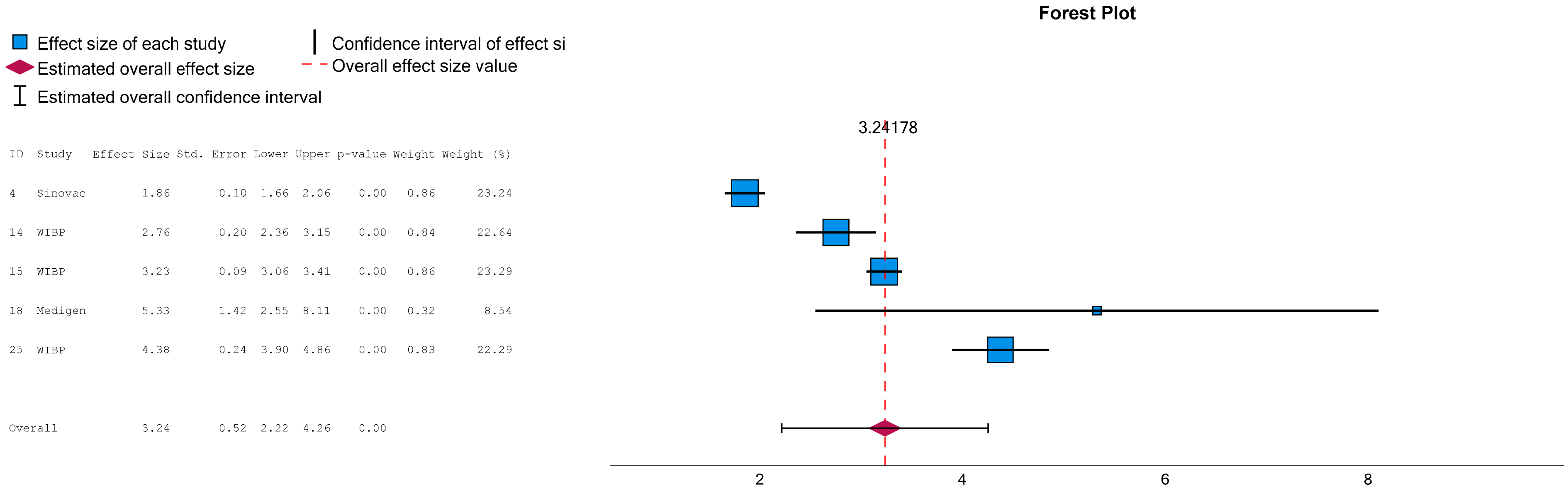

ID	Study	Effect Size	Std. Error	Lower	Upper	p-value	Weight	Weight (%)
4	Sinovac	1.86	0.10	1.66	2.06	0.00	0.86	23.24
14	WIBP	2.76	0.20	2.36	3.15	0.00	0.84	22.64
15	WIBP	3.23	0.09	3.06	3.41	0.00	0.86	23.29
18	Medigen	5.33	1.42	2.55	8.11	0.00	0.32	8.54
25	WIBP	4.38	0.24	3.90	4.86	0.00	0.83	22.29
Overall		3.24	0.52	2.22	4.26	0.00		

Model: Random-effects model

Heterogeneity: Tau-squared = 1.15, H-squared = 48.45, I-squared = 0.98

Homogeneity: Q = 153.31, df = 4, p-value = 0.00

Test of overall effect size: z = 6.24, p-value = 0.00

Figure S11. Natural logarithm of proportion of children aged ≤5 years with seronegative baseline in vaccine group with antibody titre ≥1:32 (1 month after 2nd dose).

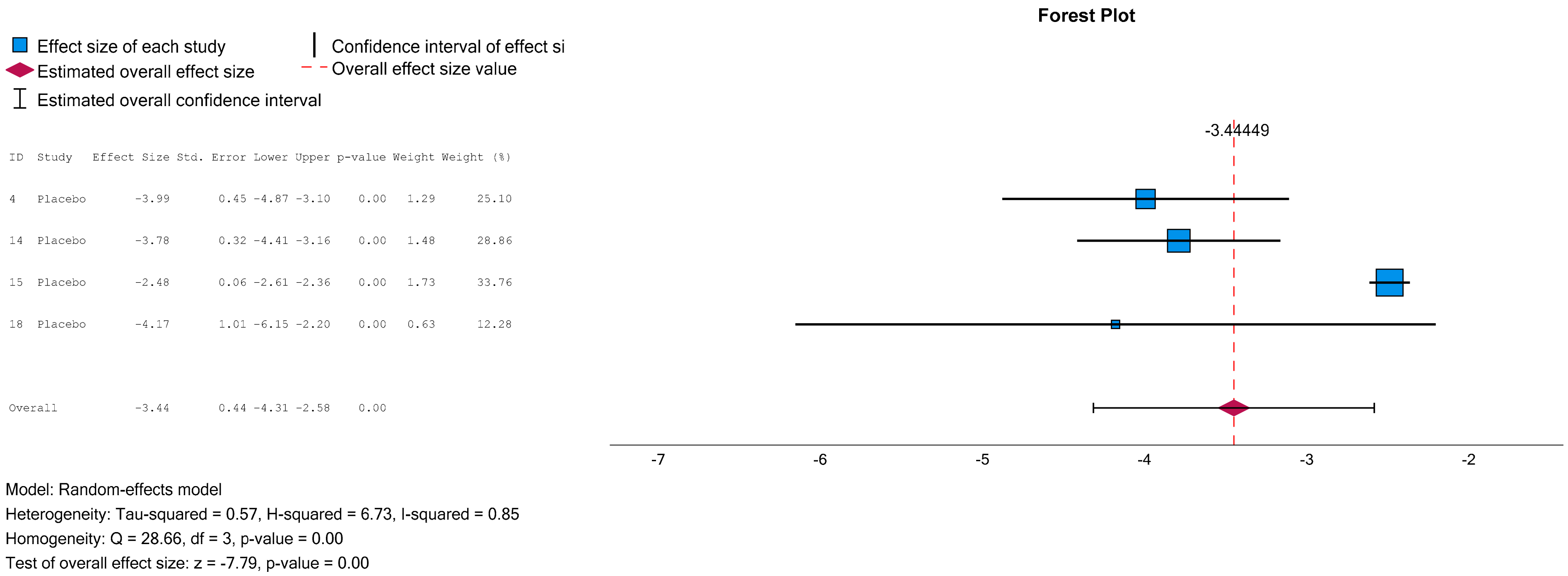

Figure S12. Natural logarithm of proportion of children aged ≤5 years with seronegative baseline in placebo group with antibody titre ≥1:32 (1 month after 2nd dose).

Effect size of each study
Estimated overall effect size
Estimated overall confidence interval
Confidence interval of effect si
Overall effect size value

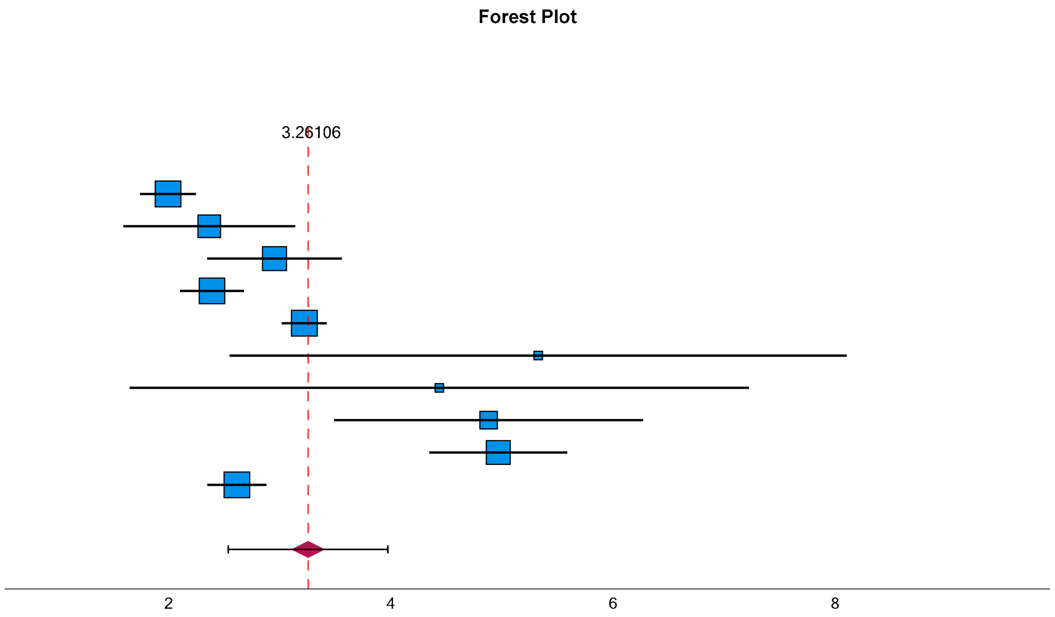

ID	Study	Effect Size	Std. Error	Lower	Upper	p-value	Weight	Weight (%)
5	Sinovac	2.00	0.13	1.75	2.25	0.00	0.91	12.28
12	WIBP	2.37	0.40	1.60	3.15	0.00	0.81	10.89
13	WIBP	2.96	0.31	2.35	3.56	0.00	0.85	11.45
14	WIBP	2.40	0.15	2.11	2.69	0.00	0.91	12.22
17	WIBP	3.23	0.10	3.02	3.43	0.00	0.92	12.34
18	Medigen	5.33	1.42	2.55	8.11	0.00	0.32	4.35
21	Enimmune	4.44	1.42	1.65	7.23	0.00	0.32	4.33
22	CAMS or Sinovac	4.89	0.71	3.50	6.28	0.00	0.63	8.50
23	CAMS or Sinovac or WIBP	4.97	0.32	4.35	5.59	0.00	0.85	11.40
24	CAMS or Sinovac	2.62	0.14	2.35	2.89	0.00	0.91	12.25
	Overall	3.26	0.37	2.54	3.98	0.00		

Model: Random-effects model
Heterogeneity: Tau-squared = 1.08, H-squared = 27.54, I-squared = 0.96
Homogeneity: Q = 126.22, df = 9, p-value = 0.00
Test of overall effect size: z = 8.89, p-value = 0.00

Figure S13. Natural logarithm of seroconversion rate of children aged ≤5 years in placebo group (1 month after 2nd dose).

Effect size of each study | Confidence interval of effect si
Estimated overall effect size | Overall effect size value
Estimated overall confidence interval

ID	Study	Effect Size	Std. Error	Lower	Upper	p-value	Weight	Weight (%)
12	WIBP	4.73	1.42	1.94	7.51	0.00	0.30	9.43
13	WIBP	4.32	0.71	2.92	5.71	0.00	0.54	17.08
14	WIBP	2.76	0.20	2.36	3.15	0.00	0.71	22.76
17	WIBP	4.76	0.24	4.28	5.23	0.00	0.71	22.46
18	Medigen	5.33	1.42	2.55	8.11	0.00	0.30	9.46
23	CAMS or Sinovac or WIBP	6.10	0.58	4.97	7.23	0.00	0.59	18.82
	Overall	4.53	0.56	3.42	5.64	0.00		

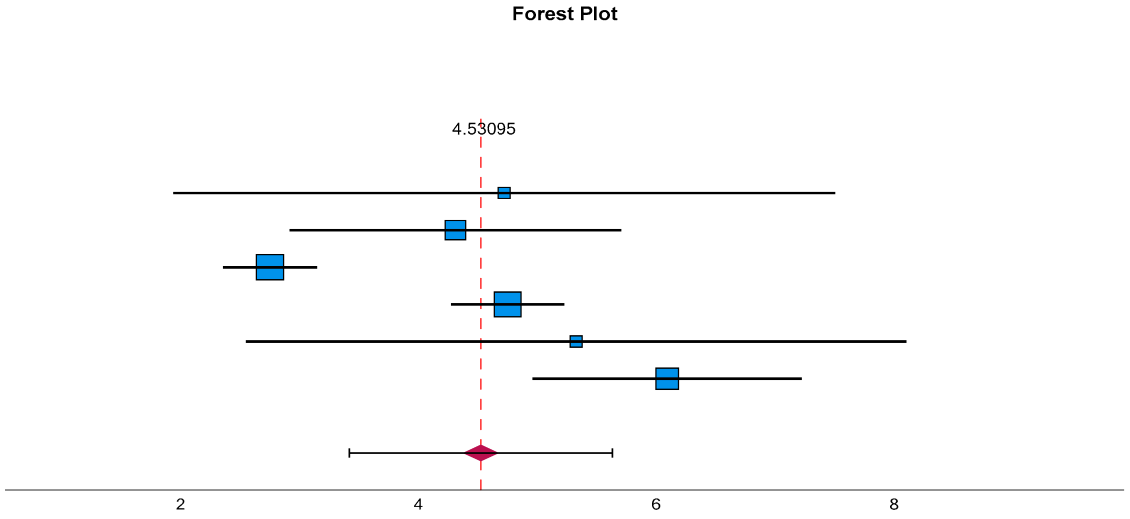

Model: Random-effects model
Heterogeneity: Tau-squared = 1.36, H-squared = 8.70, I-squared = 0.89
Homogeneity: Q = 59.90, df = 5, p-value = 0.00
Test of overall effect size: z = 8.03, p-value = 0.00

Figure S14. Natural logarithm of seroconversion rate of children aged ≤5 years with seronegative baseline in vaccine group (1 month after 2nd dose).

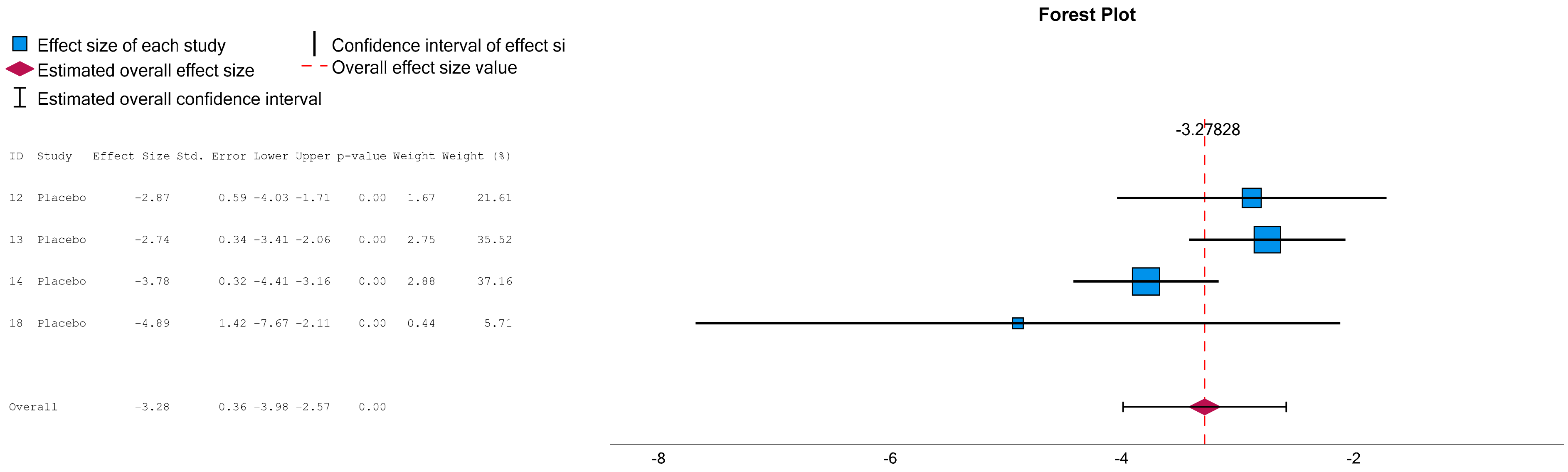

ID	Study	Effect Size	Std. Error	Lower	Upper	p-value	Weight	Weight (%)
12	Placebo	-2.87	0.59	-4.03	-1.71	0.00	1.67	21.61
13	Placebo	-2.74	0.34	-3.41	-2.06	0.00	2.75	35.52
14	Placebo	-3.78	0.32	-4.41	-3.16	0.00	2.88	37.16
18	Placebo	-4.89	1.42	-7.67	-2.11	0.00	0.44	5.71
Overall		-3.28	0.36	-3.98	-2.57	0.00		

Model: Random-effects model

Heterogeneity: Tau-squared = 0.24, H-squared = 2.10, I-squared = 0.52

Homogeneity: Q = 6.74, df = 3, p-value = 0.08

Test of overall effect size: z = -9.13, p-value = 0.00

Figure S15. Natural logarithm of seroconversion rate of children aged ≤5 years with seronegative baseline in placebo group (1 month after 2nd dose).

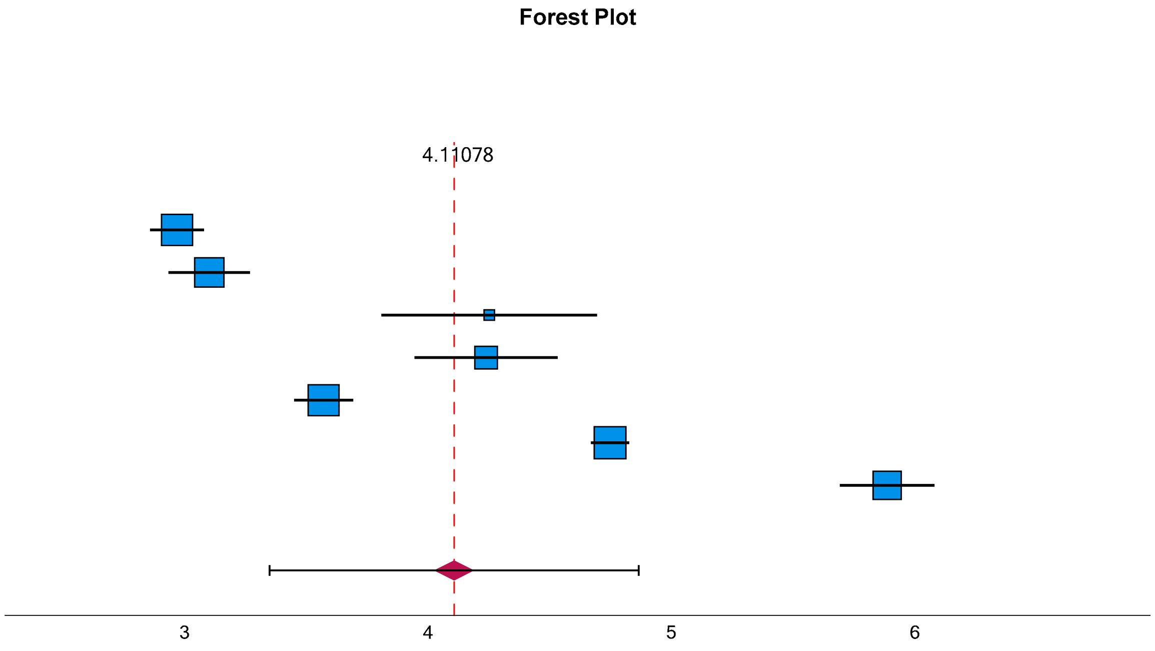

Effect size of each study
Estimated overall effect size
Estimated overall confidence interval
Confidence interval of effect si
Overall effect size value

ID	Study	Effect Size	Std. Error	Lower	Upper	p-value	Weight	Weight (%)
4	Sinovac	2.97	0.06	2.86	3.08	0.00	0.96	14.43
9	CAMS	3.11	0.09	2.94	3.27	0.00	0.96	14.38
12	WIBP	4.25	0.23	3.81	4.70	0.00	0.92	13.80
13	WIBP	4.24	0.15	3.95	4.54	0.00	0.95	14.17
14	WIBP	3.57	0.06	3.45	3.70	0.00	0.96	14.42
15	WIBP	4.75	0.04	4.67	4.83	0.00	0.97	14.46
18	Medigen	5.89	0.10	5.69	6.08	0.00	0.96	14.34
Overall		4.11	0.39	3.35	4.87	0.00		

Model: Random-effects model

Heterogeneity: Tau-squared = 1.03, H-squared = 190.90, I-squared = 0.99

Homogeneity: Q = 1188.66, df = 6, p-value = 0.00

Test of overall effect size: z = 10.63, p-value = 0.00

Figure S16. Natural logarithm of ratio of GMT between vaccine and placebo groups in children aged ≤5 years with seronegative baseline (1 month after 2nd dose).

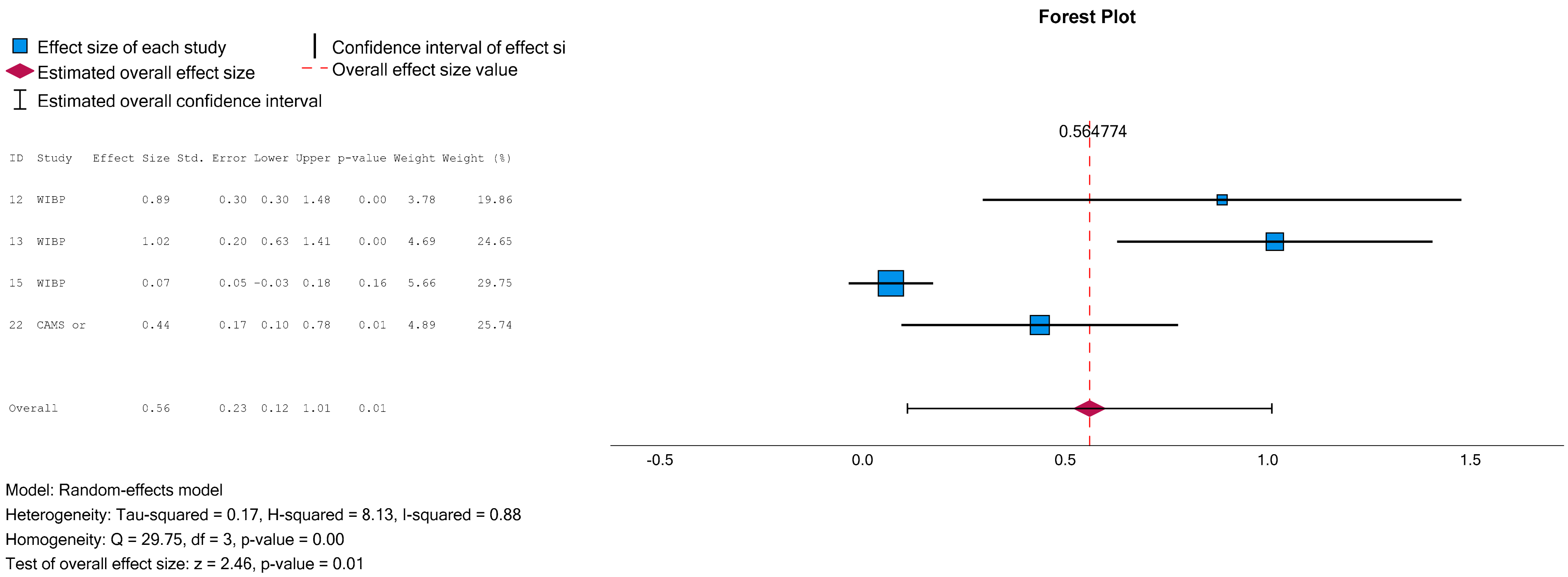

Figure S17. Natural logarithm of ratio of GMT in children aged 6-11 months as compared to those aged 1-4 years (1 month after 2nd dose).

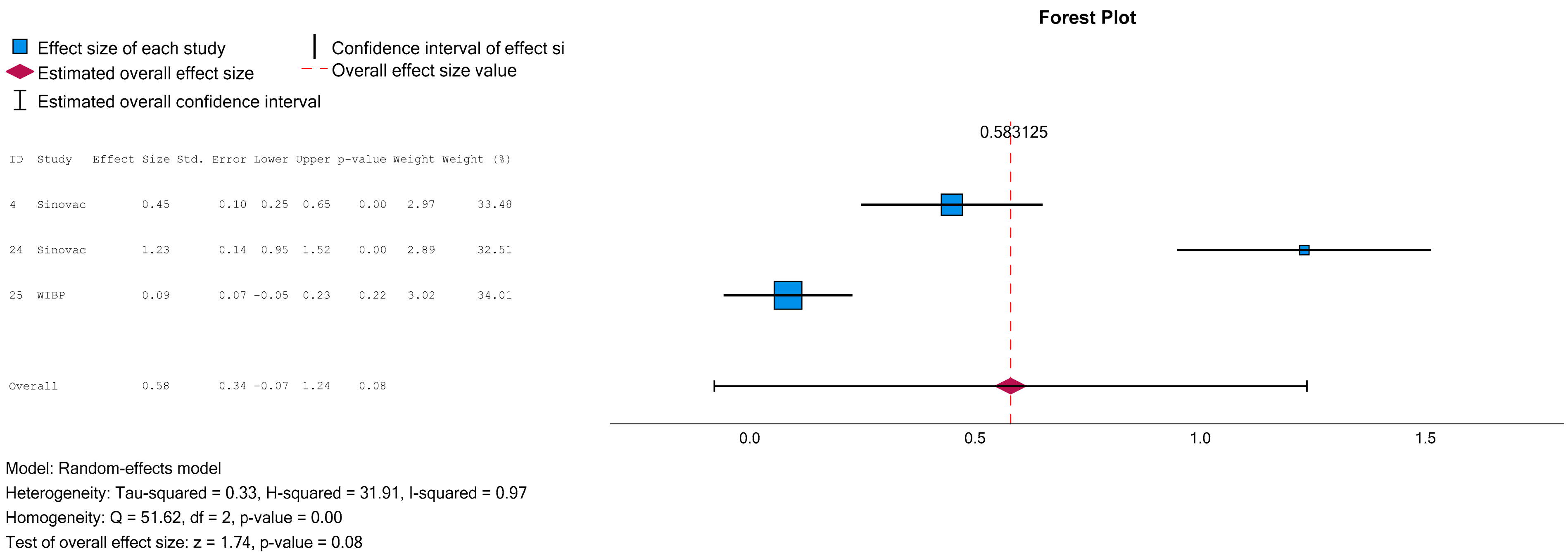

Figure S18. Natural logarithm of ratio of GMT in children aged 6-35 months as compared to those aged 3-5 years (1 month after 2nd dose).

- Effect size of each study
- Estimated overall effect size
- Estimated overall confidence interval
- Confidence interval of effect si
- Overall effect size value

ID	Study	Effect Size	Std. Error	Lower	Upper	p-value	Weight	Weight (%)
2	Sinovac	3.44	0.15	3.15	3.73	0.00	11.80	18.82
5	Sinovac	3.00	0.06	2.89	3.11	0.00	15.18	24.21
12	WIBP	3.53	0.24	3.07	4.00	0.00	8.40	13.40
13	WIBP	3.68	0.14	3.41	3.94	0.00	12.27	19.57
14	WIBP	3.25	0.06	3.13	3.37	0.00	15.06	24.01
Overall		3.35	0.13	3.10	3.59	0.00		

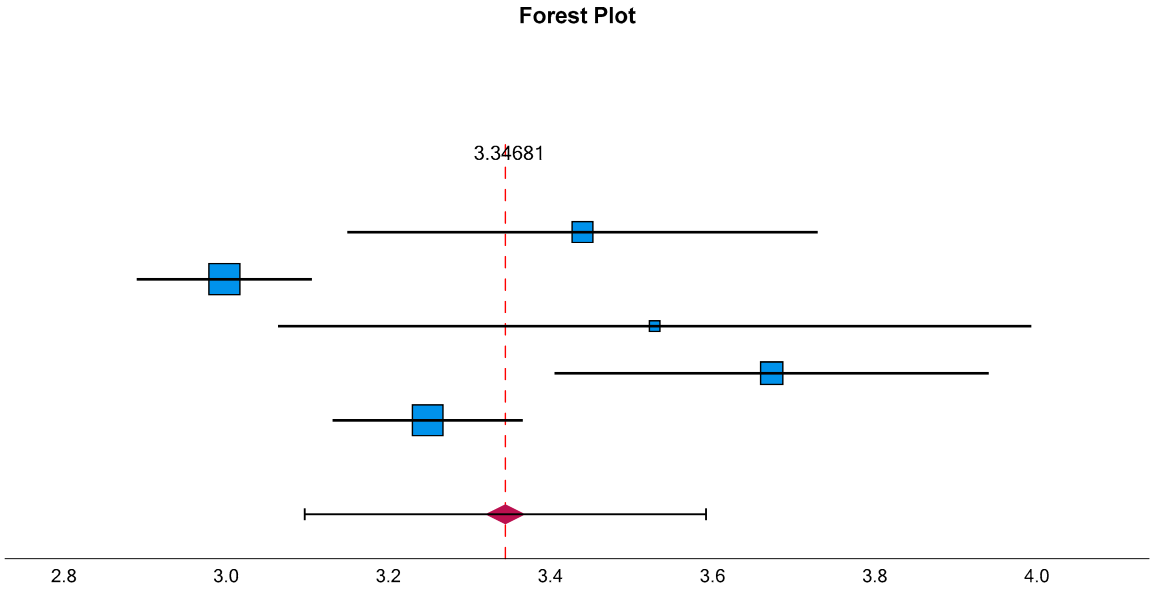

Model: Random-effects model
Heterogeneity: Tau-squared = 0.06, H-squared = 8.24, I-squared = 0.88
Homogeneity: Q = 30.47, df = 4, p-value = 0.00
Test of overall effect size: z = 26.50, p-value = 0.00

Figure S19. Natural logarithm of ratio of GMFI between vaccine and placebo groups in children aged ≤5 years (1 month after 2nd dose).

Effect size of each study
Estimated overall effect size
Estimated overall confidence interval
Confidence interval of effect si
Overall effect size value

ID	Study	Effect Size	Std. Error	Lower	Upper	p-value	Weight	Weight (%)
1	Placebo	-0.34	0.41	-1.15	0.48	0.42	2.35	7.18
4	Placebo	0.37	0.11	0.16	0.58	0.00	3.77	11.49
5	Placebo	0.11	0.03	0.06	0.17	0.00	3.93	11.99
9	Placebo	-0.01	0.03	-0.06	0.04	0.76	3.93	12.00
11	Placebo	0.40	0.03	0.35	0.45	0.00	3.93	12.00
12	Placebo	0.18	0.21	-0.24	0.59	0.40	3.35	10.22
13	Placebo	-0.15	0.13	-0.40	0.10	0.25	3.70	11.28
14	Placebo	0.86	0.03	0.80	0.92	0.00	3.93	11.99
20	Placebo	1.37	0.06	1.25	1.50	0.00	3.88	11.84
Overall		0.35	0.17	0.00	0.69	0.05		

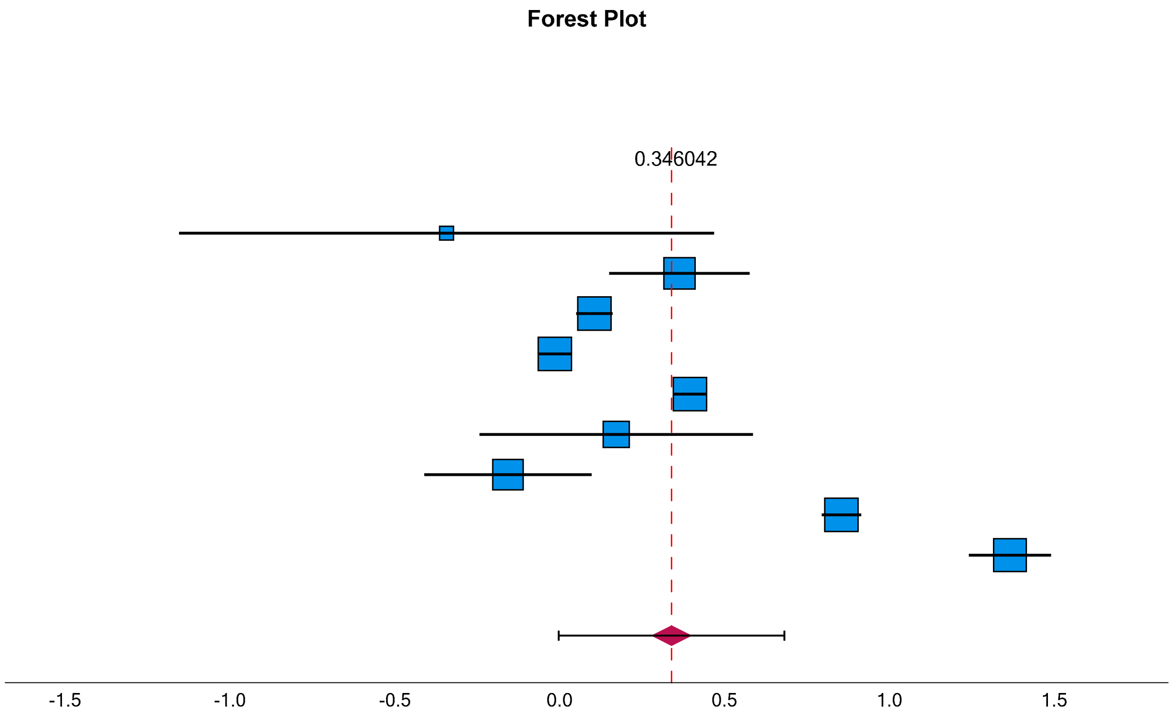

Model: Random-effects model
Heterogeneity: Tau-squared = 0.25, H-squared = 141.52, I-squared = 0.99
Homogeneity: Q = 822.95, df = 8, p-value = 0.00
Test of overall effect size: z = 1.98, p-value = 0.05

Figure S20. Natural logarithm of rate of any adverse events in children aged ≤5 years in placebo group (1 month after 2nd dose).

Effect size of each study | Confidence interval of effect size
Estimated overall effect size | Overall effect size value
No-effect value | Estimated overall confidence int

ID	Risk Difference	Std. Error	Lower	Upper	p-value	Weight	Weight (%)
1	-0.08	0.14	-0.36	0.19	0.55	50.63	0.42
4	-0.01	0.03	-0.07	0.05	0.75	778.21	6.45
5	-0.01	0.01	-0.03	0.01	0.27	2164.91	17.95
9	-0.02	0.01	-0.04	-0.01	0.01	2241.61	18.58
11	0.03	0.01	0.02	0.05	0.00	2264.16	18.77
12	0.02	0.07	-0.12	0.17	0.76	171.02	1.42
13	-0.02	0.05	-0.11	0.07	0.65	412.17	3.42
14	0.01	0.01	-0.01	0.03	0.32	2253.62	18.68
20	-0.01	0.01	-0.04	0.02	0.44	1727.30	14.32
Overall	-0.00	0.01	-0.02	0.02	0.87		

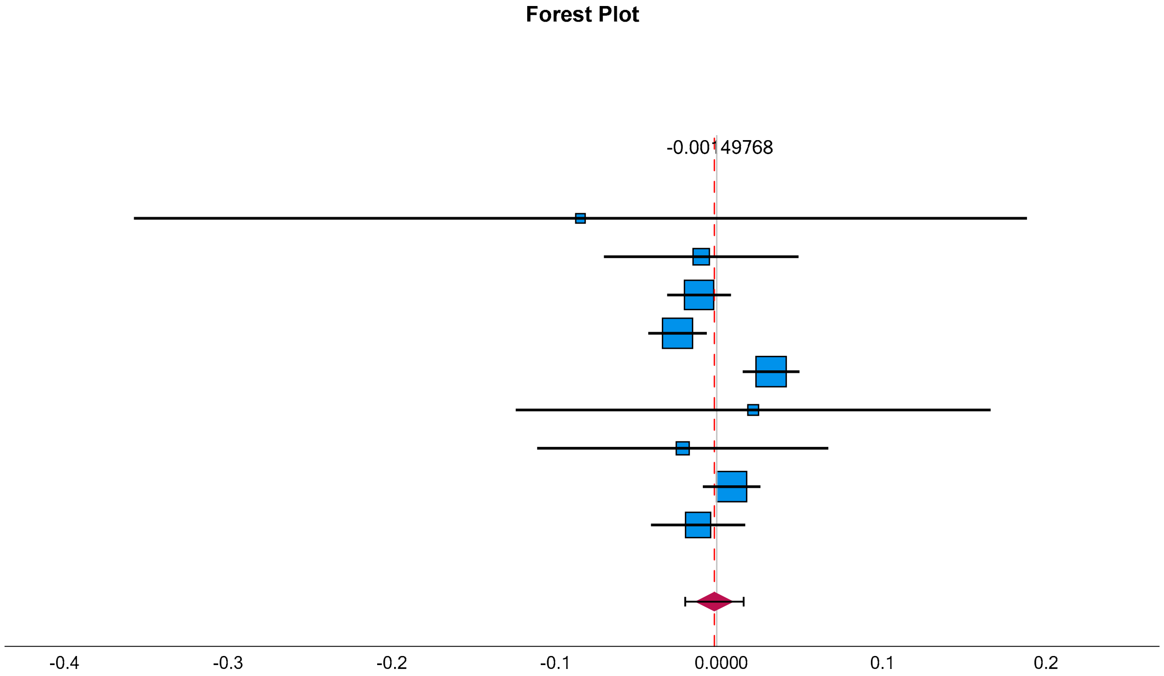

Model: Random-effects model
Heterogeneity: Tau-squared = 0.00, H-squared = 2.94, I-squared = 0.66
Homogeneity: Q = 24.38, df = 8, p-value = 0.00
Test of overall effect size: z = -0.16, p-value = 0.87

Overall rate difference = -0.15% (95%CI -1.93 – 1.63), p=0.87

Figure S21. Difference in rate of any adverse events in children aged ≤5 years between vaccine and placebo groups (1 month after 2nd dose).

Effect size of each study
Estimated overall effect size
Estimated overall confidence interval
Confidence interval of effect si
Overall effect size value

ID	Study	Effect Size	Std. Error	Lower	Upper	p-value	Weight	Weight (%)
1	Sinovac	-3.14	1.02	-5.14	-1.13	0.00	0.34	5.65
2	Sinovac	-5.48	1.42	-8.26	-2.71	0.00	0.26	4.25
3	Sinovac	-5.86	0.58	-6.99	-4.72	0.00	0.45	7.45
4	Sinovac	-5.41	0.50	-6.39	-4.43	0.00	0.47	7.74
5	Sinovac	-4.71	0.15	-5.00	-4.42	0.00	0.52	8.67
8	CAMS	-3.04	0.16	-3.36	-2.73	0.00	0.52	8.66
9	CAMS	-4.50	0.12	-4.74	-4.26	0.00	0.53	8.70
13	WIBP	-4.08	0.71	-5.48	-2.68	0.00	0.42	6.91
14	WIBP	-6.24	0.32	-6.86	-5.62	0.00	0.50	8.33
15	WIBP	-6.25	0.38	-6.99	-5.51	0.00	0.49	8.15
20	Medigen	-1.69	0.07	-1.83	-1.56	0.00	0.53	8.75
22	CAMS or Sinovac	-3.72	0.38	-4.47	-2.97	0.00	0.49	8.14
24	Sinovac	-3.44	0.19	-3.81	-3.06	0.00	0.52	8.60
	Overall	-4.39	0.41	-5.18	-3.59	0.00		

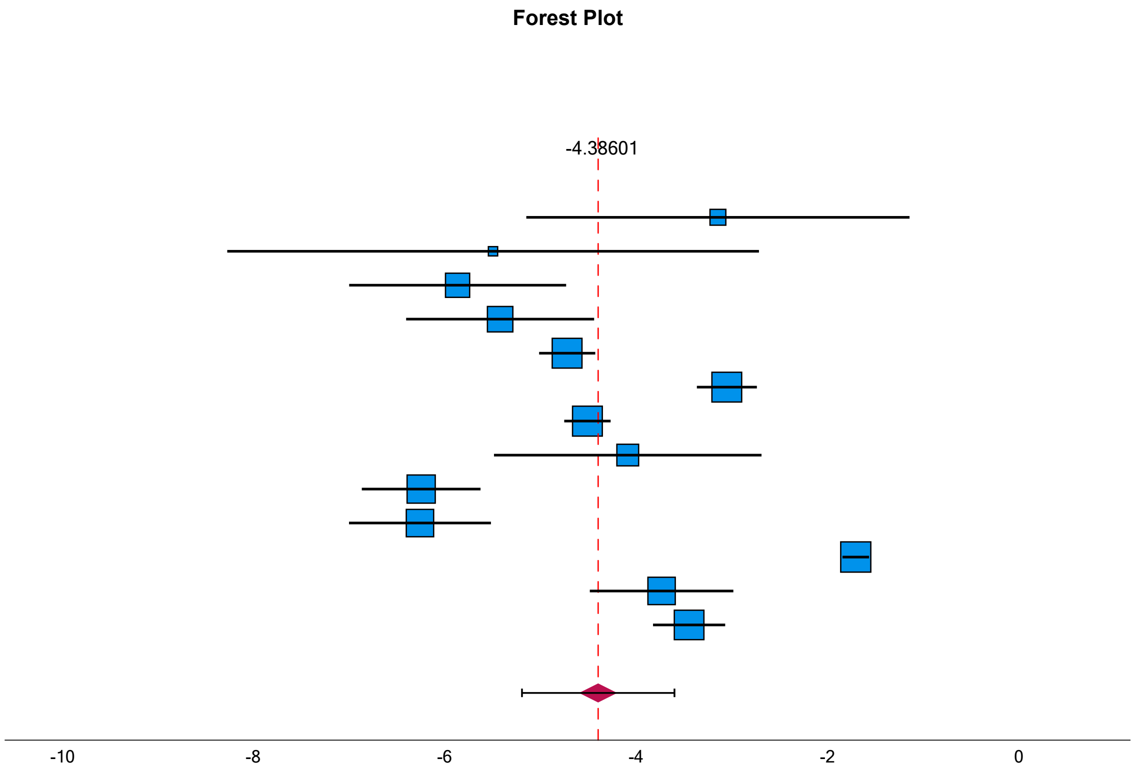

Model: Random-effects model
Heterogeneity: Tau-squared = 1.88, H-squared = 46.73, I-squared = 0.98
Homogeneity: Q = 856.57, df = 12, p-value = 0.00
Test of overall effect size: z = -10.79, p-value = 0.00

Figure S22. Natural logarithm of rate of serious adverse events in children aged ≤5 years in vaccine group (1 month after 2nd dose).

Effect size of each study
Estimated overall effect size
Estimated overall confidence interval
Confidence interval of effect si
Overall effect size value

ID	Study	Effect Size	Std. Error	Lower	Upper	p-value	Weight	Weight (%)
1	Placebo	-3.14	1.02	-5.14	-1.13	0.00	0.40	7.43
2	Placebo	-5.48	1.42	-8.26	-2.71	0.00	0.29	5.36
3	Placebo	-5.16	0.71	-6.55	-3.77	0.00	0.51	9.49
4	Placebo	-5.71	1.42	-8.49	-2.94	0.00	0.29	5.36
5	Placebo	-4.71	0.15	-5.00	-4.41	0.00	0.68	12.59
9	Placebo	-3.84	0.09	-4.02	-3.67	0.00	0.69	12.71
13	Placebo	-4.08	0.71	-5.48	-2.68	0.00	0.51	9.46
14	Placebo	-5.28	0.20	-5.66	-4.89	0.00	0.67	12.45
15	Placebo	-5.10	0.21	-5.52	-4.68	0.00	0.67	12.39
20	Placebo	-1.70	0.07	-1.83	-1.56	0.00	0.69	12.74
Overall		-4.30	0.43	-5.14	-3.45	0.00		

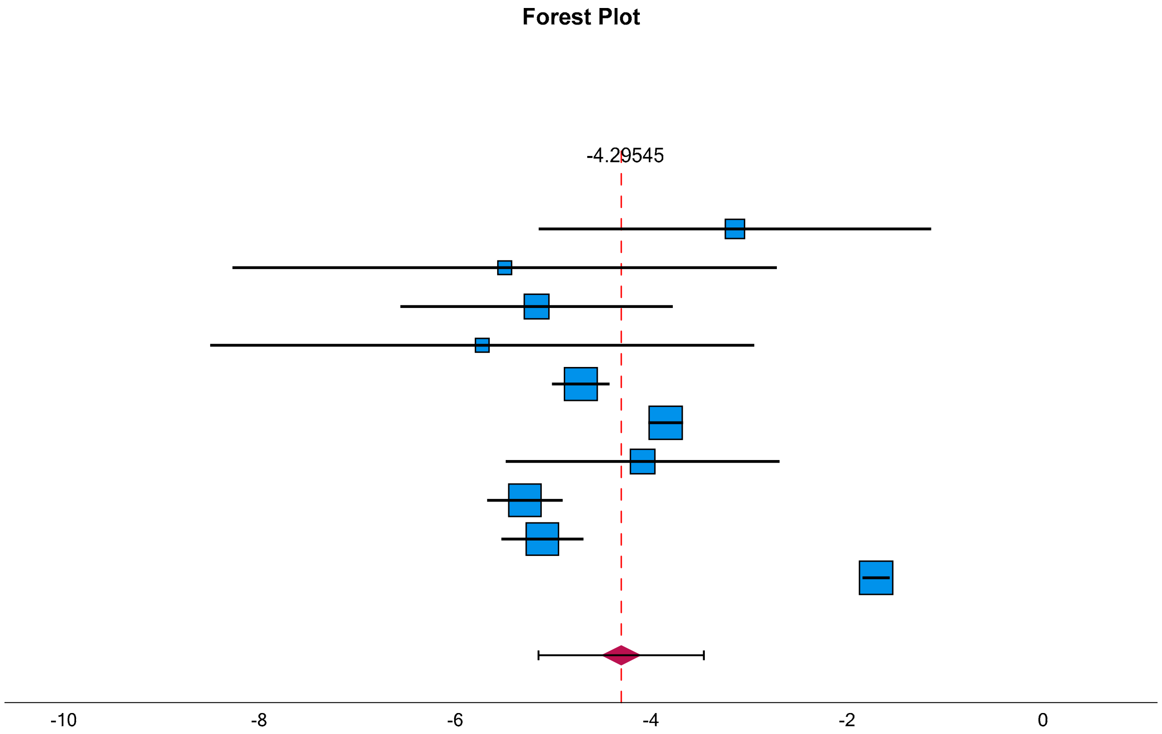

Model: Random-effects model
Heterogeneity: Tau-squared = 1.45, H-squared = 46.59, I-squared = 0.98
Homogeneity: Q = 805.03, df = 9, p-value = 0.00
Test of overall effect size: z = -9.98, p-value = 0.00

Figure S23. Natural logarithm of rate of serious adverse events in children aged ≤5 years in placebo group (1 month after 2nd dose).

Effect size of each study | Confidence interval of effect size
Estimated overall effect size | Overall effect size value
No-effect value | Estimated overall confidence int

ID	Risk Difference	Std. Error	Lower	Upper	p-value	Weight	Weight (%)
1	0.00	0.06	-0.11	0.11	1.00	299.85	0.06
2	0.00	0.01	-0.02	0.02	1.00	13241.74	2.79
3	-0.00	0.00	-0.01	0.01	0.51	37812.61	7.97
4	0.00	0.01	-0.01	0.01	0.74	28903.80	6.09
5	-0.00	0.00	-0.00	0.00	0.99	90800.12	19.13
9	-0.01	0.00	-0.01	-0.01	0.00	78497.10	16.54
13	0.00	0.02	-0.03	0.03	1.00	3563.18	0.75
14	-0.00	0.00	-0.01	-0.00	0.01	112784.58	23.76
15	-0.00	0.00	-0.01	-0.00	0.01	103212.85	21.74
20	0.00	0.01	-0.03	0.03	1.00	5572.65	1.17
Overall	-0.00	0.00	-0.01	-0.00	0.02		

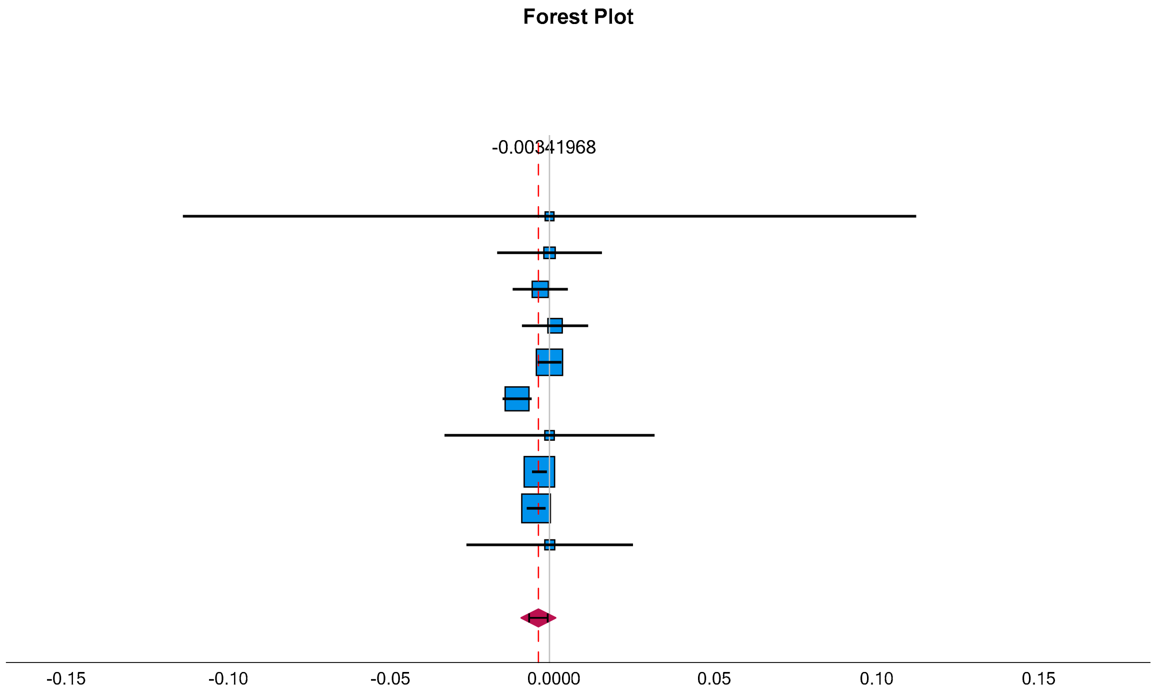

Model: Random-effects model
Heterogeneity: Tau-squared = 0.00, H-squared = 2.08, I-squared = 0.52
Homogeneity: Q = 13.12, df = 9, p-value = 0.16
Test of overall effect size: z = -2.36, p-value = 0.02

Overall rate difference = -0.34% (95%CI -0.63 - -0.06), p =0.02

Figure S24. Difference in rate of serious adverse events in children aged ⩽5 years between vaccine and placebo groups (1 month after 2nd dose).

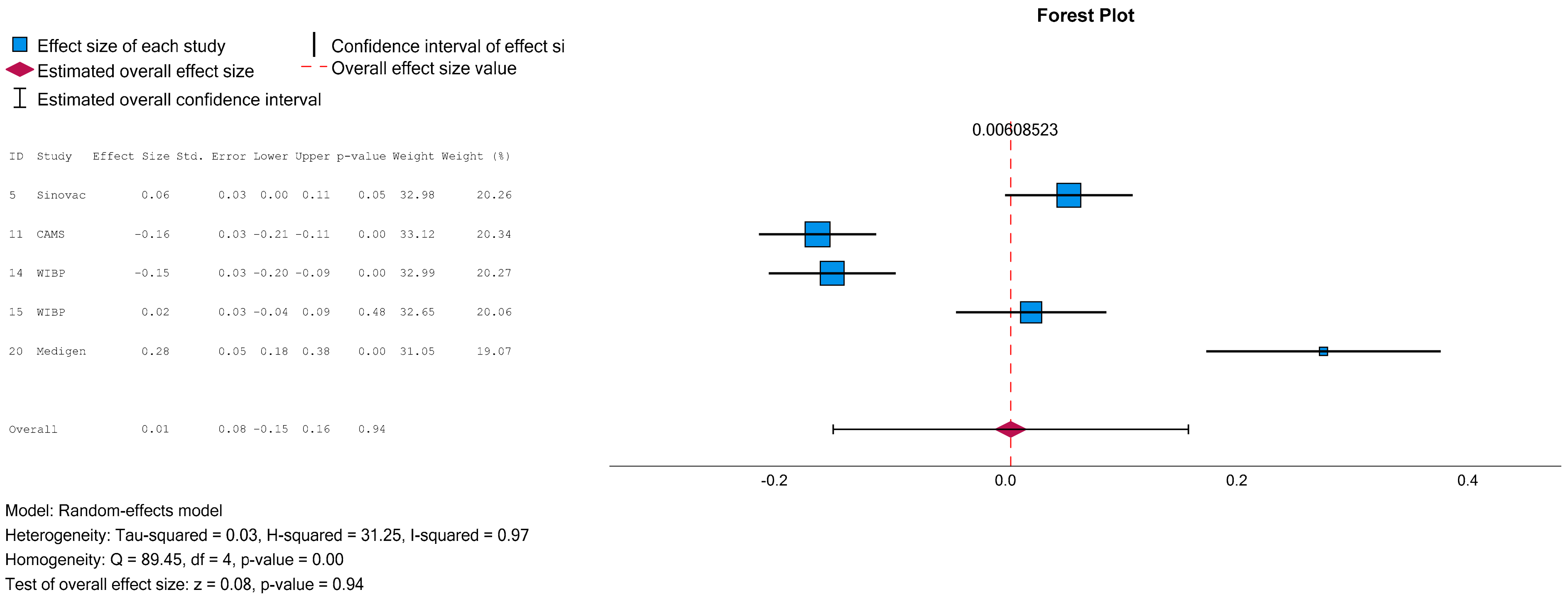

Figure S25. Natural logarithm of rate of solicited adverse events in children aged ≤5 years in vaccine group (7 days after 2nd dose).

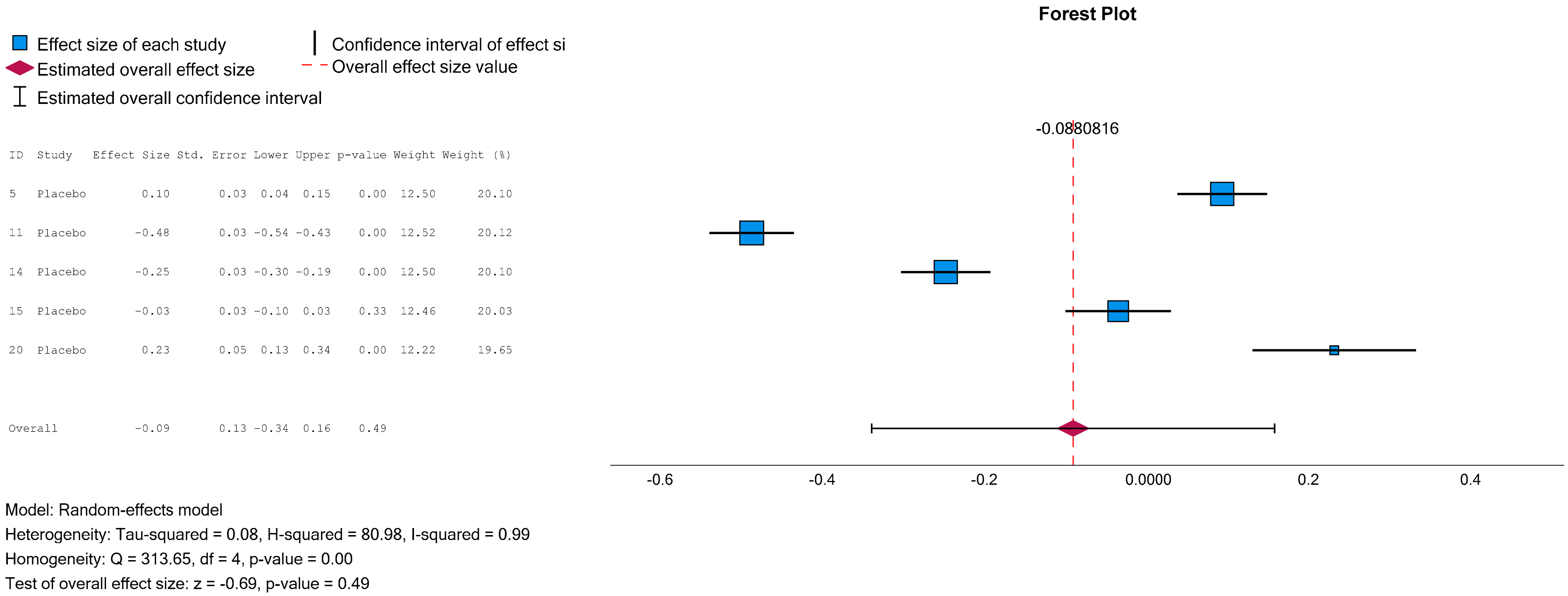

Figure S26. Natural logarithm of rate of solicited adverse events in children aged ≤5 years in placebo group (7 days after 2nd dose).

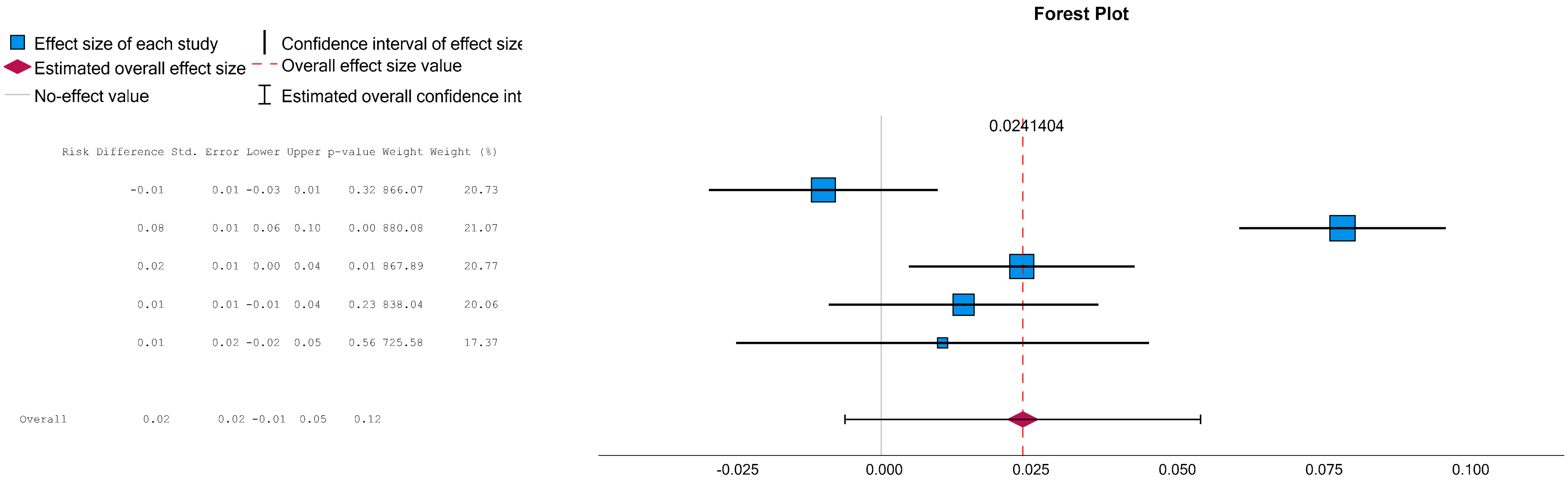

ID	Risk Difference	Std. Error	Lower	Upper	p-value	Weight	Weight (%)
5	-0.01	0.01	-0.03	0.01	0.32	866.07	20.73
11	0.08	0.01	0.06	0.10	0.00	880.08	21.07
14	0.02	0.01	0.00	0.04	0.01	867.89	20.77
15	0.01	0.01	-0.01	0.04	0.23	838.04	20.06
20	0.01	0.02	-0.02	0.05	0.56	725.58	17.37
Overall	0.02	0.02	-0.01	0.05	0.12		

Model: Random-effects model
Heterogeneity: Tau-squared = 0.00, H-squared = 9.80, I-squared = 0.90
Homogeneity: Q = 48.76, df = 4, p-value = 0.00
Test of overall effect size: z = 1.56, p-value = 0.12

Overall rate difference = 2.41% (95%CI -0.62 – 5.45), p =0.12

Figure S27. Difference in rate of solicited adverse events in children aged ≤5 years between vaccine and placebo groups (7 days after 2nd dose).

Effect size of each study
Estimated overall effect size
Estimated overall confidence interval
Confidence interval of effect si
Overall effect size value

ID	Study	Effect Size	Std. Error	Lower	Upper	p-value	Weight	Weight (%)
4	Sinovac	-5.16	0.41	-5.96	-4.36	0.00	0.15	14.28
5	Sinovac	0.27	0.03	0.21	0.33	0.00	0.15	14.64
11	CAMS	-0.33	0.03	-0.38	-0.28	0.00	0.15	14.64
15	WIBP	-0.01	0.03	-0.08	0.05	0.68	0.15	14.63
20	Medigen	0.58	0.05	0.48	0.69	0.00	0.15	14.63
21	Enimmune	0.09	0.30	-0.49	0.67	0.77	0.15	14.45
22	CAMS or Sinovac	-5.69	1.00	-7.65	-3.73	0.00	0.13	12.73
	Overall	-1.37	0.99	-3.32	0.57	0.17		

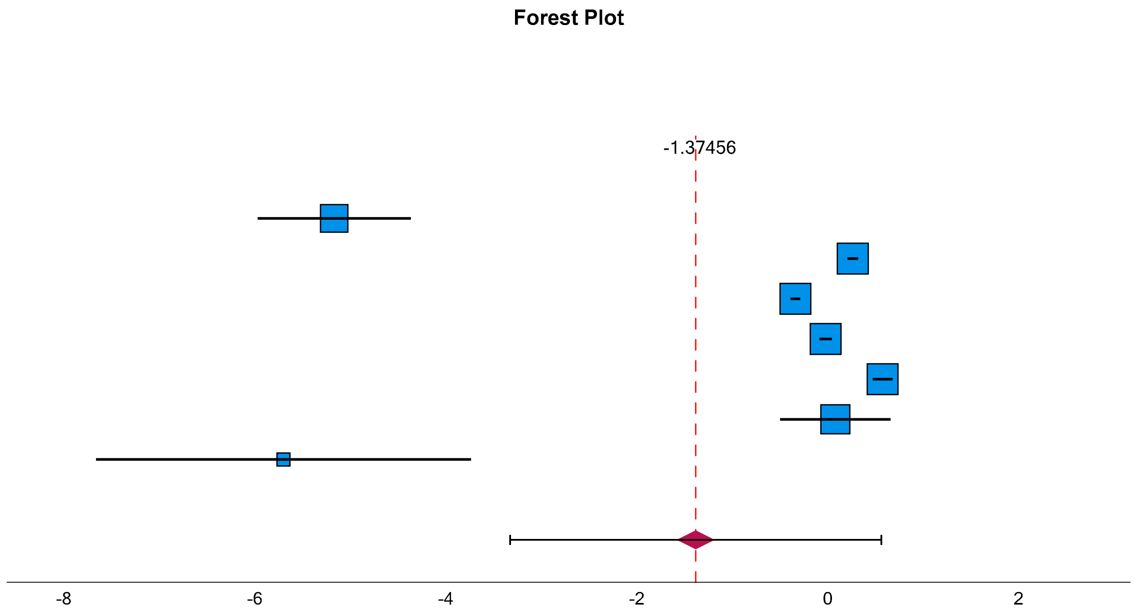

Model: Random-effects model
Heterogeneity: Tau-squared = 6.71, H-squared = 3146.95, I-squared = 1.00
Homogeneity: Q = 560.64, df = 6, p-value = 0.00
Test of overall effect size: z = -1.39, p-value = 0.17

Figure S28. Natural logarithm of rate of unsolicited adverse events in children aged ≤5 years in vaccine group (1 month after 2nd dose).

Forest Plot

- Effect size of each study
- Estimated overall effect size
- Estimated overall confidence interval
- Confidence interval of effect si
- Overall effect size value

ID	Study	Effect Size	Std. Error	Lower	Upper	p-value	Weight	Weight (%)
4	Placebo	-4.05	0.41	-4.86	-3.24	0.00	0.28	19.23
5	Placebo	0.31	0.03	0.25	0.37	0.00	0.30	20.20
11	Placebo	-0.29	0.03	-0.34	-0.24	0.00	0.30	20.20
15	Placebo	-0.06	0.03	-0.13	0.01	0.07	0.30	20.20
20	Placebo	0.52	0.05	0.41	0.62	0.00	0.30	20.18
Overall		-0.68	0.82	-2.29	0.93	0.41		

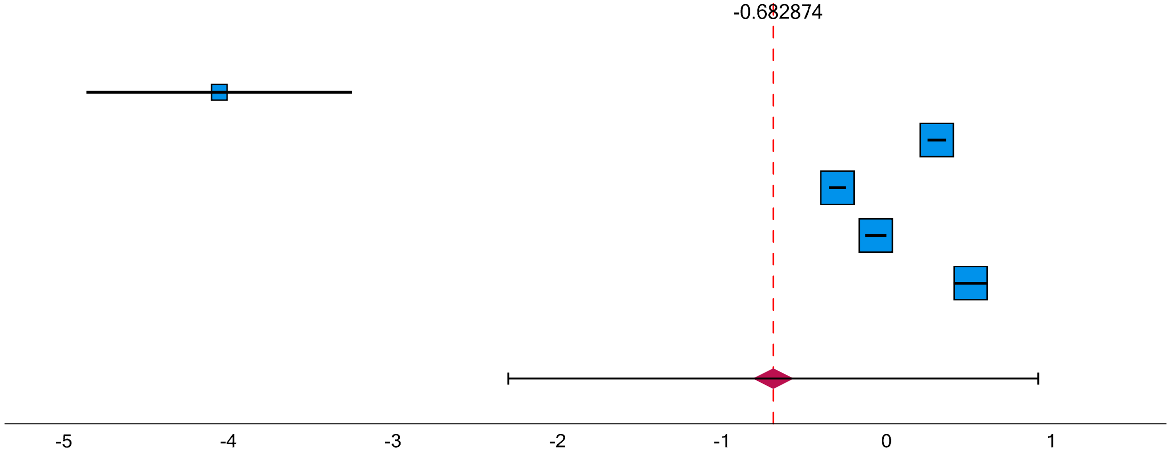

Model: Random-effects model

Heterogeneity: Tau-squared = 3.34, H-squared = 2341.63, I-squared = 1.00

Homogeneity: Q = 439.29, df = 4, p-value = 0.00

Test of overall effect size: z = -0.83, p-value = 0.41

Figure S29. Natural logarithm of rate of unsolicited adverse events in children aged ≤5 years in placebo group (1 month after 2nd dose).

Effect size of each study | Confidence interval of effect size
Estimated overall effect size | Overall effect size value
No-effect value | Estimated overall confidence int

ID	Risk Difference	Std. Error	Lower	Upper	p-value	Weight	Weight (%)
4	-0.01	0.01	-0.03	0.00	0.12	18669.51	36.04
5	-0.01	0.01	-0.03	0.01	0.31	10290.16	19.86
11	-0.01	0.01	-0.03	0.01	0.30	12294.64	23.73
15	0.01	0.01	-0.01	0.03	0.32	7262.80	14.02
20	0.02	0.02	-0.02	0.05	0.38	3285.68	6.34
Overall	-0.01	0.00	-0.01	0.00	0.19		

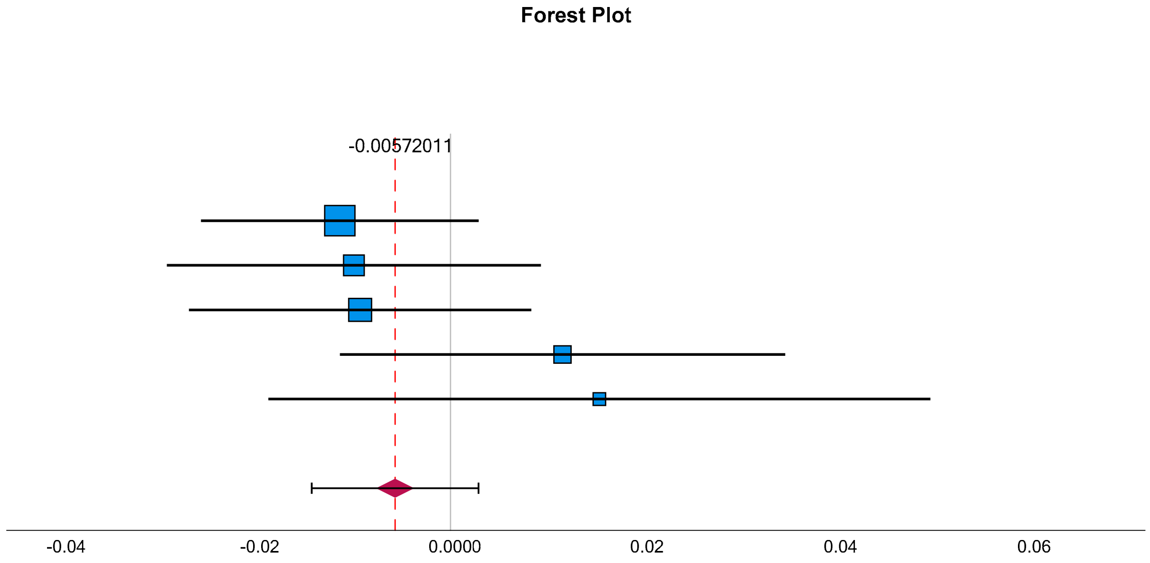

Model: Random-effects model
Heterogeneity: Tau-squared = 0.00, H-squared = 1.00, I-squared = 0.00
Homogeneity: Q = 4.59, df = 4, p-value = 0.33
Test of overall effect size: z = -1.30, p-value = 0.19

Overall rate difference = -0.57% (95%CI -1.43 – 0.29), p=0.19

Figure S30. Difference in rate of unsolicited adverse events in children aged ≤5 years between vaccine and placebo groups (1 month after 2nd dose).

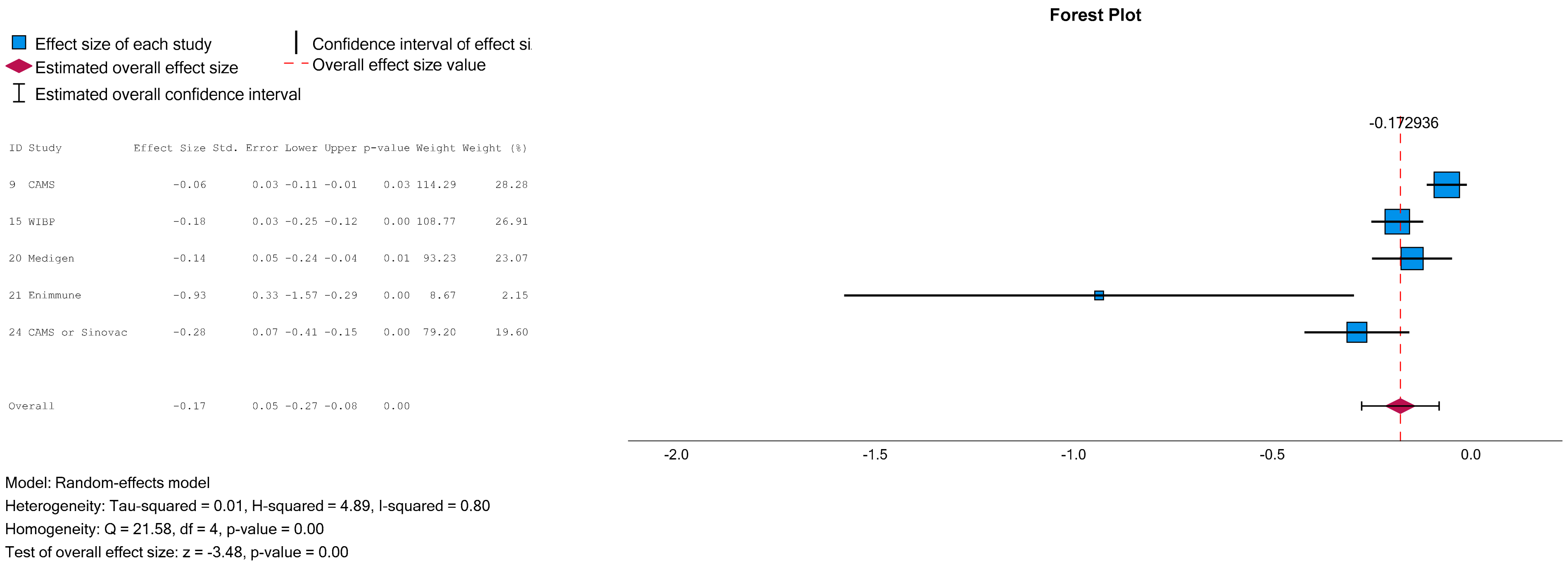

Figure S31. Natural logarithm of rate of systemic adverse events in children aged ≤5 years in vaccine group (7 days after 2nd dose).

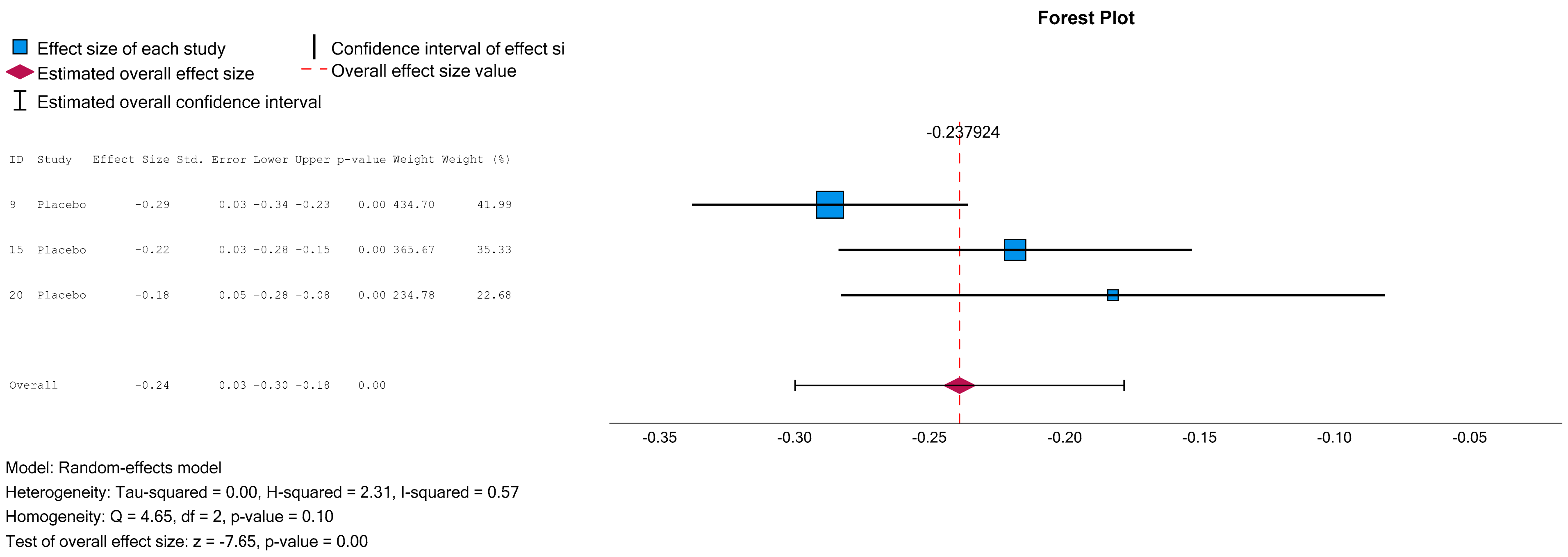

Figure S32. Natural logarithm of rate of systemic adverse events in children aged ≤5 years in placebo group (7 days after 2nd dose).

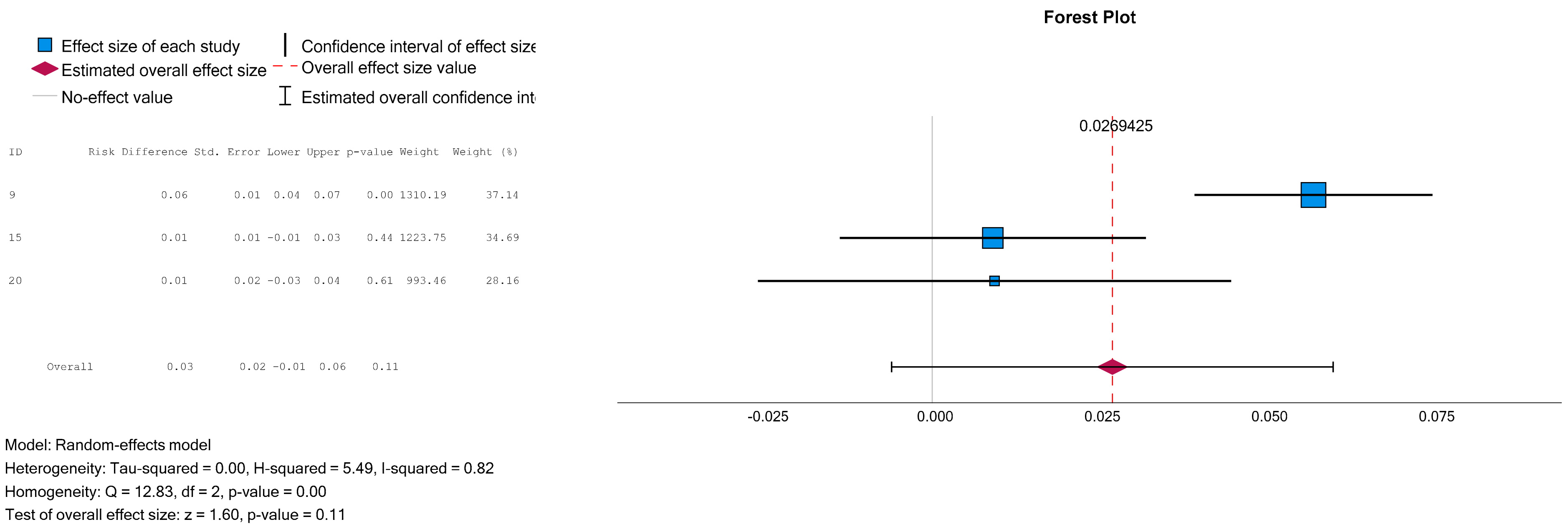

Overall rate difference = -2.69% (95%CI -0.61 – 5.99), p=0.11

Figure S33. Difference in rate of systemic adverse events in children aged ⩽5 years between vaccine and placebo groups (7 days after 2nd dose).

Effect size of each study
Estimated overall effect size
Estimated overall confidence interval
Confidence interval of effect si
Overall effect size value

ID	Study	Effect Size	Std. Error	Lower	Upper	p-value	Weight	Weight (%)
9	CAMS	-2.77	0.05	-2.88	-2.66	0.00	0.98	20.36
15	WIBP	-2.03	0.05	-2.13	-1.92	0.00	0.98	20.37
20	Medigen	-0.83	0.06	-0.94	-0.72	0.00	0.98	20.36
21	Enimmune	-0.35	0.30	-0.94	0.24	0.24	0.90	18.77
24	Sinovac	-2.40	0.12	-2.63	-2.16	0.00	0.96	20.14
	Overall	-1.69	0.46	-2.59	-0.80	0.00		

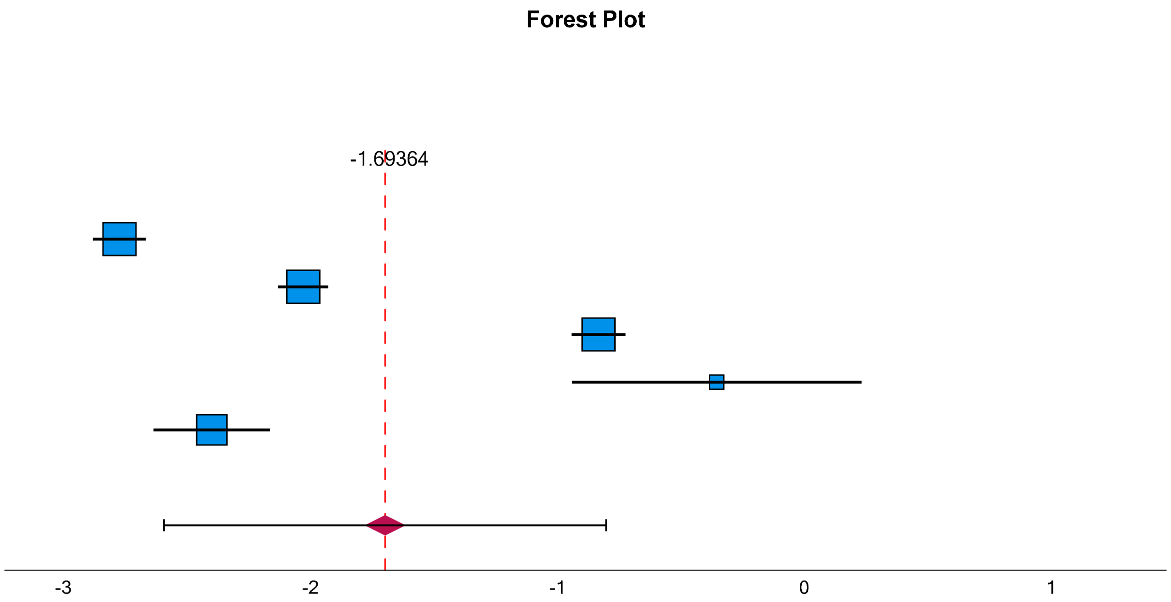

Model: Random-effects model
Heterogeneity: Tau-squared = 1.02, H-squared = 201.50, I-squared = 1.00
Homogeneity: Q = 669.83, df = 4, p-value = 0.00
Test of overall effect size: z = -3.71, p-value = 0.00

Figure S34. Natural logarithm of rate of local adverse events in children aged ≤5 years in vaccine group (7 days after 2nd dose).

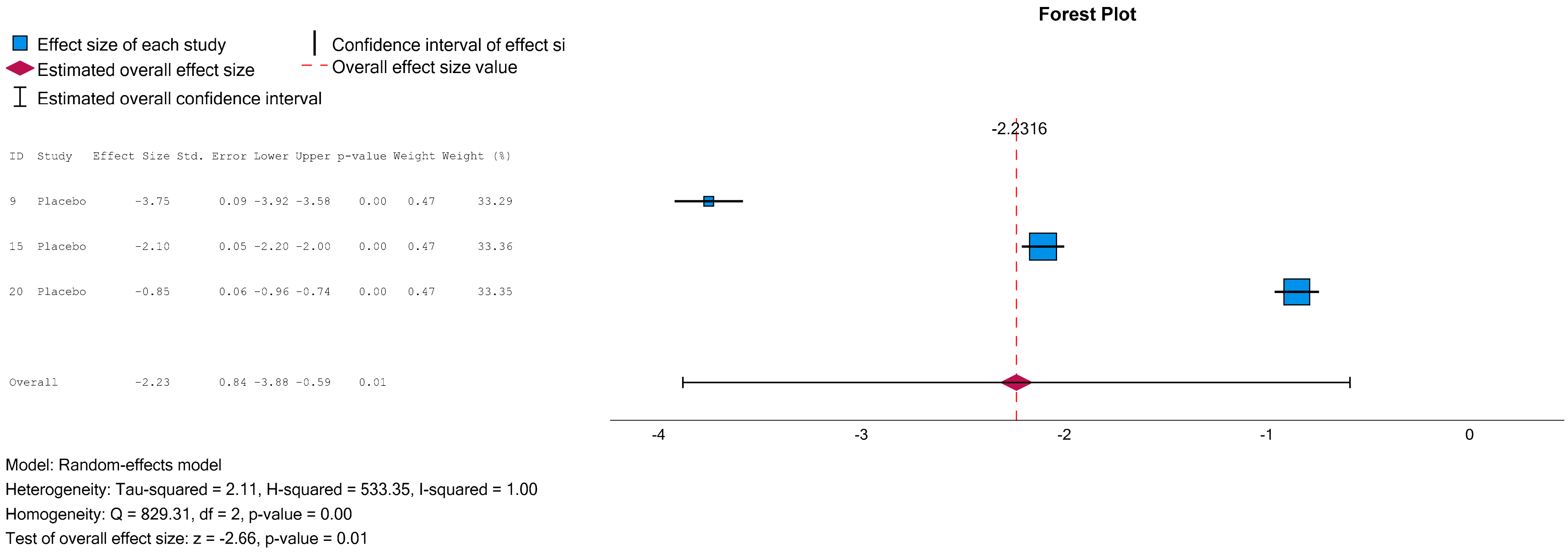

Figure S35. Natural logarithm of rate of local adverse events in children aged ≤5 years in placebo group (7 days after 2nd dose).

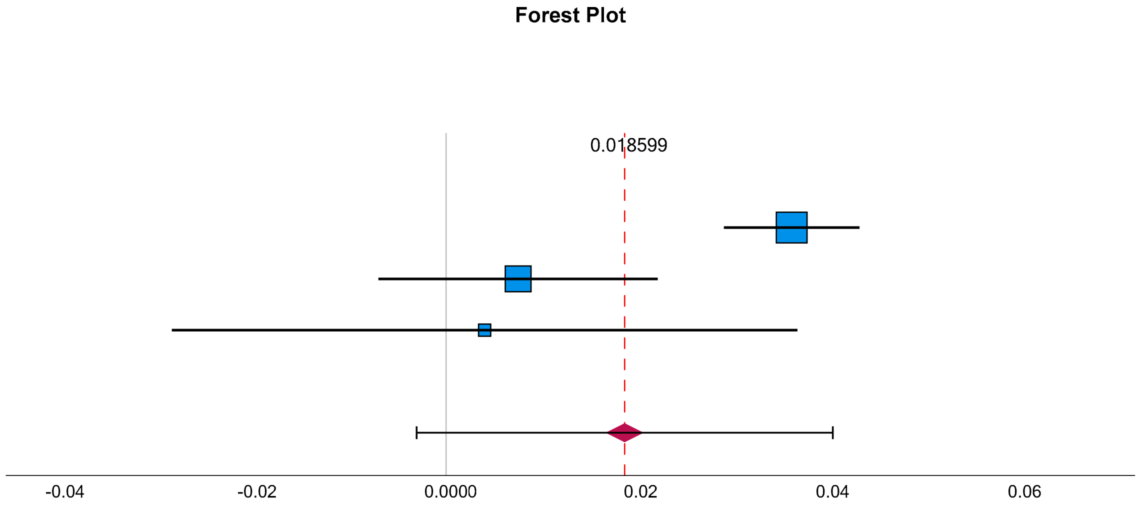

ID	Risk Difference	Std. Error	Lower	Upper	p-value	Weight	Weight (%)
9	0.04	0.00	0.03	0.04	0.00	3402.57	41.63
15	0.01	0.01	-0.01	0.02	0.31	2975.90	36.41
20	0.00	0.02	-0.03	0.04	0.81	1794.35	21.96
Overall	0.02	0.01	-0.00	0.04	0.09		

Model: Random-effects model

Heterogeneity: Tau-squared = 0.00, H-squared = 5.95, I-squared = 0.83

Homogeneity: Q = 14.38, df = 2, p-value = 0.00

Test of overall effect size: z = 1.68, p-value = 0.09

Overall rate difference = 1.86% (95%CI -0.31 – 4.03), p=0.09

Figure S36. Difference in rate of local adverse events in children aged ⩽5 years between vaccine and placebo groups (7 days after 2nd dose).

Table S1. Certainty of evidence assessment.

Outcome	Certainty Assessment						No. of patients		Effect (95% CI)	Certainty	Importance
	No. of RCTs	Risk of bias	Inconsistency	Indirectness	Imprecision	Other considerations	EV71 vaccines	Placebo			
VE against EV71 HFMD at 6 months	2	not serious	not serious	not serious	not serious	very strong association	10152	10143	Not pooled	⊕⊕⊕⊕ High	Critical
VE against EV71 HFMD at 1 year	3	not serious	not serious	not serious	not serious	very strong association	15944	15931	Not pooled	⊕⊕⊕⊕ High	Critical
VE against EV71 HFMD at 2 years	3	not serious	not serious	not serious	not serious	very strong association	10513	10490	Not pooled	⊕⊕⊕⊕ High	Critical
VE against all EV71 associated diseases at 6 months	2	not serious	not serious	not serious	not serious	very strong association	10156	10139	Not pooled	⊕⊕⊕⊕ High	Critical
VE against all EV71 associated diseases at 1 year	2	not serious	not serious	not serious	not serious	very strong association	10156	10139	Not pooled	⊕⊕⊕⊕ High	Critical
VE against all EV71 associated diseases at 2 years	1	not serious	not serious	not serious	not serious	very strong association	5056	5044	Not pooled	⊕⊕⊕⊕ High	Critical
Seropositive rate (titre >1:8) at 1 month (vaccine group)	13	not serious	not serious	not serious	not serious	publication bias strongly suspected very strong association	11229	-	99.19% (98.15 - 99.65)	⊕⊕⊕⊕ High	Important
Seropositive rate (titre >1:8) at 1 month (placebo group)	8	not serious	not serious	not serious	not serious	very strong association	-	6125	17.50% (10.58 - 27.57)	⊕⊕⊕⊕ High	Not important
Seropositive rate (titre >1:8) at 1 month among those with seronegative baseline (vaccine group)	8	not serious	not serious	not serious	not serious	very strong association	8217	-	99.30% (96.99 - 99.84)	⊕⊕⊕⊕ High	Important

Seropositive rate (titre >1:8) at 1 month among those with seronegative baseline (placebo group)	4	not serious	not serious	not serious	not serious	very strong association	-	4432	7.95% (3.41-17.44)	⊕⊕⊕⊕ High	Not important
Seropositive rate (titre >1:16) at 1 month (vaccine group)	4	not serious	not serious	not serious	not serious	very strong association	3492	-	98.52 (96.40 - 99.40)	⊕⊕⊕⊕ High	Important
Seropositive rate (titre >1:16) at 1 month (placebo group)	3	not serious	not serious	not serious	not serious	very strong association	-	1466	10.92% (2.01 - 42.25)	⊕⊕⊕⊕ High	Not important
Seropositive rate (titre >1:16) at 1 month among those with seronegative baseline (vaccine group)	3	not serious	not serious	not serious	not serious	very strong association	2635	-	99.25% (96.12 - 99.86)	⊕⊕⊕⊕ High	Important
Seropositive rate (titre >1:16) at 1 month among those with seronegative baseline (placebo group)	2	not serious	not serious	not serious	not serious	very strong association	-	725	2.08% (1.26 - 3.42)	⊕⊕⊕⊕ High	Not important
Seropositive rate (titre >1:32) at 1 month (vaccine group)	8	not serious	not serious	not serious	not serious	very strong association	8106	-	97.01% (93.00 - 98.73)	⊕⊕⊕⊕ High	Important
Seropositive rate (titre >1:32) at 1 month (placebo group)	6	not serious	not serious	not serious	not serious	very strong association	-	6021	8.96% (3.62 – 20.52)	⊕⊕⊕⊕ High	Not important

Seropositive rate (titre >1:32) at 1 month among those with seronegative baseline (vaccine group)	5	not serious	not serious	not serious	not serious	very strong association	6168	-	96.24% (90.23 – 98.61)	⊕⊕⊕⊕ High	Important
Seropositive rate (titre >1:32) at 1 month among those with seronegative baseline (placebo group)	4	not serious	not serious	not serious	not serious	very strong association	-	4232	3.09 (1.32 - 7.05)	⊕⊕⊕⊕ High	Not important
Seroconversion rate at 1 month (vaccine group)	10	not serious	not serious	not serious	not serious	publication bias strongly suspected very strong association	6746	-	96.30% (92.71 - 98.17)	⊕⊕⊕⊕ High	Important
Seroconversion rate at 1 month (placebo group)	5	not serious	not serious	not serious	not serious	very strong association	-	1561	4.96% (2.37 - 10.08)	⊕⊕⊕⊕ High	Not important
Seroconversion rate at 1 month among those with seronegative baseline (vaccine group)	6	not serious	not serious	not serious	not serious	very strong association	4080	-	98.93% (96.85 - 99.64)	⊕⊕⊕⊕ High	Important
Seroconversion rate at 1 month among those with seronegative baseline (placebo group)	4	not serious	not serious	not serious	not serious	very strong association	-	720	3.63% (1.83 - 7.08)	⊕⊕⊕⊕ High	Not important
Ratio of GMT between vaccine and placebo groups at 1 month	12	not serious	not serious	not serious	not serious	publication bias strongly suspected very strong association	8514	7302	46.78 (26.18 - 83.61)	⊕⊕⊕⊕ High	Important

Ratio of GMT between vaccine and placebo groups at 1 month among those with seronegative baseline	7	not serious	not serious	not serious	not serious	very strong association	5493	4921	60.99 (28.58 - 130.17)	⊕⊕⊕⊕ High	Important
GMT in children aged 6-11 months vs aged 1-4 years at 1 month	4	not serious	not serious	not serious	not serious	none	1209 (aged 6-11 months) 2722 (aged 1-4 years)	-	1.76 (1.12 – 2.76)	⊕⊕⊕⊕ High	Not important
GMT in children aged 6-35 months vs aged 3-5 years at 1 month	3	not serious	not serious	not serious	not serious	none	1314 (aged 0-2 years) 978 (aged 3-5 years)	-	1.79 (0.93 – 3.46)	⊕⊕⊕⊕ High	Not important
GMT at 6 months (vaccine group) (C4 vaccines)	3	not serious	not serious	not serious	not serious	very strong association	1250	-	Not pooled	⊕⊕⊕⊕ High	Important
GMT at 6 months (placebo group) (C4 vaccines)	3	not serious	not serious	not serious	not serious	very strong association	-	1288	Not pooled	⊕⊕⊕⊕ High	Not important
GMT at 12 months (vaccine group) (C4 vaccines)	3	not serious	not serious	not serious	not serious	very strong association	1218	-	Not pooled	⊕⊕⊕⊕ High	Important
GMT at 12 months (placebo group) (C4 vaccines)	3	not serious	not serious	not serious	not serious	very strong association	-	1261	Not pooled	⊕⊕⊕⊕ High	Not important
GMT at 24 months (vaccine group) (C4 vaccines)	3	not serious	not serious	not serious	not serious	very strong association	1100	-	Not pooled	⊕⊕⊕⊕ High	Important

GMT at 24 months (placebo group) (C4 vaccines)	3	not serious	not serious	not serious	not serious	very strong association	-	1133	Not pooled	⊕⊕⊕⊕ High	Not important
GMT at 60 months (vaccine group) (C4 vaccines)	1	not serious	not serious	not serious	not serious	strong association	106	-	141.4 (100.0 - 182.8)	⊕⊕⊕⊕ High	Important
GMT at 60 months (placebo group) (C4 vaccines)	1	not serious	not serious	not serious	not serious	strong association	-	105	71.8 (48.0 - 95.7)	⊕⊕⊕⊕ High	Not important
GMT at 6 months (vaccine group) (B4 vaccines)	2	not serious	not serious	not serious	not serious	very strong association	1143	-	Not pooled	⊕⊕⊕⊕ High	Important
GMT at 6 months (placebo group) (B4 vaccines)	1	not serious	not serious	not serious	not serious	very strong association	-	1104	7.9 (7.2 - 8.6)	⊕⊕⊕⊕ High	Not important
GMT at 12 months (vaccine group) (B4 vaccines)	2	not serious	not serious	not serious	not serious	very strong association	1140	-	Not pooled	⊕⊕⊕⊕ High	Important
GMT at 12 months (placebo group) (B4 vaccines)	1	not serious	not serious	not serious	not serious	very strong association	-	1104	8.0 (7.3 - 8.8)	⊕⊕⊕⊕ High	Not important
Ratio of GMFI between vaccine and placebo groups at 1 month	5	not serious	not serious	not serious	not serious	very strong association	1578	1602	28.41 (22.18 - 36.39)	⊕⊕⊕⊕ High	Important
Rate of any AE at 1 month (vaccine group)	12	not serious	not serious	not serious	not serious	none	29274	-	55.43% (46.08 – 64.41)	⊕⊕⊕⊕ High	Important
Rate of any AE at 1 month (placebo group)	9	not serious	not serious	not serious	not serious	none	-	24388	58.57% (50.09 – 66.56)	⊕⊕⊕⊕ High	Not important
Difference in rate of any AE at 1 month between vaccine and placebo groups	9	not serious	not serious	not serious	not serious	none	25086	24388	-0.15% (-1.93 - 1.63)	⊕⊕⊕⊕ High	Important

Rate of serious AE at 1 month (vaccine group)	13	not serious	not serious	not serious	not serious	publication bias strongly suspected	25619	-	1.23% (0.58 - 2.69)	⊕⊕⊕◯ Moderate	Critical
Rate of serious AE at 1 month (placebo group)	10	not serious	not serious	not serious	not serious	none	-	22080	1.34% (0.58 - 3.07)	⊕⊕⊕⊕ High	Not important
Difference in rate of serious AE at 1 month between vaccine and placebo groups	10	not serious	not serious	not serious	not serious	none	23524	22080	-0.34% (-0.63 - -0.06)	⊕⊕⊕⊕ High	Critical
Rate of solicited AE at 1 month (vaccine group)	5	not serious	not serious	not serious	not serious	none	21311	-	50.15% (46.32 - 53.98)	⊕⊕⊕⊕ High	Important
Rate of solicited AE at 1 month (placebo group)	5	not serious	not serious	not serious	not serious	none	-	21315	47.80% (41.66 - 54.00)	⊕⊕⊕⊕ High	Not important
Difference in rate of solicited AE at 1 month between vaccine and placebo groups	5	not serious	not serious	not serious	not serious	none	21311	21315	2.41% (-0.62 - 5.45)	⊕⊕⊕⊕ High	Important
Rate of unsolicited AE at 1 month (vaccine group)	7	not serious	not serious	not serious	not serious	none	17587	-	20.19% (350 - 63.83)	⊕⊕⊕⊕ High	Important
Rate of unsolicited AE at 1 month (placebo group)	5	not serious	not serious	not serious	not serious	none	-	16542	33.56% (9.17 - 71.65)	⊕⊕⊕⊕ High	Not important
Difference in rate of unsolicited AE at 1 month between vaccine and placebo groups	5	not serious	not serious	not serious	not serious	none	17244	16542	-0.57% (-1.43 - 0.29)	⊕⊕⊕⊕ High	Important
Rate of systemic AE at 1 month (vaccine group)	5	not serious	not serious	not serious	not serious	none	12094	-	45.69% (43.28 - 48.11)	⊕⊕⊕⊕ High	Important

Rate of systemic AE at 1 month (placebo group)	3	not serious	not serious	not serious	not serious	none	-	11159	44.08% (42.58 - 45.59)	⊕⊕⊕⊕ High	Not important
Difference in rate of systemic AE at 1 month between vaccine and placebo groups	3	not serious	not serious	not serious	not serious	none	11150	11159	-2.69% (-0.61 - 5.99)	⊕⊕⊕⊕ High	Important
Rate of local AE at 1 month (vaccine group)	5	not serious	not serious	not serious	not serious	none	12094	-	15.53% (6.98 - 31.04)	⊕⊕⊕⊕ High	Important
Rate of local AE at 1 month (placebo group)	3	not serious	not serious	not serious	not serious	none	-	11159	9.69% (2.03 - 35.75)	⊕⊕⊕⊕ High	Not important
Difference in rate of local AE at 1 month between vaccine and placebo groups	3	not serious	not serious	not serious	not serious	none	11150	11159	1.86% (-0.31 - 4.03)	⊕⊕⊕⊕ High	Important

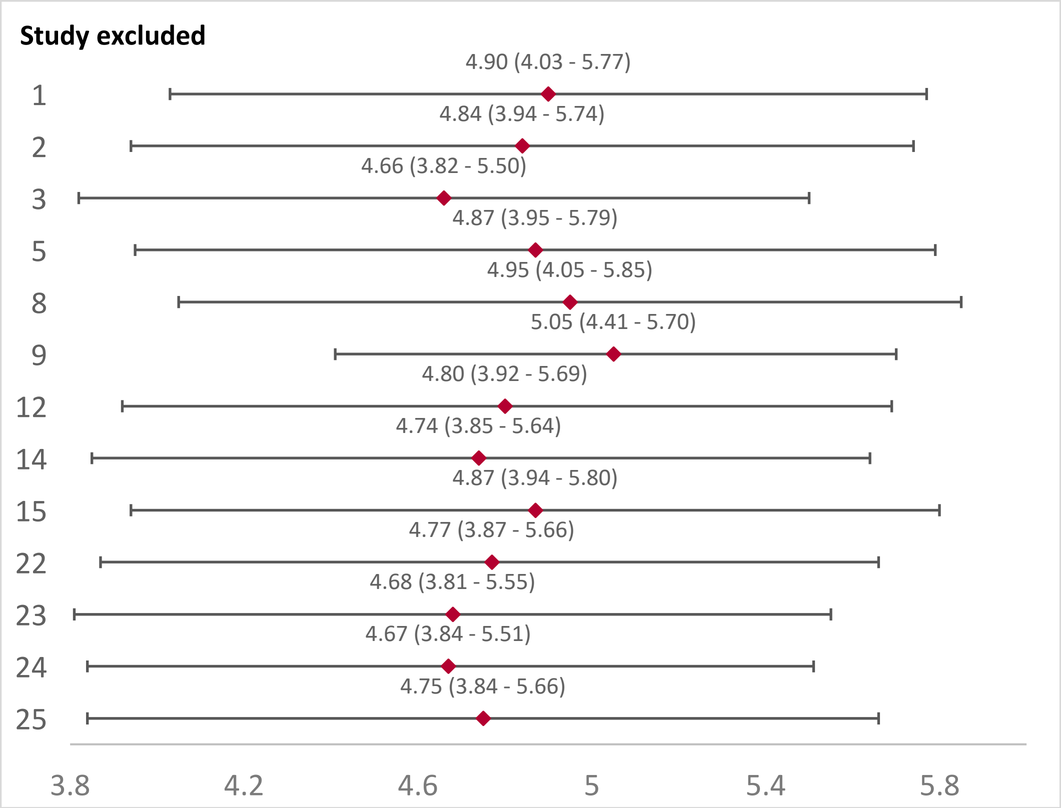

Figure S37. Sensitivity analysis for natural logarithm of proportion of children aged ⩽5 years in vaccine group with antibody titre ⩾1:8 (1 month after 2nd dose).

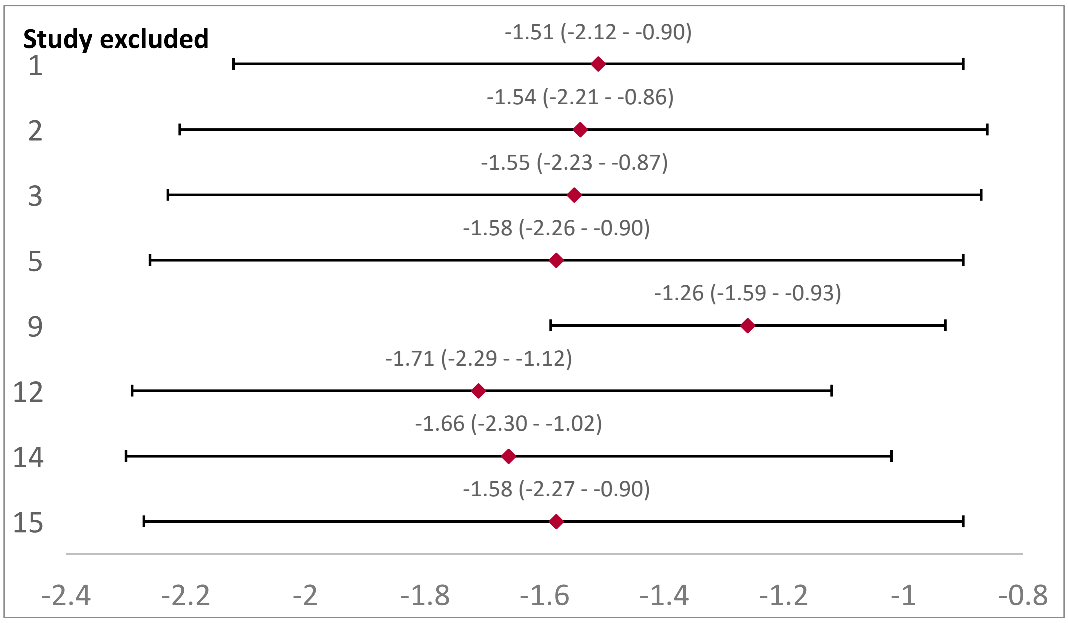

Figure S38. Sensitivity analysis for natural logarithm of proportion of children aged ⩽5 years in placebo group with antibody titre ⩾1:8 (1 month after 2nd dose).

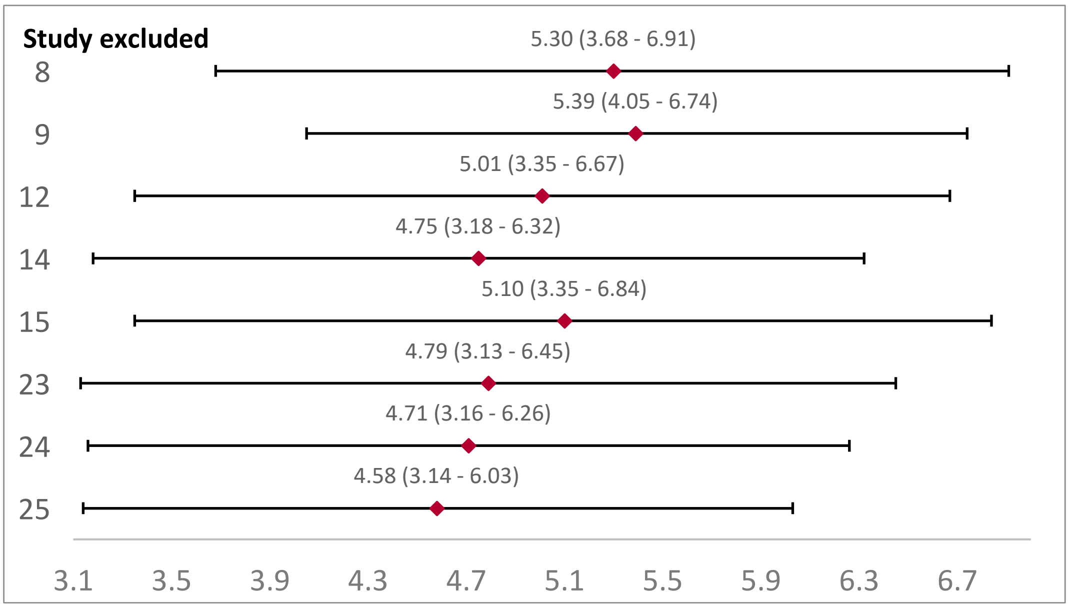

Figure S39. Sensitivity analysis for natural logarithm of proportion of children aged ≤5 years with seronegative baseline in vaccine group with antibody titre ≥1:8 (1 month after 2nd dose).

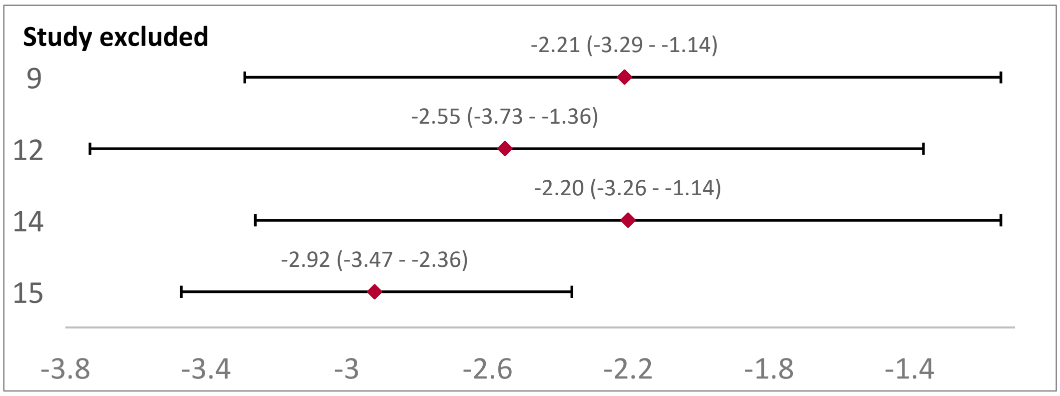

Figure S40. Sensitivity analysis for natural logarithm of proportion of children aged ≤5 years with seronegative baseline in placebo group with antibody titre ≥1:8 (1 month after 2nd dose).

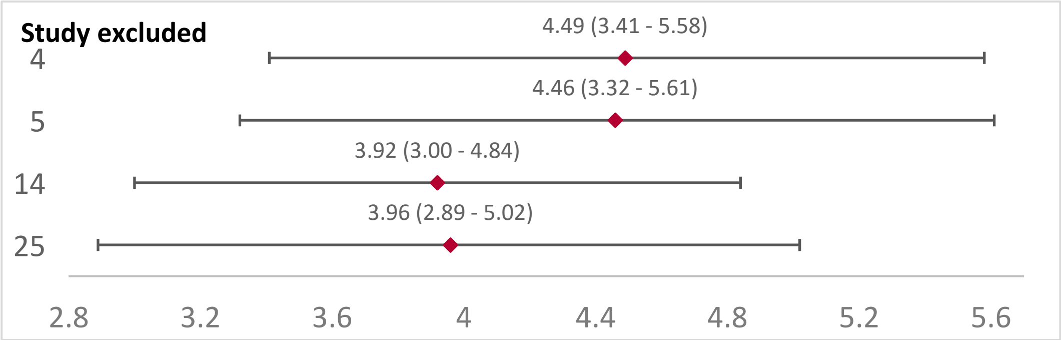

Figure S41. Sensitivity analysis for natural logarithm of proportion of children aged ≤5 years in vaccine group with antibody titre ≥1:16 (1 month after 2nd dose).

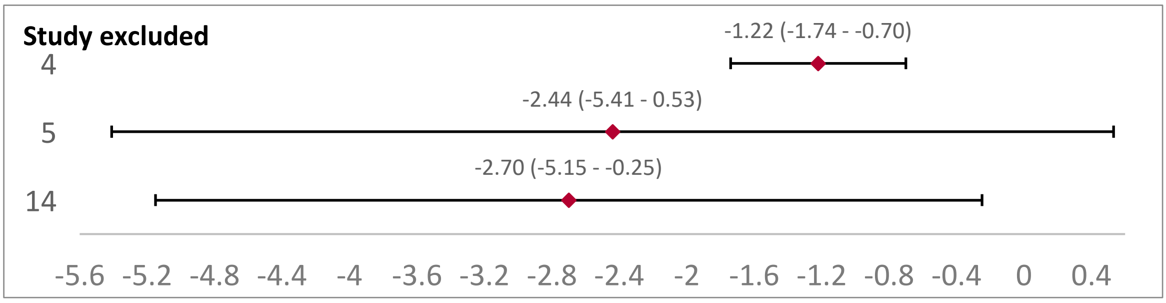

Figure S42. Sensitivity analysis for natural logarithm of proportion of children aged ⩽5 years in placebo group with antibody titre ⩾1:16 (1 month after 2nd dose).

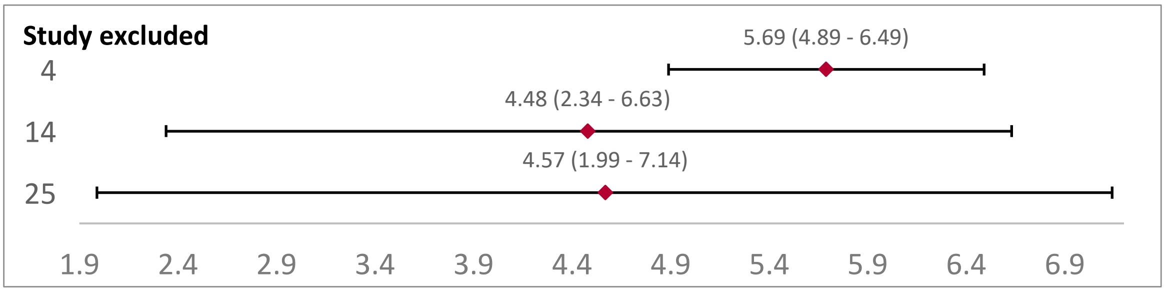

Figure S43. Sensitivity analysis for natural logarithm of proportion of children aged ⩽5 years with seronegative baseline in vaccine group with antibody titre ⩾1:16 (1 month after 2nd dose).

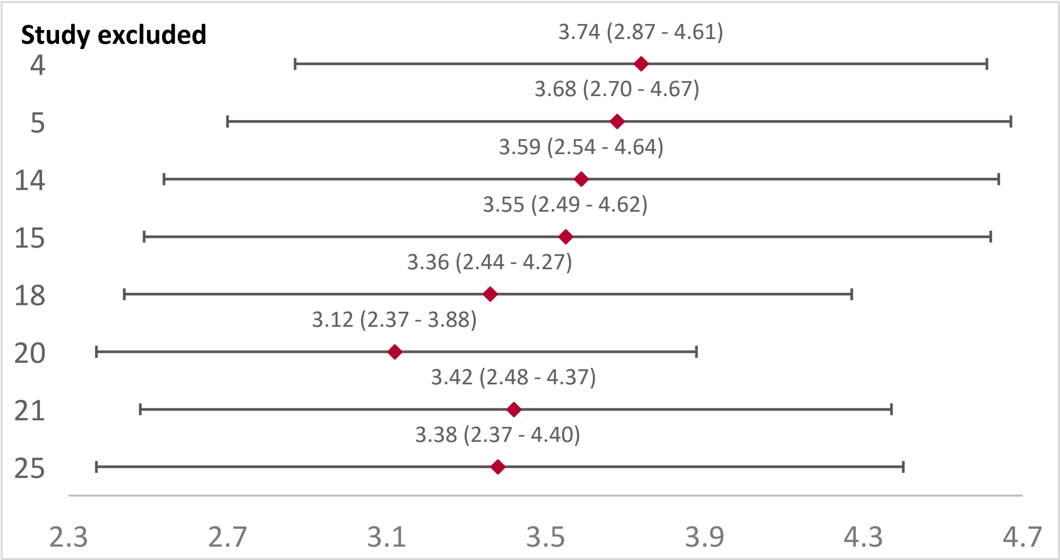

Figure S44. Sensitivity analysis for natural logarithm of proportion of children aged ⩽5 years in vaccine group with antibody titre ⩾1:32 (1 month after 2nd dose).

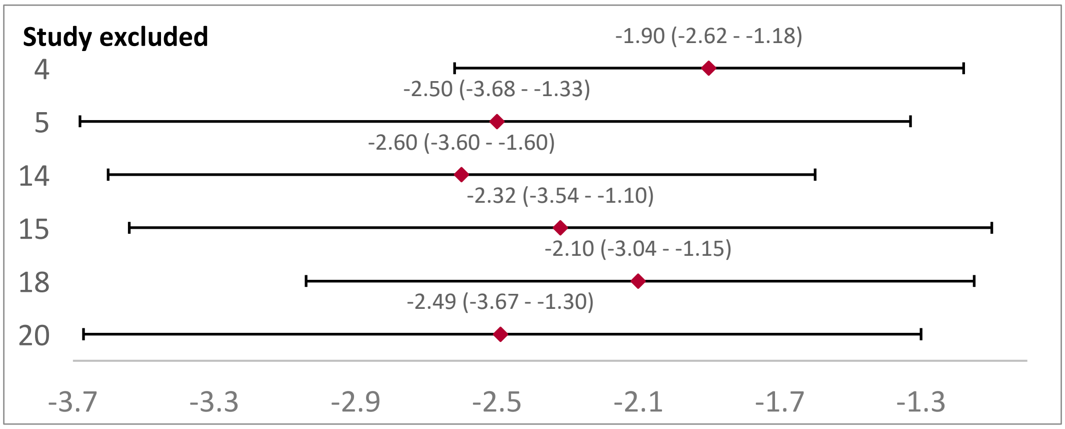

Figure S45. Sensitivity analysis for natural logarithm of proportion of children aged ⩽5 years in placebo group with antibody titre ⩾1:32 (1 month after 2nd dose).

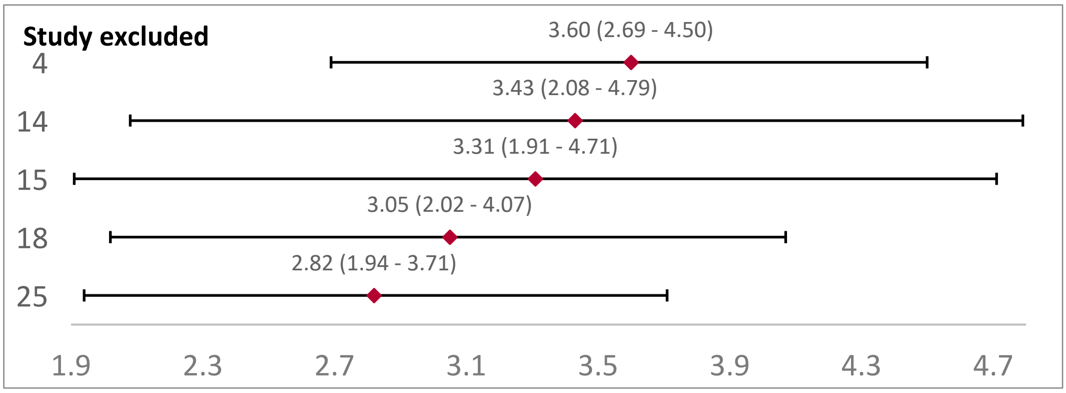

Figure S46. Sensitivity analysis for natural logarithm of proportion of children aged ⩽5 years with seronegative baseline in vaccine group with antibody titre ⩾1:32 (1 month after 2nd dose).

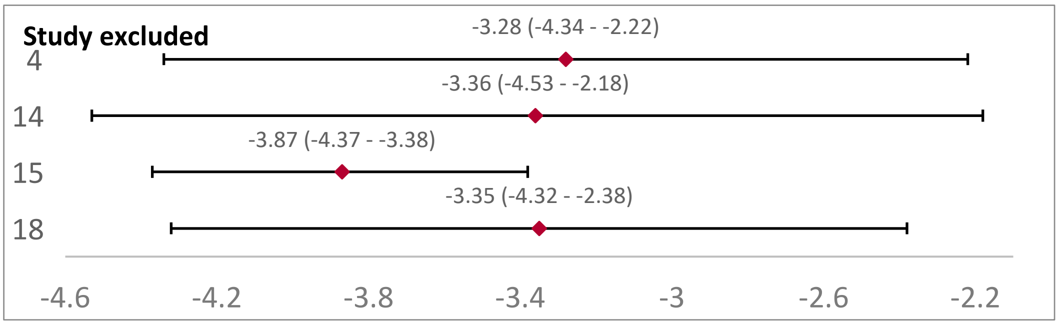

Figure S47. Sensitivity analysis for natural logarithm of proportion of children aged ⩽5 years with seronegative baseline in placebo group with antibody titre ⩾1:32 (1 month after 2nd dose).

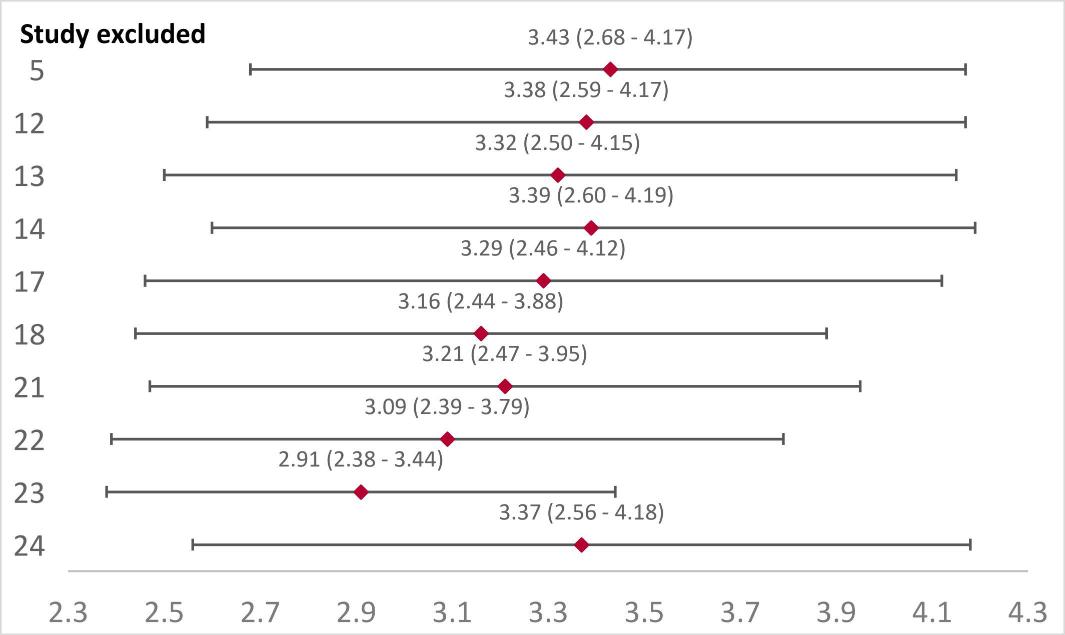

Figure S48. Sensitivity analysis for natural logarithm of seroconversion rate of children aged ≤5 years in vaccine group (1 month after 2nd dose).

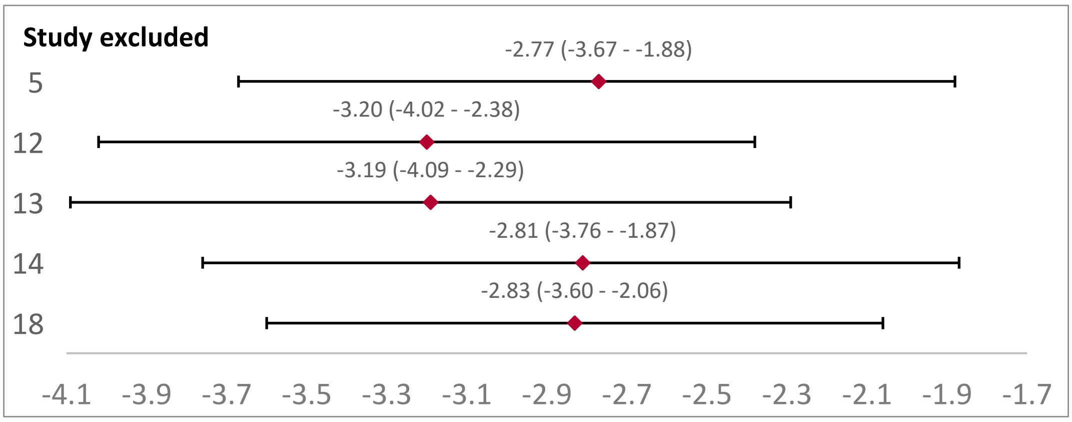

Figure S49. Sensitivity analysis for natural logarithm of seroconversion rate of children aged ≤5 years in placebo group (1 month after 2nd dose).

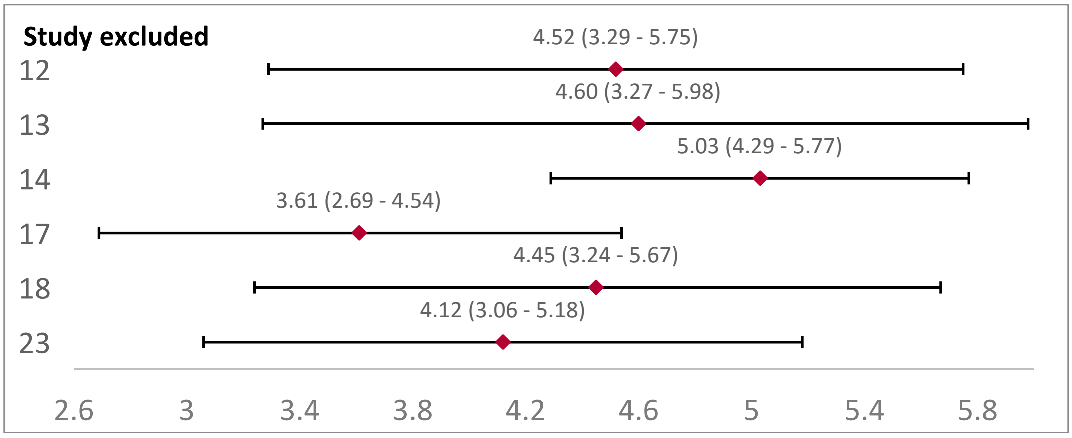

Figure S50. Sensitivity analysis for natural logarithm of seroconversion rate of children aged ≤5 years with seronegative baseline in vaccine group (1 month after 2nd dose).

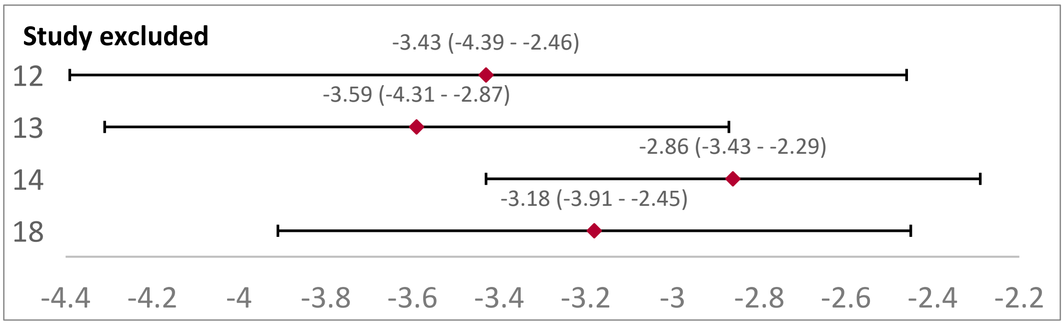

Figure S51. Sensitivity analysis for natural logarithm of seroconversion rate of children aged ≤5 years with seronegative baseline in placebo group (1 month after 2nd dose).

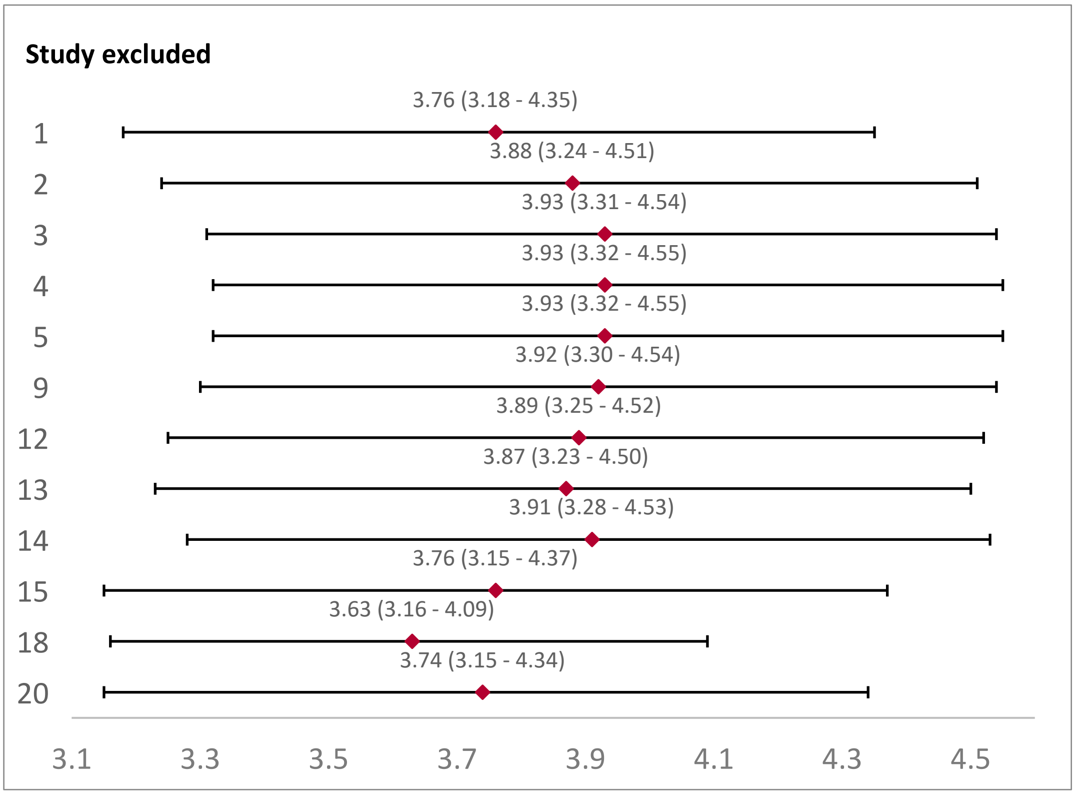

Figure S52. Sensitivity analysis for natural logarithm of ratio of GMT between vaccine and placebo groups in children aged ≤5 years (1 month after 2nd dose).

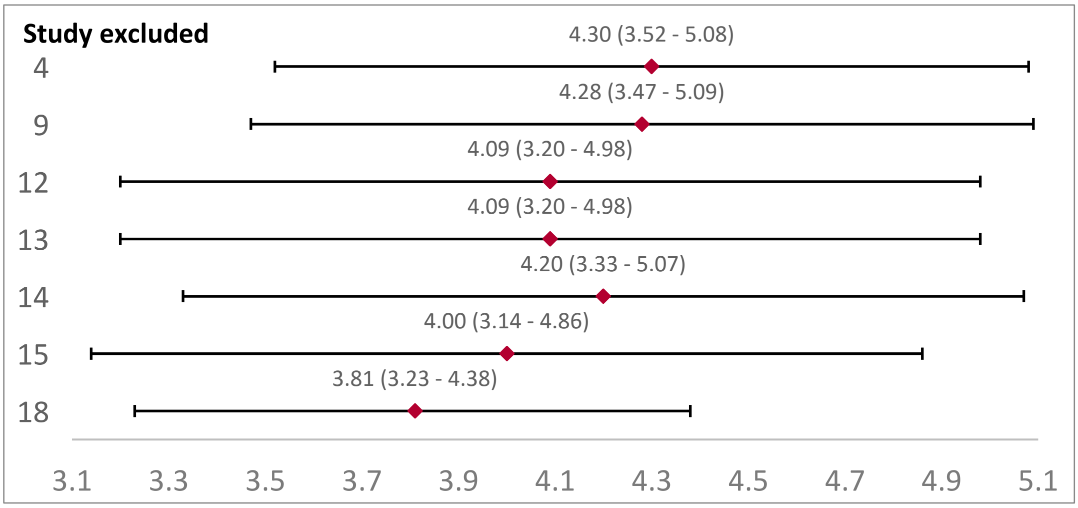

Figure S53. Sensitivity analysis for natural logarithm of ratio of GMT between vaccine and placebo groups in children aged ≤5 years with seronegative baseline (1 month after 2nd dose).

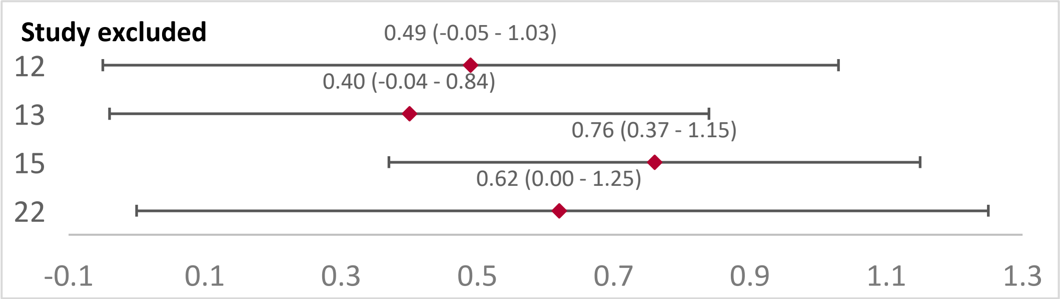

Figure S54. Sensitivity analysis for natural logarithm of ratio of GMT in children aged 6-11 months as compared to those aged 1-4 years (1 month after 2nd dose).

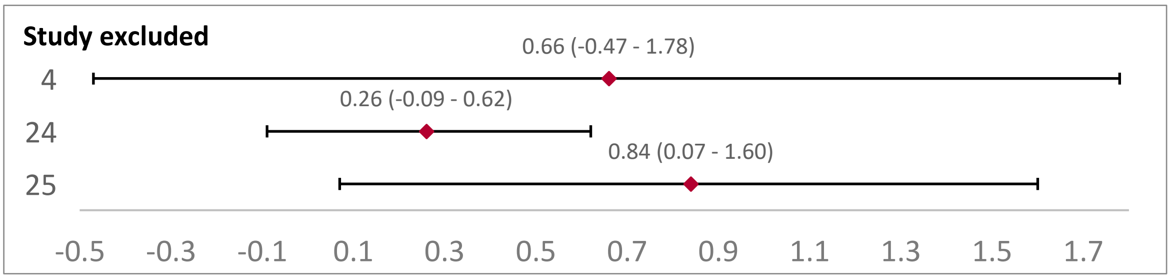

Figure S55. Sensitivity analysis for natural logarithm of ratio of GMT in children aged 6-35 months as compared to those aged 3-5 years (1 month after 2nd dose).

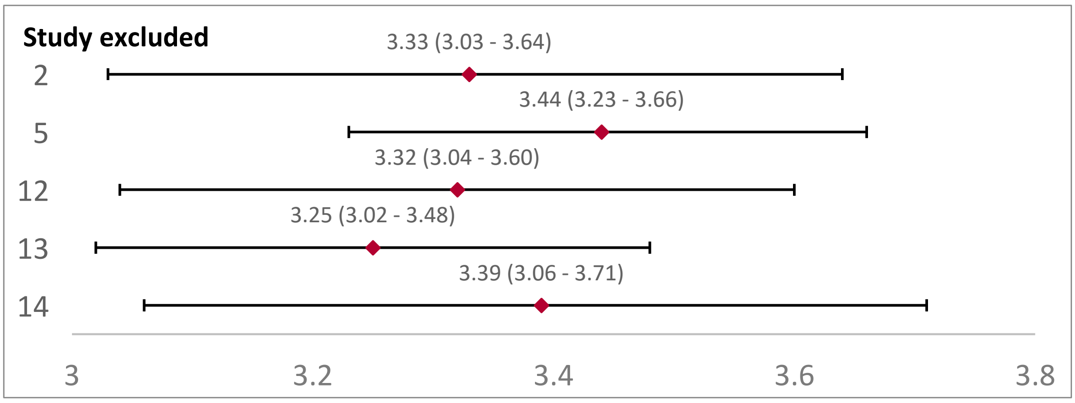

Figure S56. Sensitivity analysis for natural logarithm of ratio of GMFI between vaccine and placebo groups in children aged ≤5 years (1 month after 2nd dose).

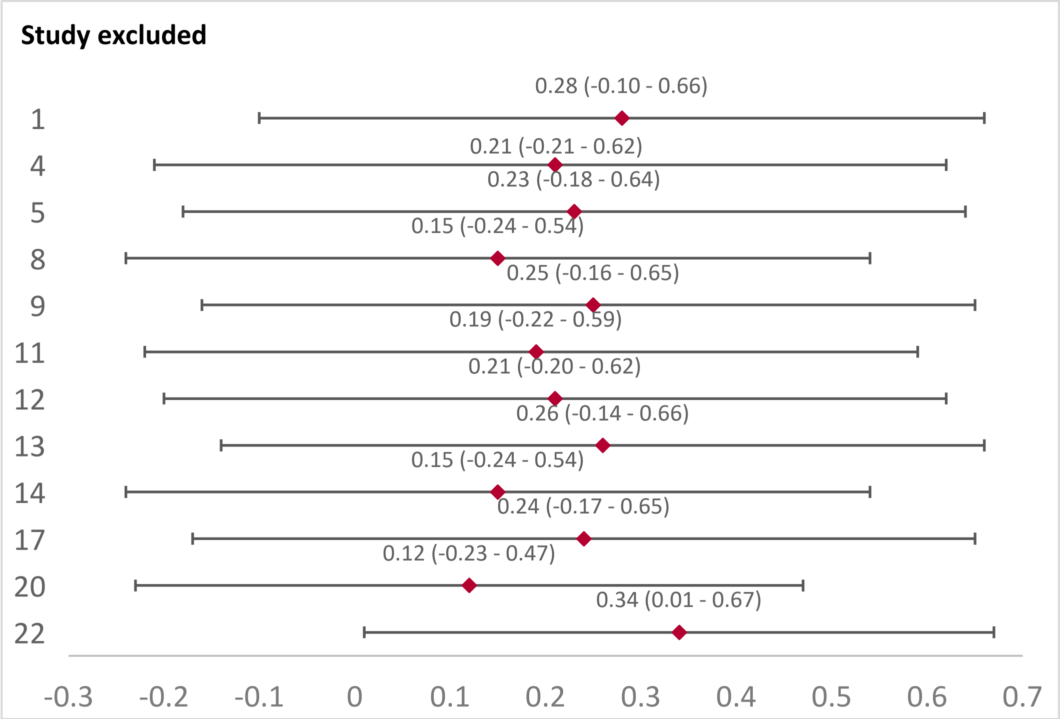

Figure S57. Sensitivity analysis for natural logarithm of rate of any adverse events in children aged ≤5 years in vaccine group (1 month after 2nd dose).

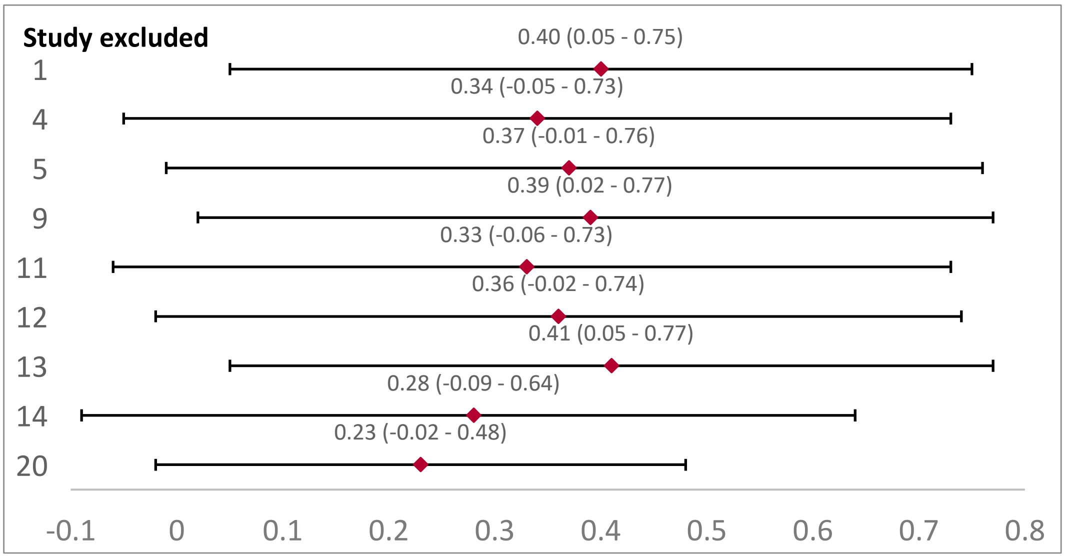

Figure S58. Sensitivity analysis for natural logarithm of rate of any adverse events in children aged ≤5 years in placebo group (1 month after 2nd dose).

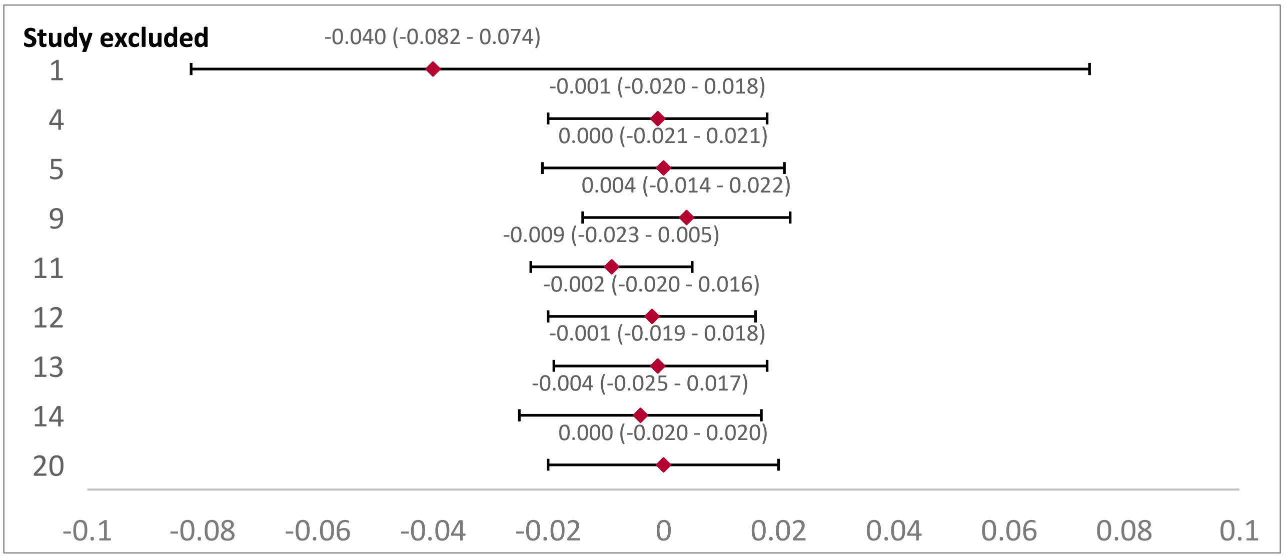

Figure S59. Sensitivity analysis for difference in rate of any adverse events in children aged ≤5 years between vaccine and placebo groups (1 month after 2nd dose).

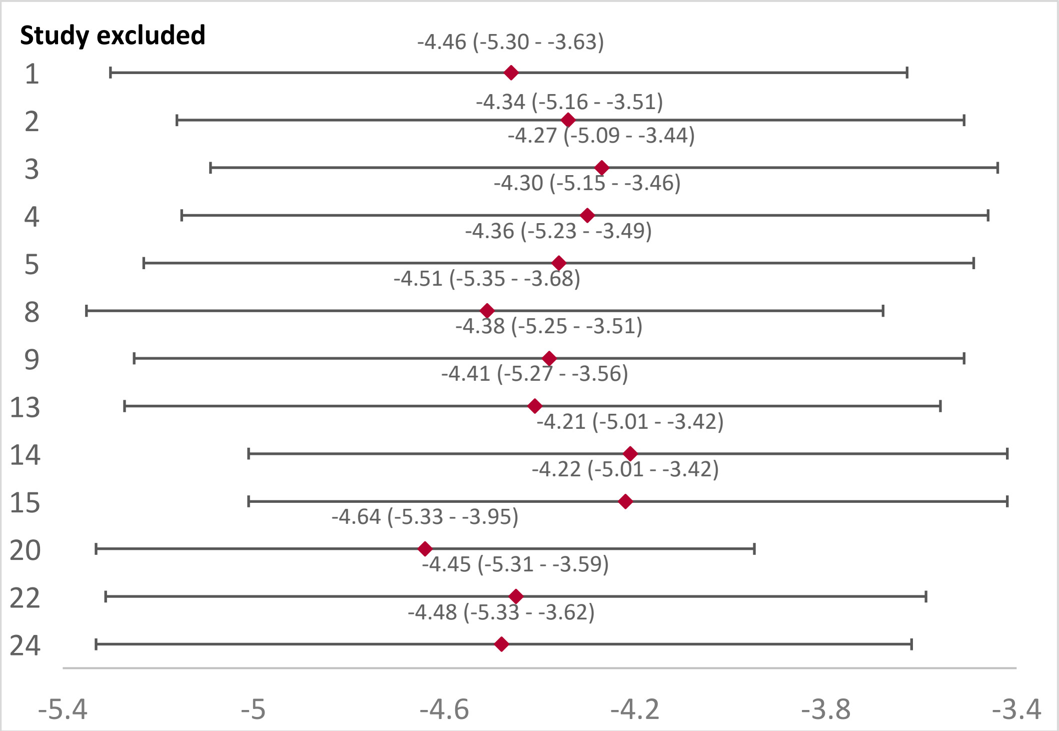

Figure S60. Sensitivity analysis for natural logarithm of rate of serious adverse events in children aged ⩽5 years in vaccine group (1 month after 2nd dose).

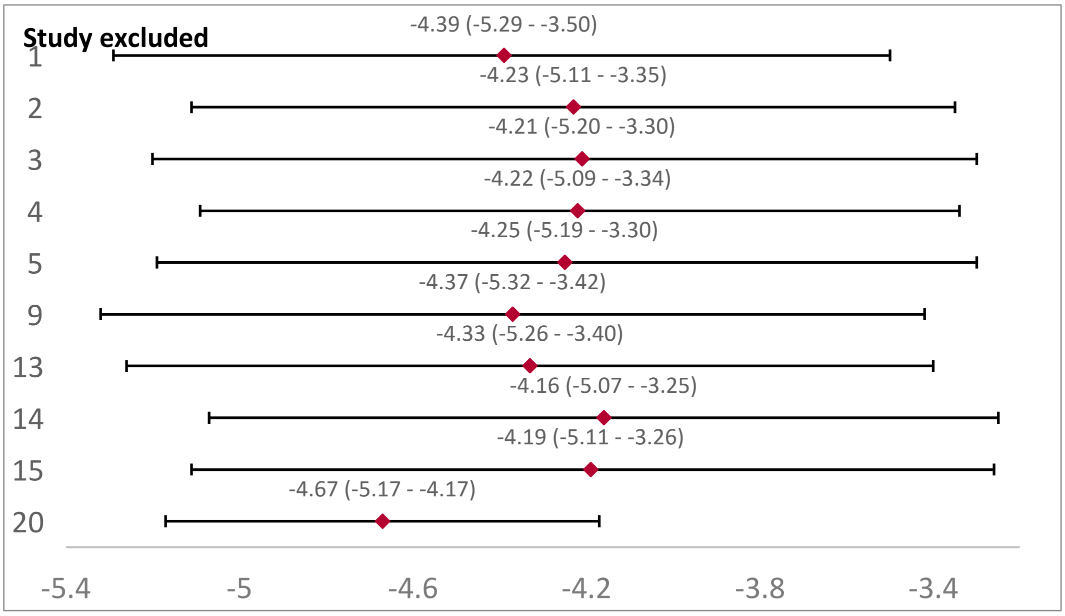

Figure S61. Sensitivity analysis for natural logarithm of rate of serious adverse events in children aged ⩽5 years in placebo group (1 month after 2nd dose).

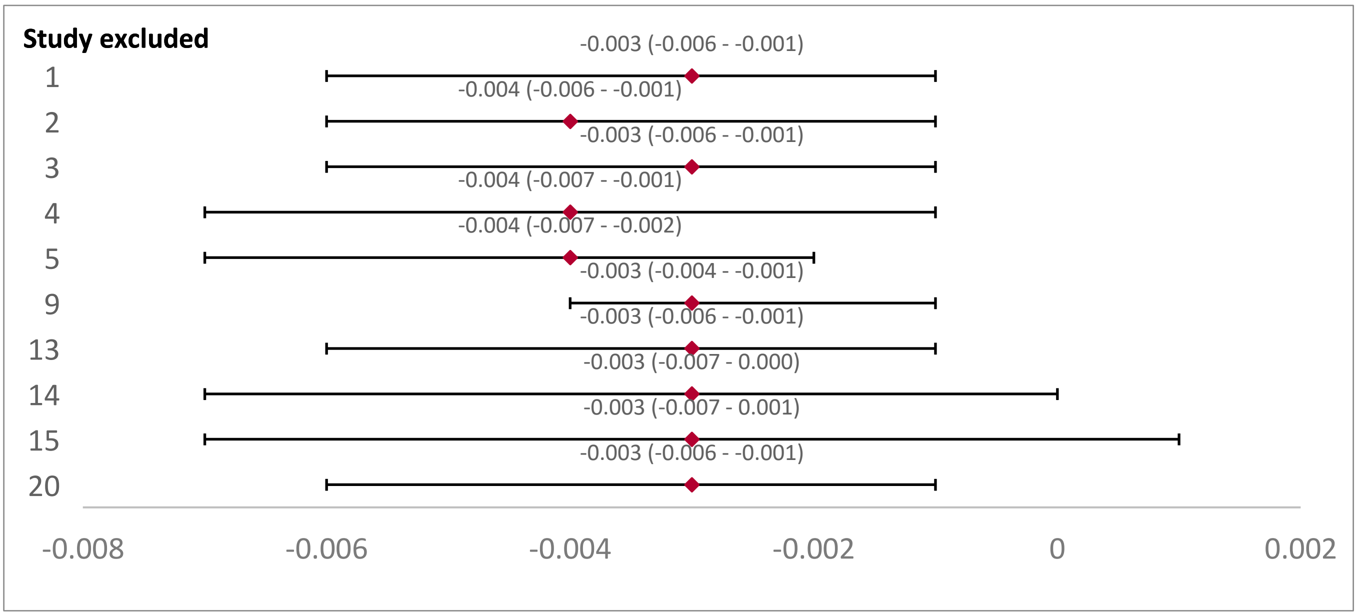

Figure S62. Sensitivity analysis for difference in rate of serious adverse events in children aged ⩽5 years between vaccine and placebo groups (1 month after 2nd dose).

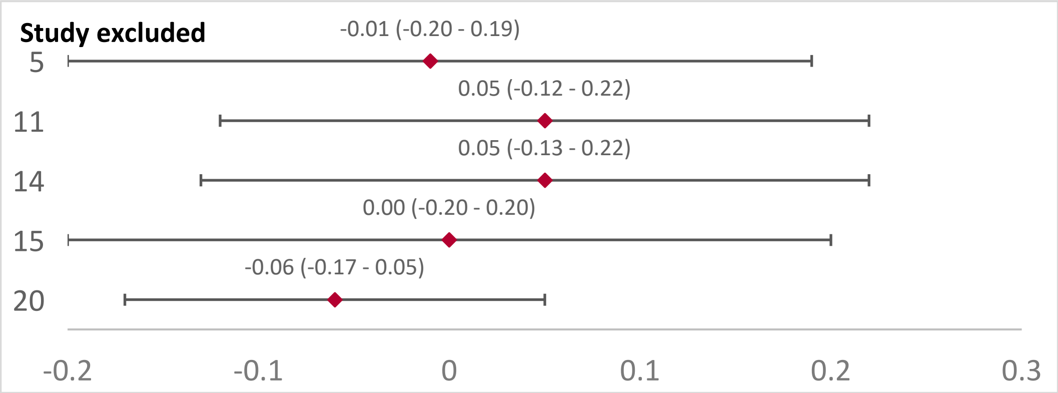

Figure S63. Sensitivity analysis for natural logarithm of rate of solicited adverse events in children aged ⩽5 years in vaccine group (7 days after 2nd dose).

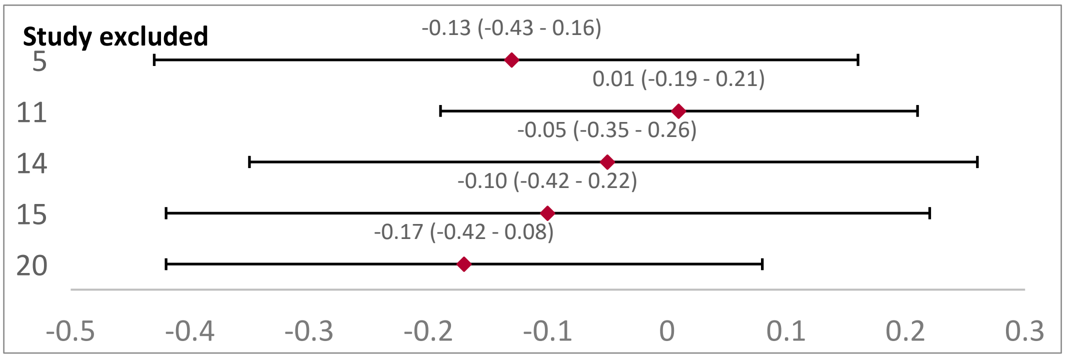

Figure S64. Sensitivity analysis for natural logarithm of rate of solicited adverse events in children aged ⩽5 years in placebo group (7 days after 2nd dose).

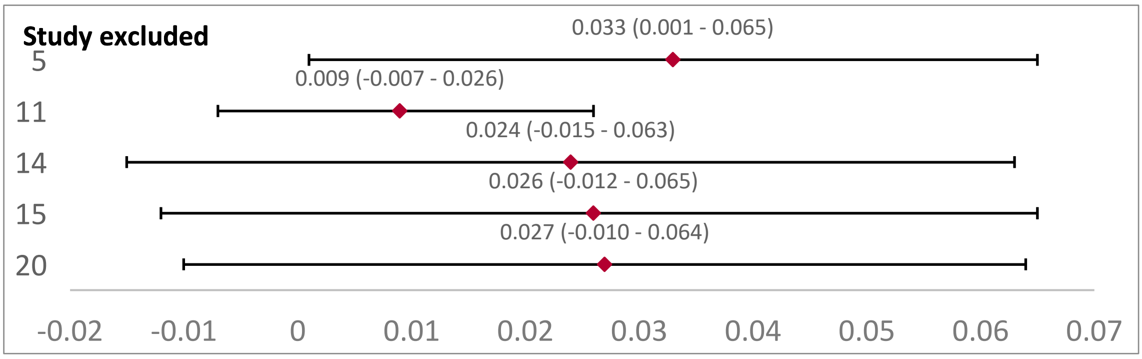

Figure S65. Sensitivity analysis for difference in rate of solicited adverse events in children aged ≤5 years between vaccine and placebo groups (7 days after 2nd dose).

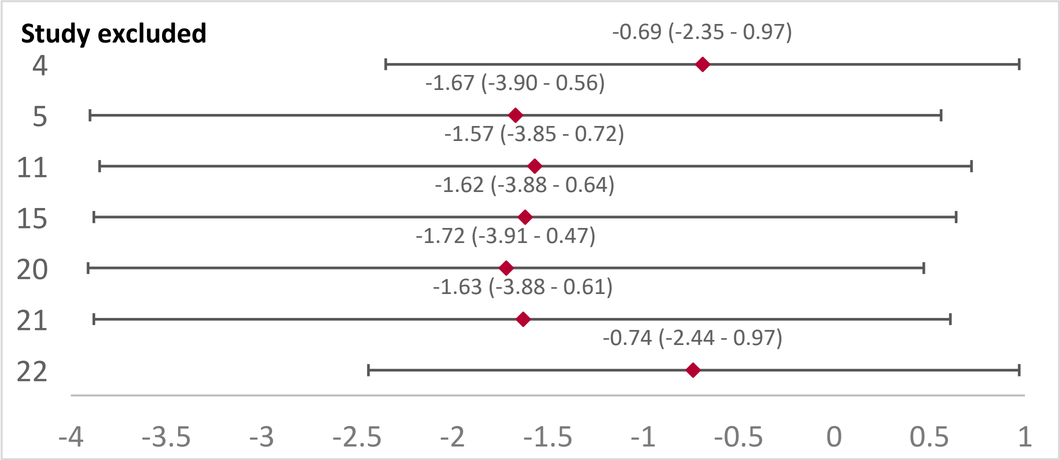

Figure S66. Sensitivity analysis for natural logarithm of rate of unsolicited adverse events in children aged ≤5 years in vaccine group (1 month after 2nd dose).

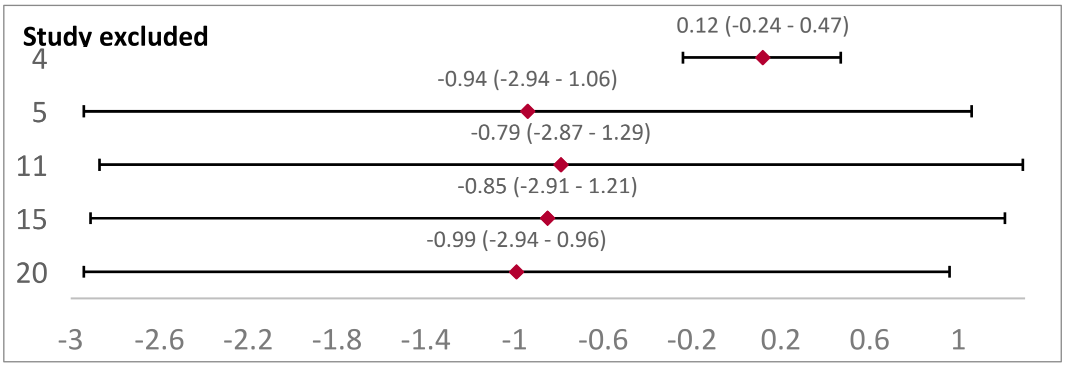

Figure S67. Sensitivity analysis for natural logarithm of rate of unsolicited adverse events in children aged ≤5 years in placebo group (1 month after 2nd dose).

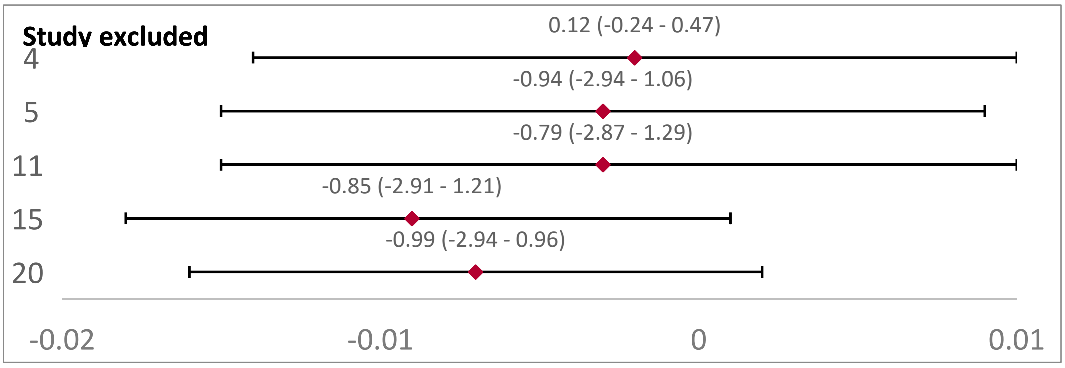

Figure S68. Sensitivity analysis for difference in rate of unsolicited adverse events in children aged ≤5 years between vaccine and placebo groups (1 month after 2nd dose).

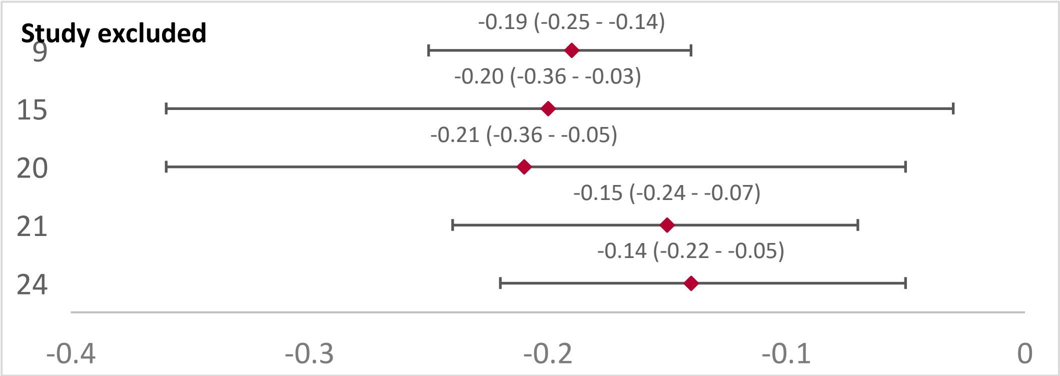

Figure S69. Sensitivity analysis for natural logarithm of rate of systemic adverse events in children aged ≤5 years in vaccine group (7 days after 2nd dose).

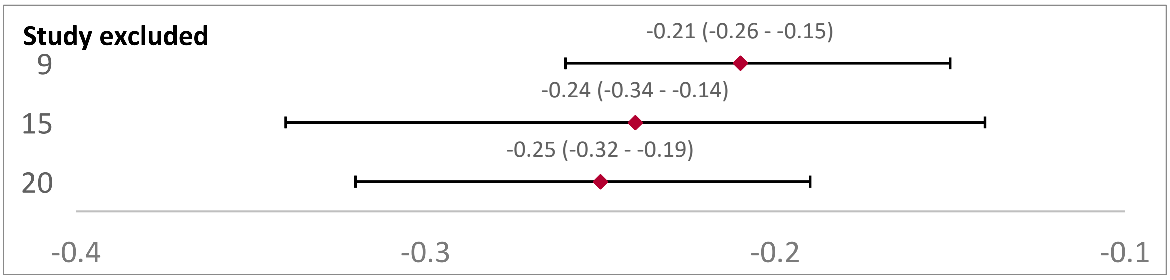

Figure S70. Sensitivity analysis for natural logarithm of rate of systemic adverse events in children aged ≤5 years in placebo group (7 days after 2nd dose).

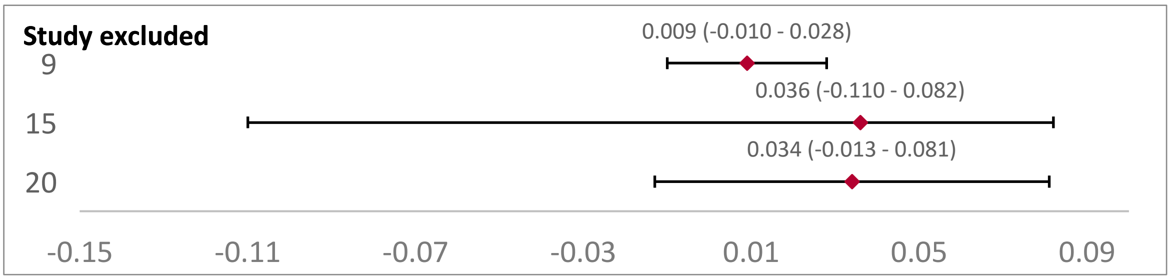

Figure S71. Sensitivity analysis for difference in rate of systemic adverse events in children aged ≤5 years in between vaccine and placebo groups (7 days after 2nd dose).

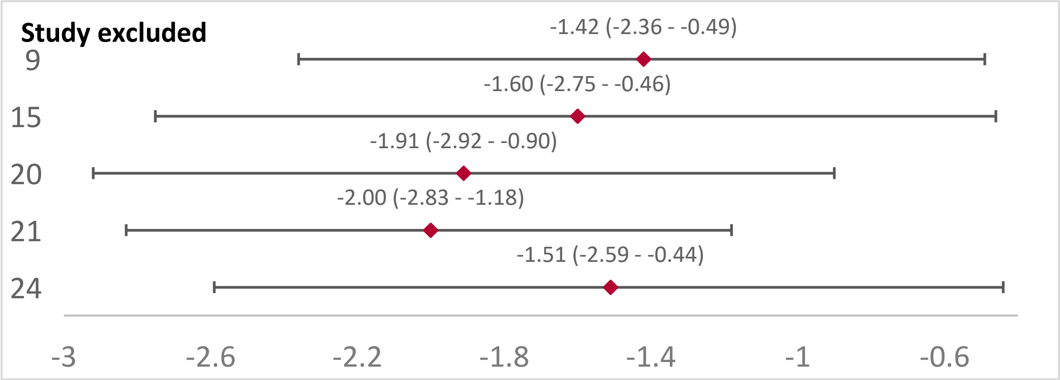

Figure S72. Sensitivity analysis for natural logarithm of rate of local adverse events in children aged ≤5 years in vaccine group (7 days after 2nd dose).

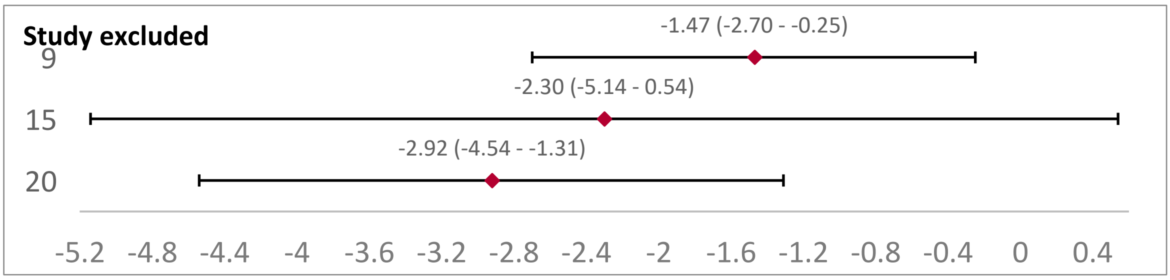

Figure S73. Sensitivity analysis for natural logarithm of rate of local adverse events in children aged ≤5 years in placebo group (7 days after 2nd dose).

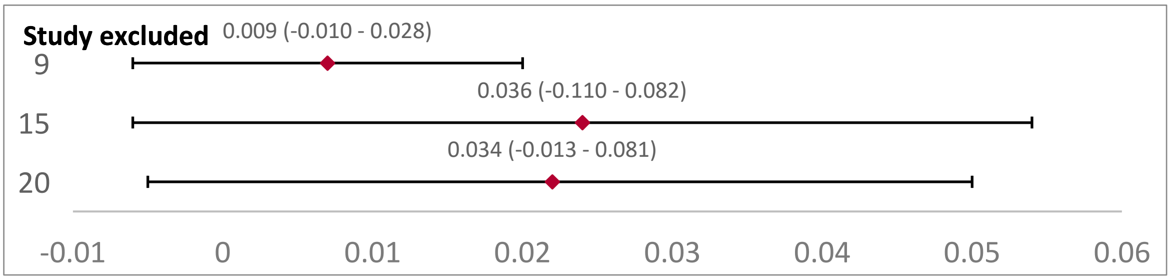

Figure S74. Sensitivity analysis for difference in rate of local adverse events in children aged ≤5 years between vaccine and placebo groups (7 days after 2nd dose).

Effect size of each study — Confidence interval of effect si
Estimated overall effect size — Overall effect size value
Estimated overall confidence interval

ID	Study	Effect Size	Std. Error	Lower	Upper	p-value	Weight	Weight (%)
5	Sinovac	2.00	0.13	1.75	2.25	0.00	0.94	13.47
12	WIBP	2.37	0.40	1.60	3.15	0.00	0.83	11.90
13	WIBP	2.96	0.31	2.35	3.56	0.00	0.88	12.53
14	WIBP	2.40	0.15	2.11	2.69	0.00	0.94	13.40
17	WIBP	3.23	0.10	3.02	3.43	0.00	0.95	13.54
22	CAMS or Sinovac	4.89	0.71	3.50	6.28	0.00	0.65	9.23
23	CAMS or Sinovac or WIBP	4.97	0.32	4.35	5.59	0.00	0.87	12.48
24	CAMS or Sinovac	2.62	0.14	2.35	2.89	0.00	0.94	13.44
	Overall	3.10	0.38	2.36	3.85	0.00		

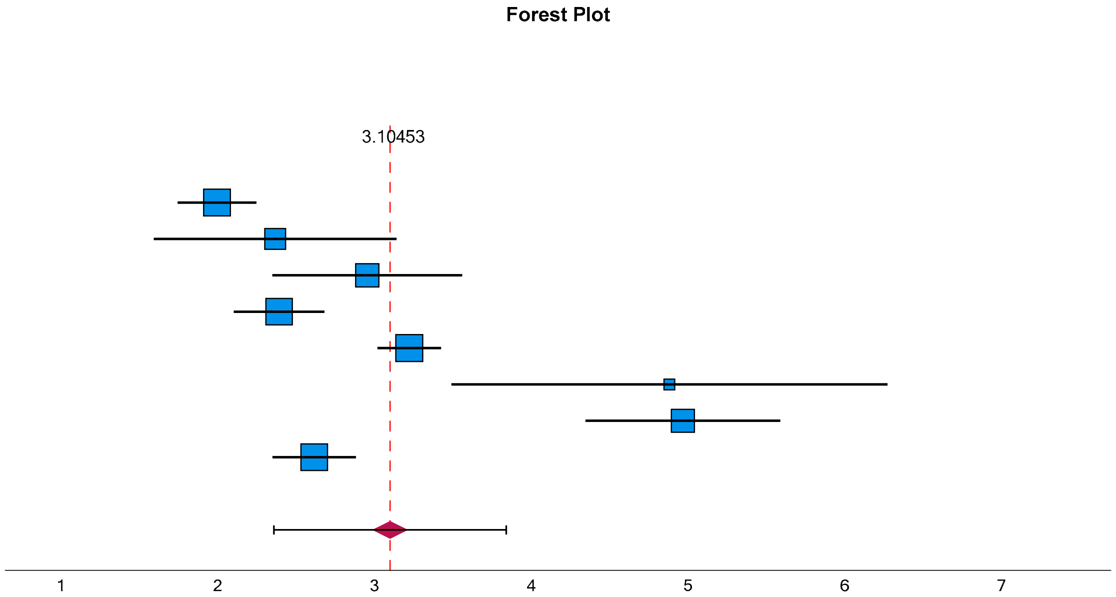

Model: Random-effects model
Heterogeneity: Tau-squared = 1.05, H-squared = 33.91, I-squared = 0.97
Homogeneity: Q = 121.53, df = 7, p-value = 0.00
Test of overall effect size: z = 8.21, p-value = 0.00

Studies excluded: 18 and 21

Figure S75. Natural logarithm of seroconversion rate of children aged ⩽5 years in vaccine group (1 month after 2nd dose) after excluding of all studies requiring continuity correction.

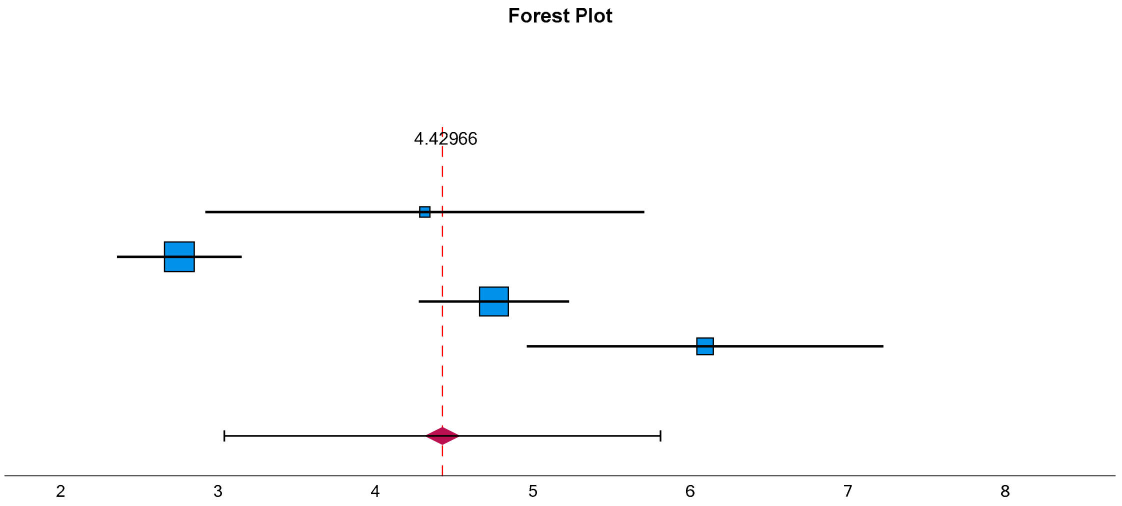

ID	Study	Effect Size	Std. Error	Lower	Upper	p-value	Weight	Weight (%)
13	WIBP	4.32	0.71	2.92	5.71	0.00	0.44	21.83
14	WIBP	2.76	0.20	2.36	3.15	0.00	0.55	27.41
17	WIBP	4.76	0.24	4.28	5.23	0.00	0.54	27.14
23	CAMS or Sinovac or WIBP	6.10	0.58	4.97	7.23	0.00	0.47	23.61
Overall		4.43	0.71	3.04	5.82	0.00		

Model: Random-effects model
Heterogeneity: Tau-squared = 1.78, H-squared = 17.00, I-squared = 0.94
Homogeneity: Q = 58.26, df = 3, p-value = 0.00
Test of overall effect size: z = 6.27, p-value = 0.00

Studies excluded: 12 and 18

Figure S76. Natural logarithm of seroconversion rate of children aged ≤5 years with seronegative baseline in vaccine group (1 month after 2nd dose) after excluding of all studies requiring continuity correction.

Effect size of each study
Estimated overall effect size
Estimated overall confidence interval
Confidence interval of effect si
Overall effect size value

ID	Study	Effect Size	Std. Error	Lower	Upper	p-value	Weight	Weight (%)
2	Sinovac	4.54	1.01	2.57	6.51	0.00	0.35	7.70
3	Sinovac	6.88	1.00	4.92	8.84	0.00	0.35	7.73
5	Sinovac	4.40	0.38	3.66	5.15	0.00	0.51	11.08
8	CAMS	3.64	0.21	3.23	4.06	0.00	0.53	11.67
9	CAMS	1.96	0.14	1.69	2.22	0.00	0.54	11.84
14	WIBP	5.70	0.71	4.31	7.09	0.00	0.43	9.39
15	WIBP	4.41	0.16	4.11	4.72	0.00	0.54	11.80
22	CAMS or Sinovac	5.58	1.00	3.62	7.55	0.00	0.35	7.72
23	CAMS or Sinovac or WIBP	6.18	0.58	5.05	7.31	0.00	0.46	10.11
25	CAMS or WIBP	5.51	0.41	4.71	6.31	0.00	0.50	10.96
	Overall	4.74	0.47	3.83	5.66	0.00		

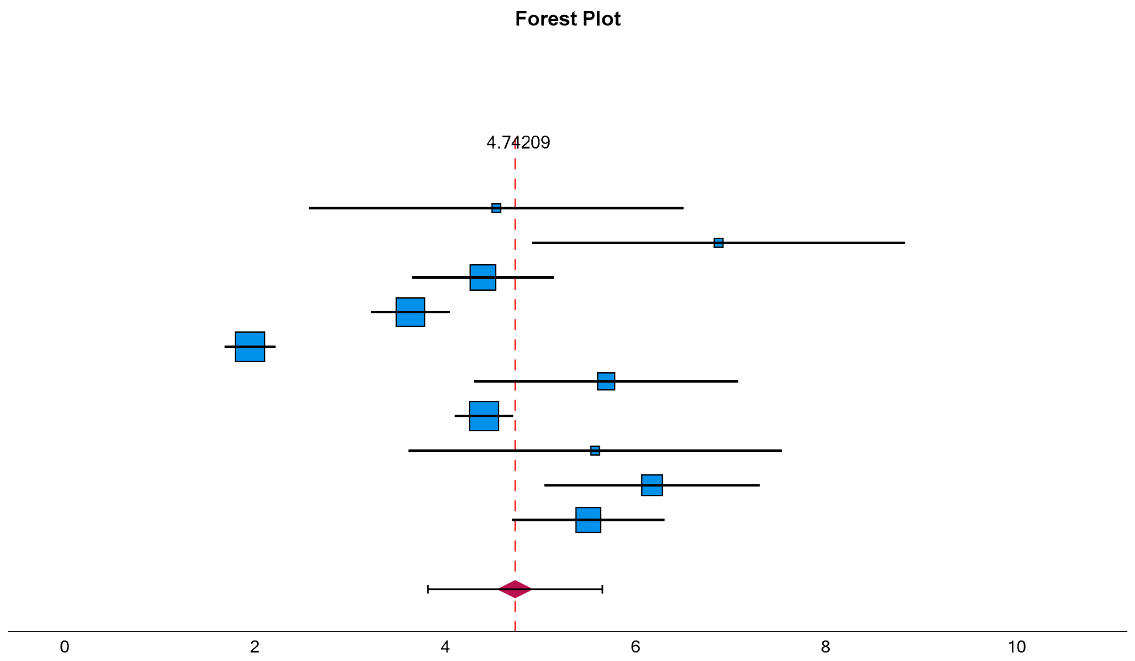

Model: Random-effects model
Heterogeneity: Tau-squared = 1.83, H-squared = 21.35, I-squared = 0.95
Homogeneity: Q = 239.45, df = 9, p-value = 0.00
Test of overall effect size: z = 10.14, p-value = 0.00

Studies excluded: 1, 12 and 24

Figure S77. Natural logarithm of proportion of children aged ≤5 years in vaccine group with antibody titre ≥1:8 (1 month after 2nd dose) after excluding of all studies requiring continuity correction.

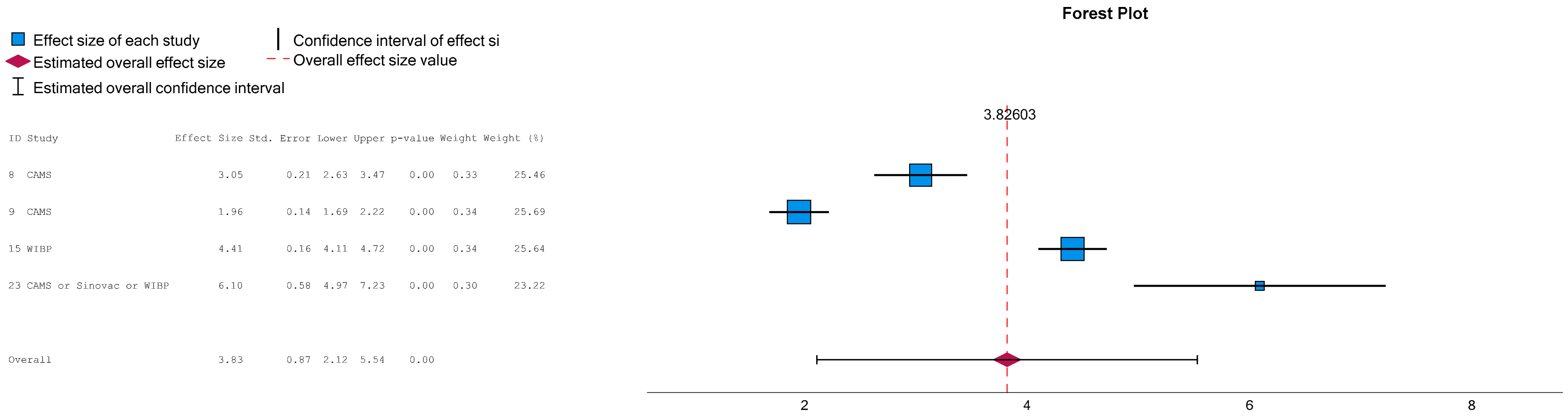

ID	Study	Effect Size	Std. Error	Lower	Upper	p-value	Weight	Weight (%)
8	CAMS	3.05	0.21	2.63	3.47	0.00	0.33	25.46
9	CAMS	1.96	0.14	1.69	2.22	0.00	0.34	25.69
15	WIBP	4.41	0.16	4.11	4.72	0.00	0.34	25.64
23	CAMS or Sinovac or WIBP	6.10	0.58	4.97	7.23	0.00	0.30	23.22
	Overall	3.83	0.87	2.12	5.54	0.00		

Model: Random-effects model
Heterogeneity: Tau-squared = 2.95, H-squared = 76.61, I-squared = 0.99
Homogeneity: Q = 167.01, df = 3, p-value = 0.00
Test of overall effect size: z = 4.38, p-value = 0.00

Study excluded: 12, 14, 24 and 25

Figure S78. Natural logarithm of proportion of children aged ≤5 years with seronegative baseline in vaccine group with antibody titre ≥1:8 (1 month after 2nd dose) after excluding of all studies requiring continuity correction.

Effect size of each study

Estimated overall effect size

Estimated overall confidence interval

Confidence interval of effect si

Overall effect size value

ID	Study	Effect Size	Std. Error	Lower	Upper	p-value	Weight	Weight (%)
4	Sinovac	1.86	0.10	1.66	2.06	0.00	0.70	17.14
5	Sinovac	2.36	0.15	2.07	2.65	0.00	0.70	17.00
14	WIBP	3.02	0.19	2.64	3.39	0.00	0.69	16.82
15	WIBP	3.23	0.09	3.06	3.41	0.00	0.70	17.17
20	Medigen	5.33	0.45	4.45	6.21	0.00	0.62	15.12
25	WIBP	4.16	0.21	3.74	4.57	0.00	0.69	16.74
Overall		3.28	0.49	2.32	4.25	0.00		

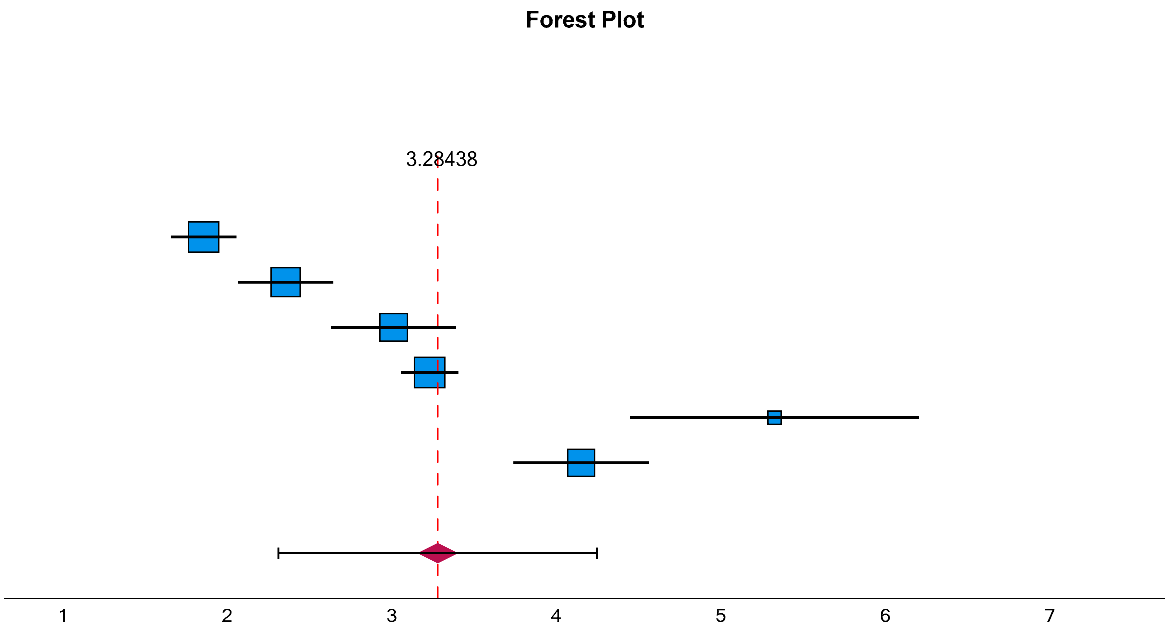

Model: Random-effects model

Heterogeneity: Tau-squared = 1.42, H-squared = 67.04, I-squared = 0.99

Homogeneity: Q = 191.92, df = 5, p-value = 0.00

Test of overall effect size: z = 6.64, p-value = 0.00

Study excluded: 18 and 21

Figure S79. Natural logarithm of proportion of children aged ≤5 years in vaccine group with antibody titre ≥1:32 (1 month after 2nd dose) after excluding of all studies requiring continuity correction.

Effect size of each study
Estimated overall effect size
Estimated overall confidence interval
Confidence interval of effect si
Overall effect size value

ID	Study	Effect Size	Std. Error	Lower	Upper	p-value	Weight	Weight (%)
1	Placebo	-3.14	1.02	-5.14	-1.13	0.00	0.39	8.47
3	Placebo	-5.16	0.71	-6.55	-3.77	0.00	0.49	10.72
5	Placebo	-4.71	0.15	-5.00	-4.41	0.00	0.64	14.03
9	Placebo	-3.84	0.09	-4.02	-3.67	0.00	0.65	14.16
13	Placebo	-4.08	0.71	-5.48	-2.68	0.00	0.49	10.69
14	Placebo	-5.28	0.20	-5.66	-4.89	0.00	0.64	13.89
15	Placebo	-5.10	0.21	-5.52	-4.68	0.00	0.63	13.83
20	Placebo	-1.70	0.07	-1.83	-1.56	0.00	0.65	14.19
Overall		-4.14	0.47	-5.05	-3.22	0.00		

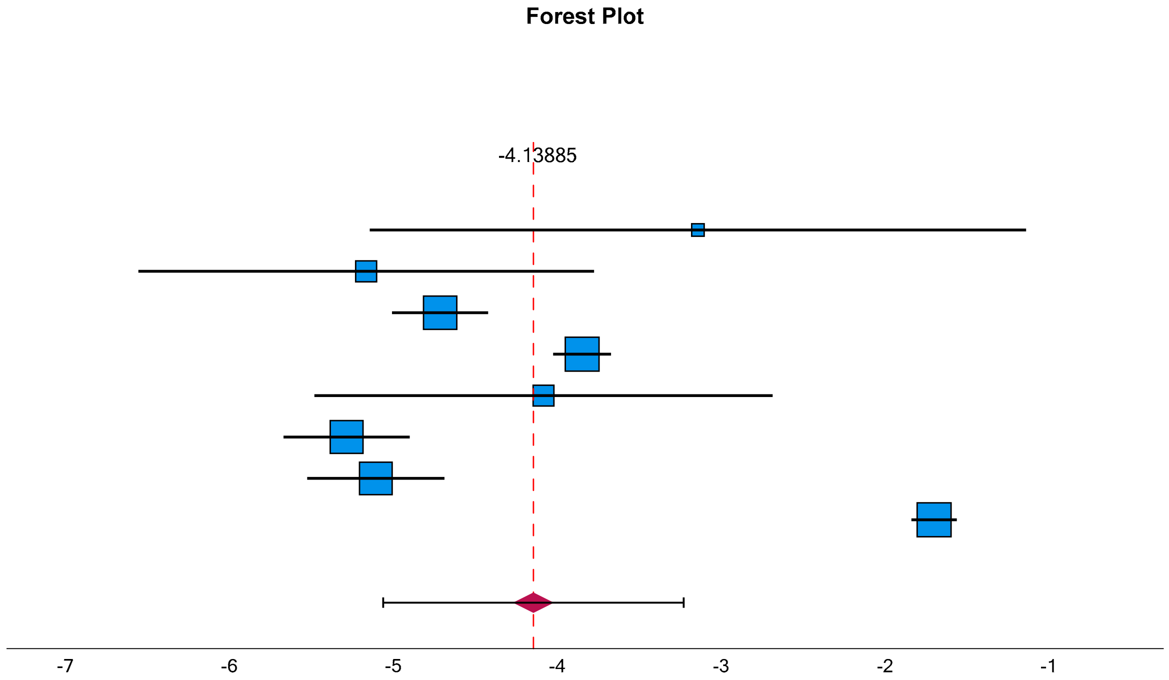

Model: Random-effects model
Heterogeneity: Tau-squared = 1.53, H-squared = 62.79, I-squared = 0.98
Homogeneity: Q = 798.66, df = 7, p-value = 0.00
Test of overall effect size: z = -8.86, p-value = 0.00

Study excluded: 2 and 4

Figure S80. Natural logarithm of rate of serious adverse events in children aged ≤5 years in placebo group (1 month after 2nd dose) after excluding of all studies requiring continuity correction.

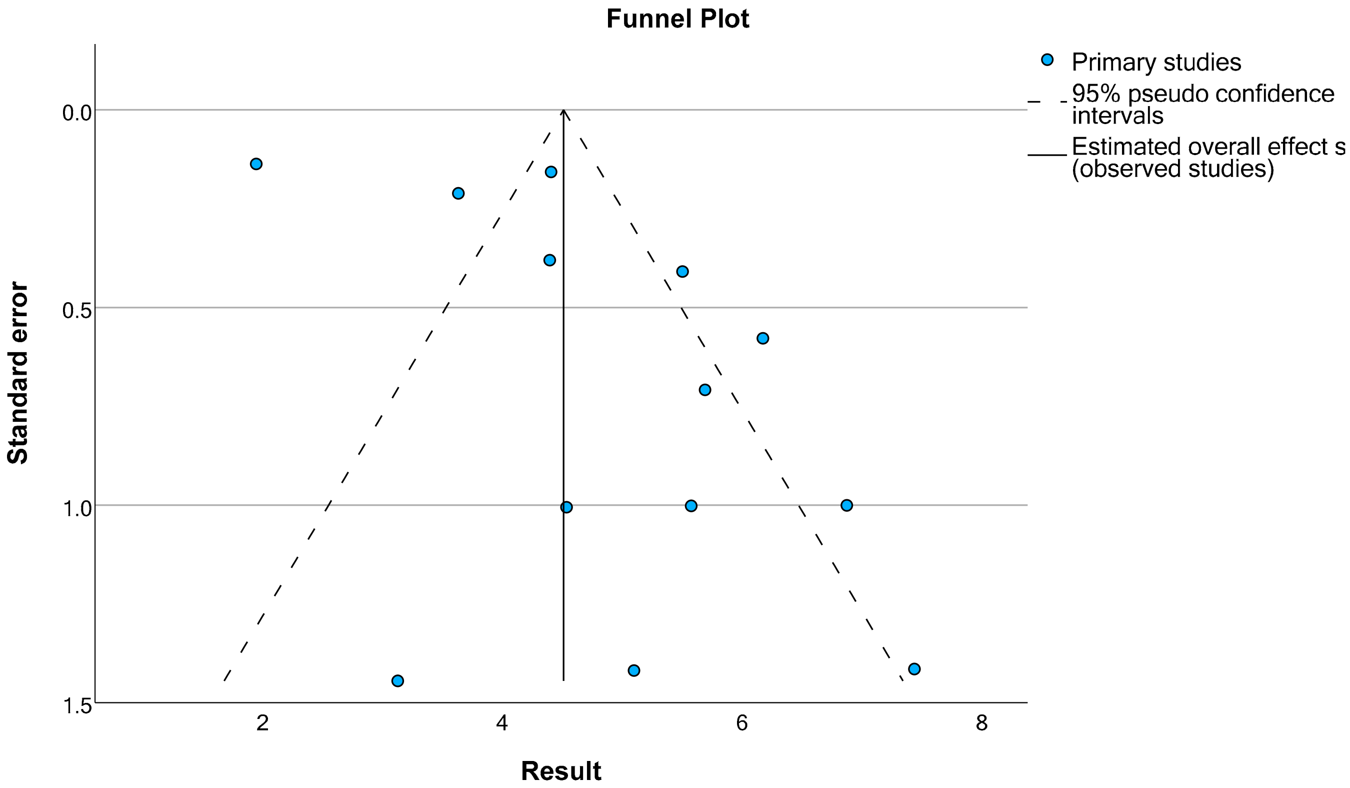

Figure S81. Funnel plot for natural logarithm of proportion of children aged ⩽5 years in vaccine group with antibody titre ⩾1:8 (1 month after 2nd dose).

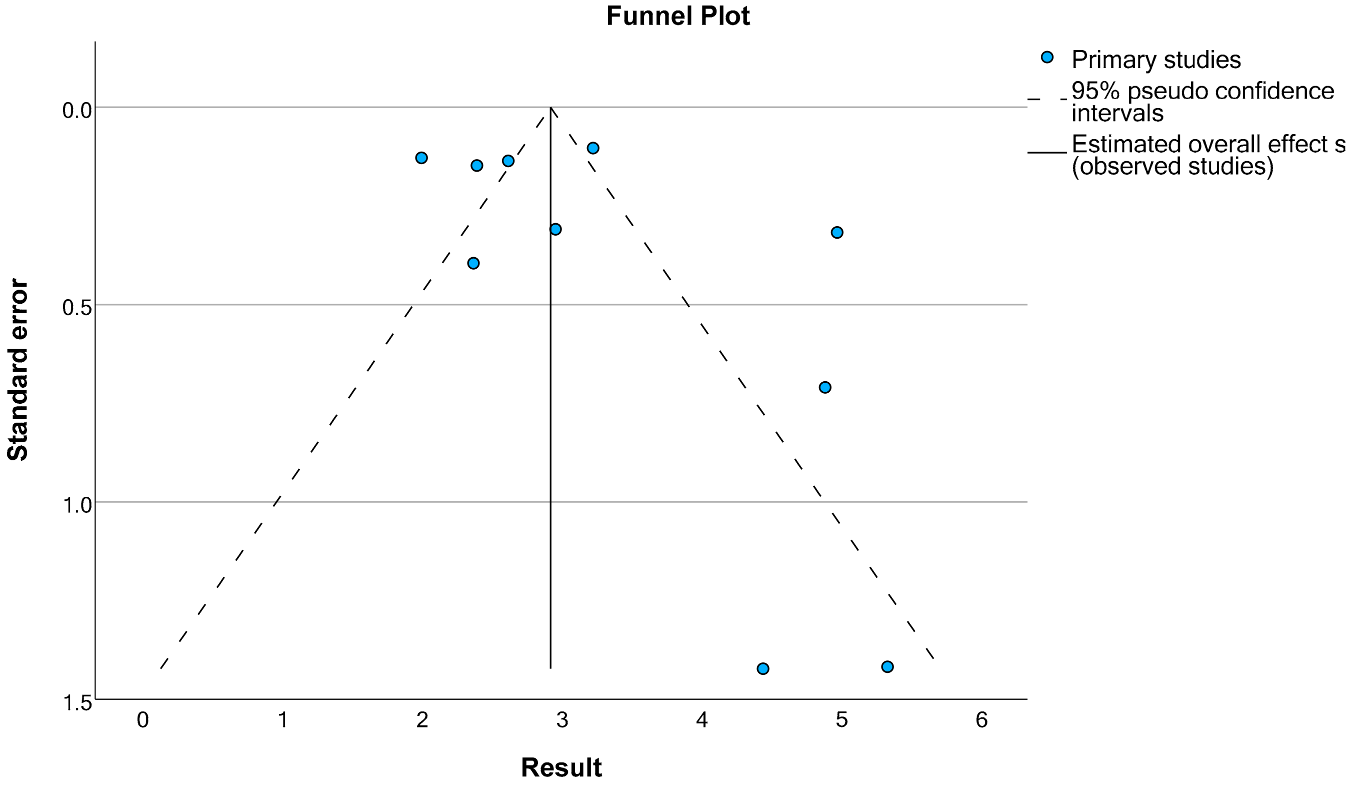

Figure S82. Funnel plot for natural logarithm of seroconversion rate of children aged ⩽5 years in vaccine group (1 month after 2nd dose).

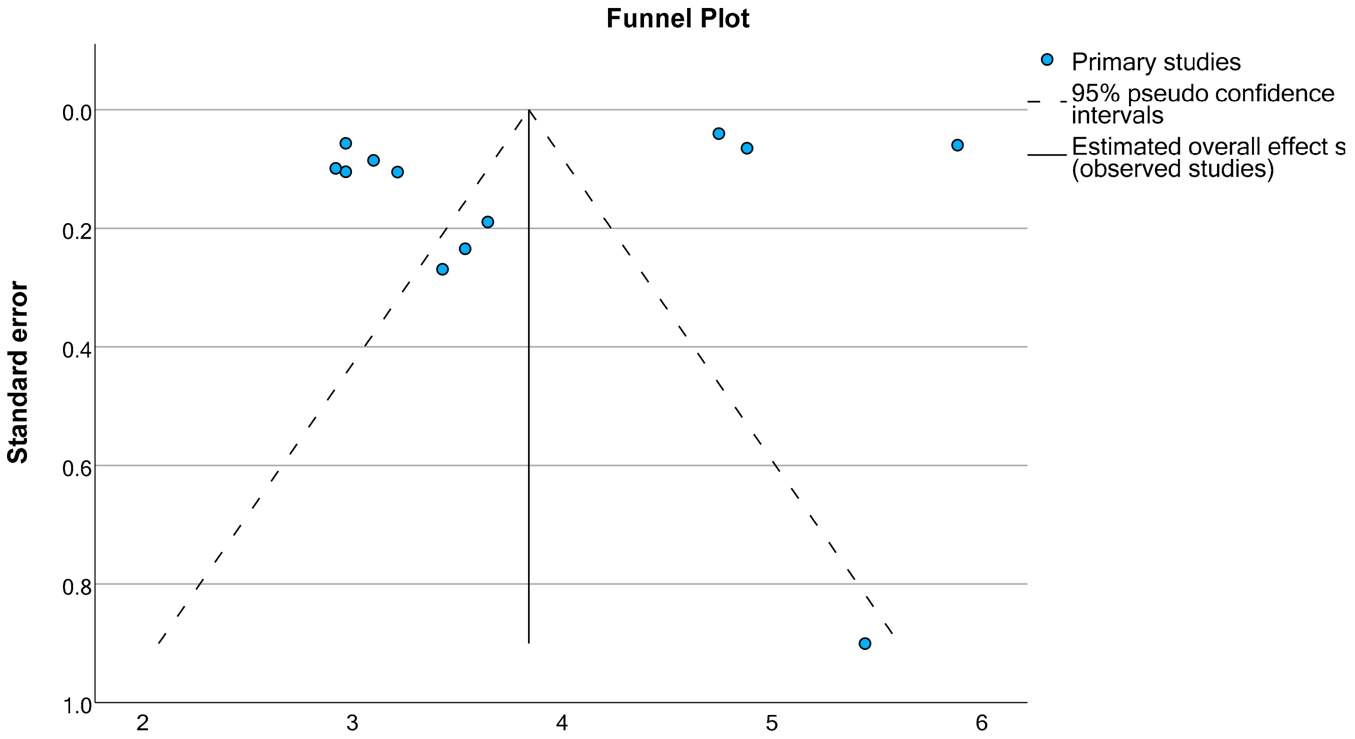

Figure S83. Funnel plot for natural logarithm of ratio of GMT between vaccine and placebo groups in children aged ⩽ 5 years (1 month after 2nd dose).

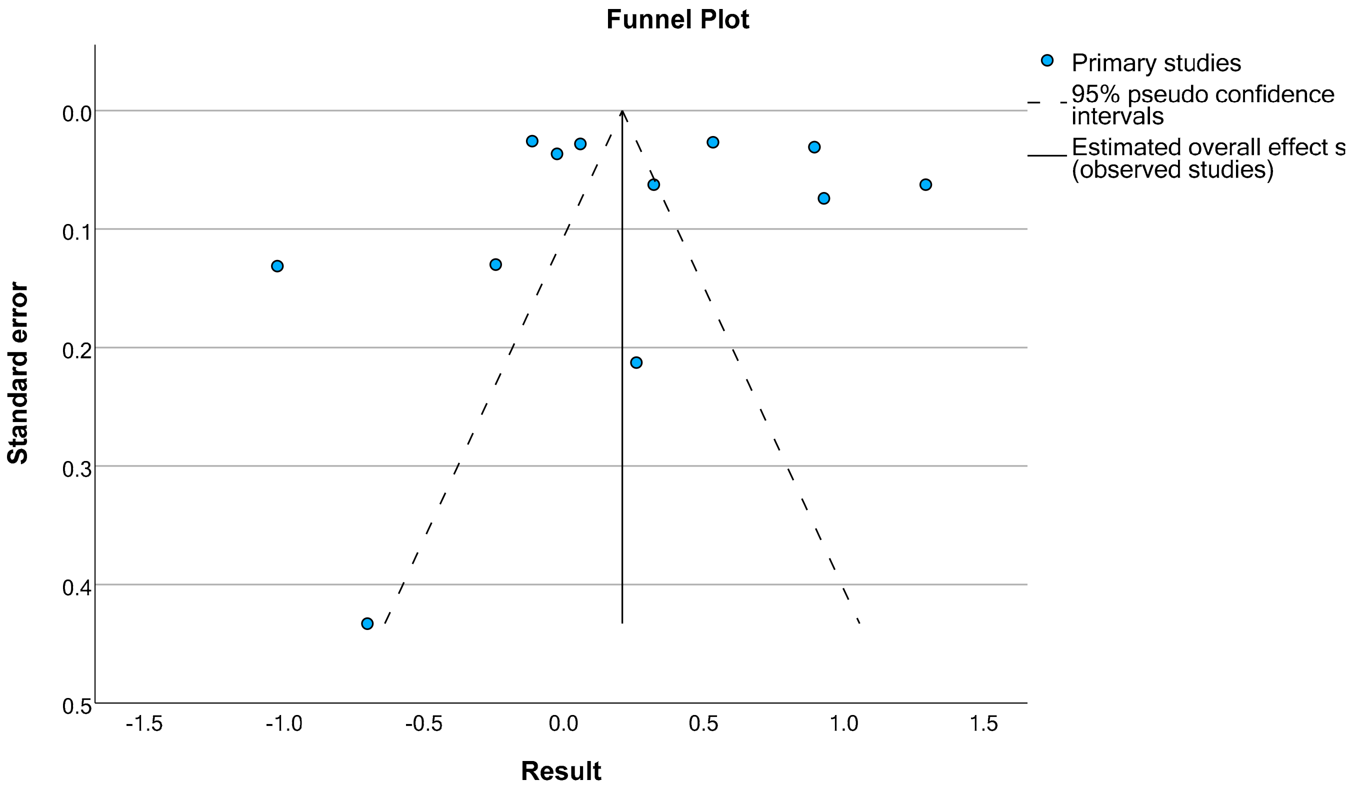

Figure S84. Funnel plot for natural logarithm of rate of any adverse events in children aged ⩽5 years in vaccine group (1 month after 2nd dose).

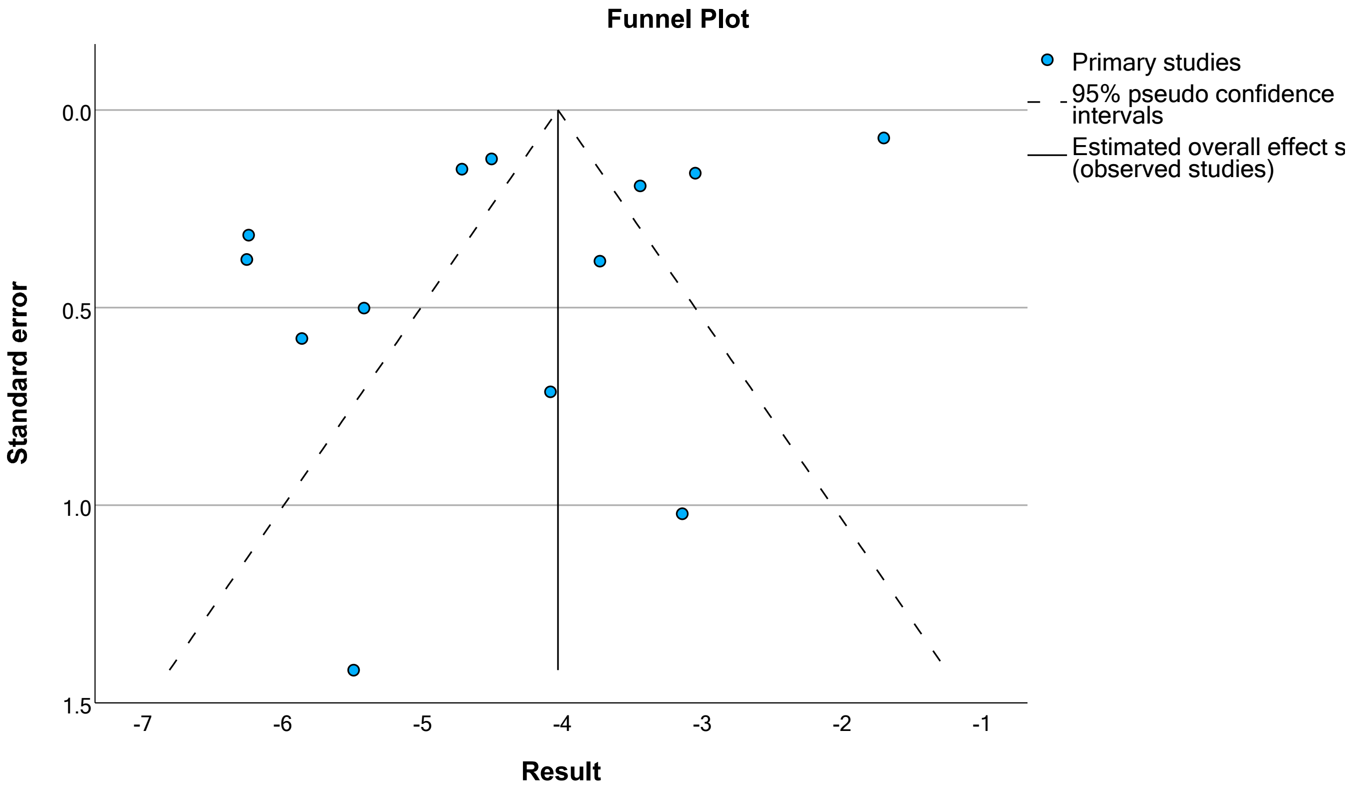

Figure S85. Funnel plot for natural logarithm of rate of serious adverse events in children aged ≤5 years in vaccine group (1 month after 2nd dose).

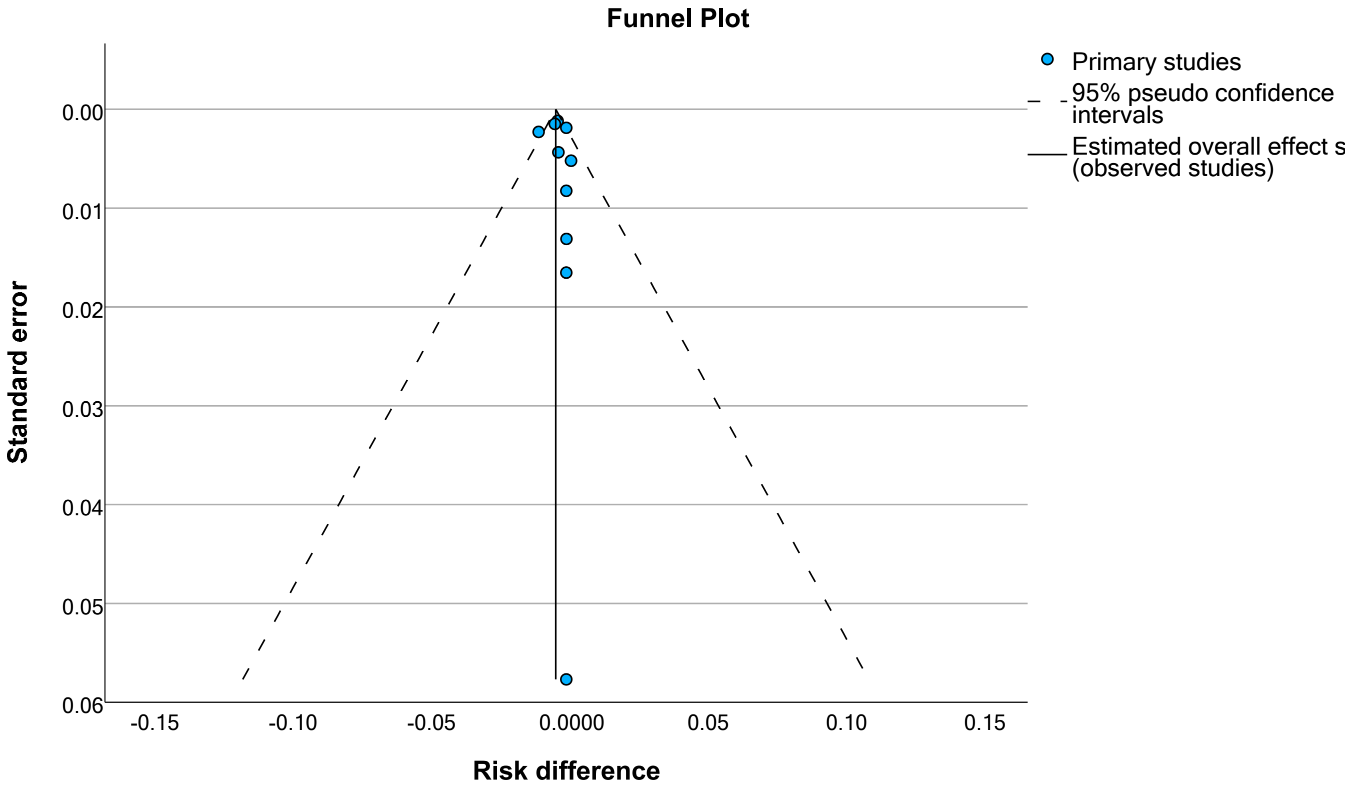

Figure S86. Funnel plot of difference in rate of serious adverse events in children aged ≤5 years between vaccine and placebo groups (1 month after 2nd dose).

Table S2. Egger's test result.

Outcome	Coefficient	Standard Error	T	P	Lower 95% CI	Upper 95% CI
Natural logarithm of seropositive rate (NAbT ≥1:8) at 1 month (vaccine group)	3.72	0.64	5.85	<0.001	2.32	5.13
Natural logarithm of seroconversion rate at 1 month (vaccine group)	2.53	0.43	5.86	<0.001	1.53	3.53
Natural logarithm of ratio of GMT between vaccine and placebo groups at 1 month	3.66	0.40	9.15	<0.001	2.77	4.55
GMT at 1 month among those with seronegative baseline (vaccine group)	5.52	0.26	21.08	<0.001	4.93	6.11
Natural logarithm of rate of any AE at 1 month (vaccine group)	0.49	0.25	1.99	0.075	-0.06	1.04
Natural logarithm of rate of serious AE at 1 month (vaccine group)	-3.93	0.63	-6.21	<0.001	-5.32	-2.54
Difference in rate of serious adverse events between vaccine and placebo groups at 1 month	0.00	0.00	-2.13	0.07	-0.01	0.00
